# FtsZ phosphorylation pleiotropically affects Z-ladder formation, antibiotic production, and morphogenesis in *Streptomyces coelicolor*

**DOI:** 10.1007/s10482-022-01778-w

**Published:** 2022-11-16

**Authors:** Paula Yagüe, Joost Willemse, Xiansha Xiao, Le Zhang, Angel Manteca, Gilles P. van Wezel

**Affiliations:** 1 Department of Molecular Biotechnology, Institute of Biology Leiden, Leiden University, PO Box 9505, 2300 AB Leiden, The Netherlands; 2 Departamento de Biología Funcional e IUOPA, Área de Microbiología, Facultad de Medicina, Universidad de Oviedo, 33006 Oviedo, Spain

**Keywords:** Cell division, Differentiation, Serine-phosphorylation, Sporulation, *Streptomyces*

## Abstract

The GTPase FtsZ forms the cell division scaffold in bacteria, which mediates the recruitment of the other components of the divisome. Streptomycetes undergo two different forms of cell division. Septa without detectable peptidoglycan divide the highly compartmentalised young hyphae during early vegetative growth, and cross-walls are formed that dissect the hyphae into long multinucleoid compartments in the substrate mycelium, while ladders of septa are formed in the aerial hyphae that lead to chains of uninucleoid spores. In a previous study, we analysed the phosphoproteome of *Streptomyces coelicolor* and showed that FtsZ is phosphorylated at Ser 317 and Ser389. Substituting Ser–Ser for either Glu–Glu (mimicking phosphorylation) or Ala–Ala (mimicking non-phosphorylation) hinted at changes in antibiotic production. Here we analyse development, colony morphology, spore resistance, and antibiotic production in FtsZ knockout mutants expressing FtsZ alleles mimicking Ser319 and Ser387 phosphorylation and non-phosphorylation: AA (no phosphorylation), AE, EA (mixed), and EE (double phosphorylation). The FtsZ-eGFP AE, EA and EE alleles were not able to form observable FtsZ-eGFP ladders when they were expressed in the *S. coelicolor* wild-type strain, whereas the AA allele could form apparently normal eGFP Z-ladders. The FtsZ mutant expressing the FtsZ EE or EA or AE alleles is able to sporulate indicating that the mutant alleles are able to form functional Z-rings leading to sporulation when the wild-type FtsZ gene is absent. The four mutants were pleiotropically affected in colony morphogenesis, antibiotic production, substrate mycelium differentiation and sporulation (sporulation timing and spore resistance) which may be an indirect result of the effect in sporulation Z-ladder formation. Each mutant showed a distinctive phenotype in antibiotic production, single colony morphology, and sporulation (sporulation timing and spore resistance) indicating that the different FtsZ phosphomimetic alleles led to different phenotypes. Taken together, our data provide evidence for a pleiotropic effect of FtsZ phosphorylation in colony morphology, antibiotic production, and sporulation.

**Supplementary Information:**

The online version contains supplementary material available at 10.1007/s10482-022-01778-w.

## Introduction

Streptomycetes are filamentous bacteria with a complex multicellular lifecycle (Claessen et al. 2014). After germination, the hyphae grow out via tip growth and branching, forming an intricate network called the vegetative mycelium. This vegetative or substrate mycelium undergoes several rounds of programmed cell death, which is required for the formation of the reproductive aerial hyphae (Manteca et al. 2006; Yague et al. 2016). Eventually, the aerial hyphae differentiate into chains of spores (Flardh and Buttner 2009). The biochemical pathways regulating this complex way of growth have not yet been fully characterized (Claessen et al. 2006; McCormick and Flardh 2012). Streptomycetes produce a wide range of bioactive metabolites that are important for medicine, biotechnology, and agriculture (Barka et al. 2016; Berdy 2005; Katz and Baltz 2016). They are referred to as nature’s medicine makers (Hopwood 2007) and play a key role in the protection of eukaryotic hosts against challenges by pathogens (van Bergeijk et al. 2020). The production of secondary metabolites is closely linked to the developmental program of streptomycetes (van der Heul et al. 2018). Understanding the regulatory networks that control the physiology of this intriguing bacterium is one of the keys to understanding and improving secondary metabolism activation (Manteca and Yague 2018).

Bacterial cell division has been studied extensively, whereby much information has been obtained particularly from the rod-shaped bacteria *Escherichia coli* or *Bacillus subtilis* (Du and Lutkenhaus 2017; Errington and Wu 2017). These bacteria grow by elongation of the lateral wall and divide by binary fission (Koch 2000). The process involves more than 35 proteins that form the so-called divisome at mid-cell. FtsZ is a tubulin-like GTPase protein (de Boer et al. 1992) that polymerizes into protofilaments that together form the Z-ring (Sun and Margolin 1998). The Z-ring is a cytoskeletal structure that forms the scaffold at the site of division and creates the constricting force that eventually divides the cell into two daughter cells (Vicente et al. 2006). The formation of the Z-ring is regulated negatively by the Min proteins that prevent division at the cell poles (Szwedziak and Ghosal 2017; Howard 2004), and nucleoid occlusion to prevent the formation of the septum over non-segregated chromosomes (Margolin 2004; Wu and Errington 2004). Various FtsZ-interacting proteins have been discovered that often interact with the conserved C-terminal end of the protein, showing that this is a hotspot for protein interactions (Huang et al. 2016). These interactions play a major role in the polymerization and correct localization of the Z-ring. The conserved C-terminal part (CCTP) of FtsZ interacts among others with the membrane tethers FtsA and ZipA (Hale and de Boer 1997; Pichoff and Lutkenhaus 2002), the Z-ring stabilising proteins ZapA (Low et al. 2004) and ZapD (Durand-Heredia et al. 2012), and with SepF, which is the only one of these proteins conserved in *Streptomyces* and forms ring-like structures that promote FtsZ polymerization (Hamoen et al. 2006; Krol et al. 2012; Singh et al. 2008). Conversely, the FtsZ-recruiting SsgB in *Streptomyces* interacts with the N-terminal part of FtsZ (Willemse et al. 2011). The precise FtsZ dynamics in bacterial cell division and the identity of the divisome proteins are still not fully understood (reviewed in (Barrows and Goley 2021).

Cell division in the mycelial streptomycetes is coordinated differently, involving two types of cell division (Jakimowicz and van Wezel 2012). During vegetative growth, cell division results in cross-walls that divide the hyphae into long multinucleoid syncytial cells. Complex membrane assemblies thereby form chromosome-free zones in the hyphae during septum formation, apparently protecting the DNA from damage during division (Celler et al. 2016). During sporulation-specific cell division in the aerial hyphae, up to a hundred septa are laid down more or less simultaneously, eventually resulting in the formation of chains of uninucleoid spores (Jakimowicz and van Wezel 2012). Vegetative and sporulation-specific cell division also differ mechanistically, as illustrated by the fact that many cell division genes that are required for sporulation (e.g. *ftsI*, *ftsL,* and *ftsW*) are dispensable for vegetative cross-wall formation (Bennett et al. 2007, 2009; Mistry et al. 2008; Yague et al. 2016). Indeed, canonical cell division involving the divisome is only seen during sporulation. While in most bacteria cell division is negatively controlled, in streptomycetes FtsZ is actively recruited by the SsgB protein (Willemse et al. 2011). In turn, the localization of SsgB depends on its orthologue SsgA (Traag and van Wezel 2008), while SsgB is tethered to the membrane by SepG (Zhang et al. 2016). Thus the control of cell division differs substantially from rod-shaped bacteria, most likely due to the absence of a mid-cell reference (Jakimowicz and van Wezel 2012). FtsZ is essential in most bacteria, but surprisingly, *ftsZ* mutants of *Streptomyces* are viable (McCormick et al. 1994; Dai and Lutkenhaus 1991)*.* Dynamin-like proteins DynA and DynB were reported to stabilise Z-rings during *Streptomyces* sporulation (Schlimpert et al. 2017). Recent works discovered novel proteins associated with *Streptomyces* FtsZ during the vegetetative (Bush et al. 2022) and sporulation divisomes (Ramos-Leon et al. 2021). FtsZ is also involved in the formation of septa without detectable peptidoglycan in the highly compartmentalised young *Streptomyces* hyphae formed after spore germination and preceding the differentiation of the multinucleated substrate mycelium (Yague et al. 2016), making *Streptomyces* cell division even more complex.

Besides the various layers of transcriptional regulation that control the major processes in cells, posttranslational modifications (PTMs) also play a major role. Protein phosphorylation is one of the most important PTMs in cells. His/Asp phosphorylation is a well-known PTM in prokaryotes, since it forms part of the two-component systems, while Ser/Thr/Tyr phosphorylation is one of the most important PTMs in eukaryotes (Petrickova and Petricek 2003). However, Ser/Thr/Tyr phosphorylation also exists in bacteria, where it has important regulatory roles, though it is still less well understood than in eukaryotes (Yague et al. 2019; Pereira et al. 2011; Petrickova and Petricek 2003). Members of the genus *Streptomyces* have one of the largest phosphoproteomes described to date (Parker et al. 2010; Manteca et al. 2011; Rioseras et al. 2018). The *S. coelicolor* genome encodes some 34 Serine Threonine Kinase proteins (STKs) and at least 184 phosphoproteins (Hempel et al. 2012; Parker et al. 2010; Rioseras et al. 2018; Petrickova and Petricek 2003; Manteca et al. 2011; Hirakata et al. 2019). To date, the biological relevance of *Streptomyces* Ser/Thr/Tyr protein phosphorylation was only experimentally validated for DivIVA, an essential protein that controls polar growth and hyphal branching (Hempel et al. 2012) and DnaA, also an essential protein controlling DNA replication (Lebkowski et al. 2020). DivIVA was also reported to be modulated by phosphorylation in *Streptococcus suis* (Ni et al. 2018). Important cellular bacterial processes were reported to be modulated by Serine/Threonine/Tyrosine phosphorylation as cell-wall remodelling, quorum sensing or bacterial virulence [reviewed in Yague et al. (2019)]. The activity of FtsZ was reported to be modulated by S/T/Y phosphorylation in some bacteria as *Deinococcus radiodurans* (Maurya et al. 2018) and *Mycobacterium tuberculosis* (Thakur and Chakraborti 2006). Despite this knowledge, much more work will be necessary to fully characterise and understand the biological role of *Streptomyces* phosphoproteome and other bacterial phosphoproteomes.

**Fig. 1:**
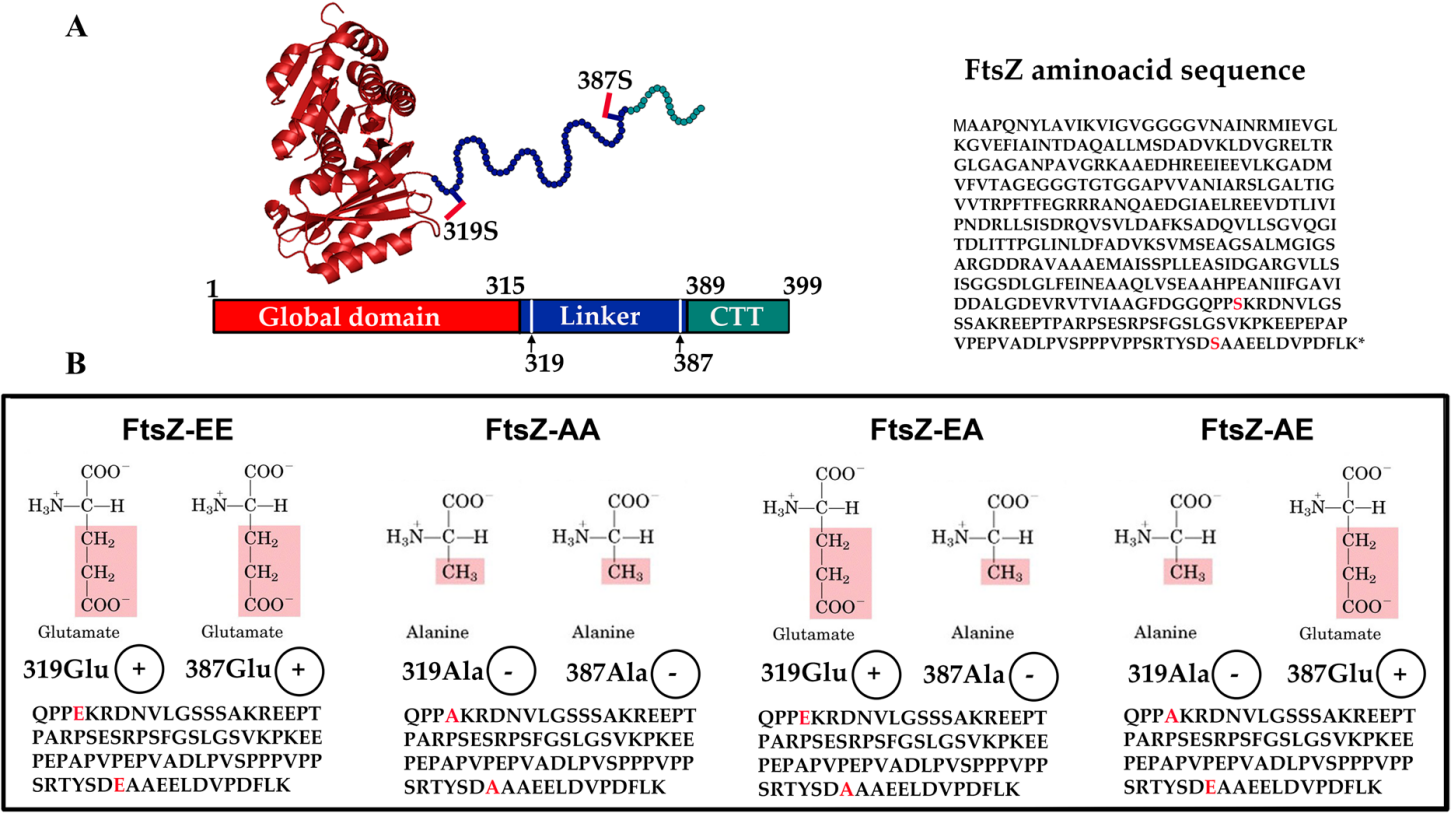
Scheme of FtsZ and the two serine sites object of this study. **A** The amino acid sequence of FtsZ protein. The cartoon represents the FtsZ core structure (in red) from *Mycobacterium tuberculosis* (PDB ID 2Q1X). The linker region of *Streptomyces coelicolor* FtsZ spans residues 315–389 (in blue), while the C-terminal tail (CTT) spans residues 389–399 (in green), which are all depicted by balls. The side chain of Ser319 and Ser387 are labeled in red sticks. **B** FtsZ amino acid substitutions in the mutants. Substitutions of the 319 and 387 serine sites are highlighted in red. + (Glu) mimics permanent phosphorylation; − (Ala) mimics permanent lack of phosphorylation

In a previous shotgun quantitative phosphoproteomic study, we discovered 131 phosphoproteins in *S. coelicolor*, one of which was FtsZ (Manteca et al. 2011; Rioseras et al. 2018). To investigate the importance and biological relevance of the FtsZ phosphorylations, we mutated residues Ser319 and Ser387 simultaneously, creating mutants mimicking FtsZ double phosphorylation (FtsZ-EE) and non-phosphorylation (FtsZ-AA). Preliminary analysis revealed that these mutations had an effect on secondary metabolism (Rioseras et al. 2018). In the current work, we further analyse the biological effect of FtsZ phosphorylation in the FtsZ-EE and FtsZ-AA mutants and in two new mutants mimicking single FtsZ phosphorylation at Ser319 or Ser387 (Mutants FtsZ-EA and FtsZ-AE) (Fig. 1). We discovered that, in addition to secondary metabolism, FtsZ phosphorylation shows a surprising pleiotropic phenotype affecting Z-ladder formation during sporulation, colony morphogenesis, sporulation timing, spore morphology, and spore resistance. To the best of our knowledge, this is the first time that serine phosphorylation was described to interfere with FtsZ polymerisation and to affect biological processes different from secondary metabolism.

## Material and methods

### Bacterial strains and media

*Streptomyces coelicolor* A3(2) M145 was obtained from the John Innes Centre (UK) strain collection (Kieser 2000). *Streptomyces coelicolor* M145 was the parent for ftsZ* mutant strains, FtsZ-EE (pGlu319 and pGlu387; EE), FtsZ-AA (pGlu319 and Ala387; EA), FtsZ-EA (Ala387 and pGlu387; AE), and FtsZ-AE (Ala319 and Ala387; AA). *Escherichia coli* ET dam^−^ 123,567 harbouring the conjugative plasmid pUZ8002 (Paget et al. 1999) was used as host for conjugation. SFM (soya flavour, mannitol) agar medium (Hobbs 1989) was used as a sporulation medium and for the study of phenotypes using scanning electron microscopy, transmission electron microscopy or stereo microscopy. GYM medium (glucose 5 g/l, yeast extract 4 g/l, malt extract 5 g/l, 0.5 g/l MgSO_4_·7H_2_O, agar 20 g/l and supplemented to a final concentration of 0.5 g/l K_2_HPO_4_) with cellophane disks was used as the growth medium for the confocal observations. MM (agar minimal medium) (Hopwood 1967), with mannitol as a carbon source, was used as a growth medium for checking the production of pigments (antibiotics) and the capacity of sporulation. For *Streptomyces*, agar plates were inoculated with 100 ml of a suspension of 10^8^ spores per ml, followed by incubation at 30 °C. For *E. coli*, Lysogen broth [LB, (Bertani 2004)] liquid/solid medium supplemented with 20% of glucose was used followed by incubation at 37 °C.

*E. coli* strains harbouring the pBluescript II SK + and pNG3 plasmids were grown in ampicillin (100 μg/ml) amended media. Nalidixic acid (25 μg/ml) was used in the *E. coli*/*Streptomyces* conjugation to inhibit *E. coli* (Kieser 2000). *Streptomyces* strains harbouring the integrative pNG3 plasmid (Gonzalez-Quinonez et al. 2016) were grown in SFM supplemented with apramycin (25 μg/ml) for sporulation. In order to prevent antibiotic interferences, the phenotypic analyses of the strains expressing the FtsZ alleles cloned into pNG3 were performed without antibiotic.

### Construction of ftsZ* mutant strains

Different versions of recombinant ftsZ* whereby the codons for Ser319 and Ser387 were replaced by codons for either glutamate or alanine or combinations thereof were synthetized by GeneCust (www.genecust.com) and cloned into pBluescript II SK + . For this, DNA fragments were amplified by PCR from the *S. coelicolor* M145 chromosome using primers: FtsZ_F and FtsZ_R (Table 1). Integrative vector pNG3 (Gonzalez-Quinonez et al. 2016) or its derivatives pNG3-EE, -EA, -AE, and -AA harbouring a single copy of each recombinant ftsZ* gene (expressed from the native *ftsZ* promoter regions described in (Flardh et al. 2000) (Table 1) were introduced into *S. coelicolor* M145 via conjugation using *E. coli* ET12567/pUZ8002 as a donor strain and following the protocol described in Kieser et al. (2000). This generated strains harbouring control plasmid or expressing FtsZ-EE, -EA, -AE, or -AA. Subsequently, the native *ftsZ* gene was inactivated using Crispr-Cas9 methodology in the four different trans-conjugants (FtsZ-EE, FtsZ-AA, FtsZ-EA, FtsZ-AE) eliminating the *ftsZ* ORF and generating the same *ftsZ* null background in all the phosphorylation/non-phosphorylation mimicking mutants (Tong et al. 2015). In the same way, a control strain harbouring *ftsZ* in pNG3 plasmid and subsequently inactivating the native copy, was performed demonstrating that the phenotypes of the mutants are not due to *ftsZ* placement (Supplementary Fig. 1A). For Crispr-Cas9 the target sequence CGATGACTTTGATGACTGCG was used (provided by http://staff.biosustain.dtu.dk/laeb/crispy_scoeli), the primer sgRNA-F (NcoI) and the primer sgRNA-R (SnaBI) (Table 1). For the overlapping extremes (recombining fragment) the following primers were used to allow homologous region replacement, as described (Tong et al. 2015): 2082LeftF, 2082LeftR, 2082RightF, 2082RightR (Table 1). This resulted in the deletion of 1144 bp of *ftsZ* (nt positions 2,234,455–2,235,599) from the *S. coelicolor* genome (Tong et al. 2015). As a control, wild type strain in this study is harbouring empty plasmid pNG3 (Table 1).

**Table 1:** Bacterial strains, plasmids and primers used in this study

	Description	References
*Bacterial strains*		
*Escherichia coli* TOP10	Harbouring the pBluescript II SK	Invitrogen®
*Streptomyces coelicolor* M145	SCP1-SCP2-harbouring empty plasmid pNG3	Kieser (2000)
*Streptomyces coelicolor* M145	pGWS1574 ( habouring *ftsZ* under its own promoter on pNG3)	This study
*E. coli ET12567/pUZ8002*	*E. coli* ET12567 containing plasmid pUZ8002, a not self-transmissible plasmid which can mobilize other plasmids; Cln^R^, Km^R^	Flett et al. (1997), MacNeil et al. (1992)
*Plasmids*		
pNG3	Cloning vector; Hyg^R^, Amp^R^	Gonzalez-Quinonez et al. (2016)
pBluescript II SK	Cloning vector; Amp^R^	Agilent®
pCR™-Blunt II-TOPO®	Zero Blunt®TOPO®PCR Cloning Kit, Km^R^	Invitrogen®
pHJL401	Cloning vector; Tsr^R^	Larson and Hershberger (1986)
pCRISPR-Cas9	Conjugative and thermosensitive plasmid harbouring Cas9	Tong et al. (2015)
pCRISPR-SgFtsZ	pCRISPR-Cas9 harbouring the target SCO2082 sequence and a 2 kb fragment surrounding the ftsZ ORF used to knockdown ftsZ	This study
FtsZ*-EE pNG3	pNG3 harboring ftsZ* pGlu319 and pGlu387; Hygr^R^, Amp^R^, Hyg^R^	Rioseras et al. (2018)
FtsZ*-AA pNG3	pNG3 harboring ftsZ* pGlu319 and Ala387; Hygr^R^, Amp^R^, Hyg^R^	Rioseras et al. (2018)
FtsZ*-EA pNG3	pNG3 harboring ftsZ* Ala387 and pGlu387; Hyg^R^, Amp^R^, Hyg^R^	This study
FtsZ*-AE pNG3	pNG3 harboring ftsZ* Ala319 and Ala387; Hyg^R^, Amp^R^, Hyg^R^	This study
FtsZ*-EE eGFP pHJL401	pHJL401 harbouring ftsZ*EE-egfp	This study
FtsZ*-AA eGFP pHJL401	pHJL401 harbouring ftsZ*AA-egfp	This study
FtsZ*-EA eGFP pHJL401	pHJL401 harbouring ftsZ*EA-egfp	This study
FtsZ*-AE eGFP pHJL401	pHJL401 harbouring ftsZ*AE-egfp	This study
*Primers*		
SCO4848F	CGTCGTATCCCCTCGGTTG	Gonzalez-Quinonez et al. (2016)
pMS82R	GAGCCGGGAAAGCTCATTCA	Gonzalez-Quinonez et al. (2016)
FtsZ_F	GGACTAGTAGCAGGGTGTGCGGAAG	This study
FtsZ_R	AAGATATCCTATCACTTCAGGAAGTCCG	This study
sgRNA-F (NcoI)	CATGCCATGGCGATGACTTTGATGACTGCGGTTTTAGAGCTAGAAATAGC	This study
sgRNA-R (SnaBI)	ACGCCTACGTAAAAAAAGCACCGACTCGGTGCC	This study
2082LeftF	AGGCCTAGACCGACCACCGCCGAG	This study
2082LeftR	CCTATCACTTCAGGAAGTCCGTGATGACTGCGAGGTAGTTCTG	This study
2082RightF	CAGAACTACCTCGCAGTCATCACGGACTTCCTGAAGTGATAGG	This study
2082RightR	AGGCCTAGTAACCGACCACGGAACGCA	This study
eGFP-FTSZ* F	GTCAGAATTCAGGCCTTCGACGTGGCAGCACCGCAGAACTACC	This study
eGFP-FTSZ* R	GTCAAAGCTTGGATCCTTCAGGAAGTCCGGCACGTCC	This Study

### Cell viability and morphology observations at the confocal microscope

For the analysis of hypha viability, morphology and sporulation timing, cultures were grown on GYM, harvested at different time points from cellophane-grown mycelia, stained with the LIVE/DEAD BacLight Bacterial Viability Kit (Invitrogen, L-13152) and observed under the confocal microscope following our previous protocol (Manteca et al. 2005). The LIVE/DEAD BacLight Bacterial Viability Kit consists in dry SYTO 9 and Propidium Iodide (PI) which we prepared at the concentrations recommended by Invitrogen, 6 µM and 30 µM respectively (both were prepared in ultrapure mQ water). Cellophane squares (1 cm side) were manually cut, placed over the microscope slide, 20 µL of the SYTO9/PI mixture was added, a cover glass was carefully putted over the sample preventing bubble formation, the sample was incubated at room temperature for 5 min, and immediately observed at the microscope. This kit uses SYTO9 and Propidium Iodide (PI), two DNA-binding colorants. SYTO9 penetrates intact membranes and stains viable cells green, whereas PI (staining red) only penetrates bacteria with damaged membranes. PI displaces SYTO9 from DNA when both colorants are present in dying cells. Samples were observed using an inverted Zeiss Axio Observer Z laser scanning microscope at wavelengths of 488 and 568 nm for excitation and 530 (green) or 630 nm (red) for emission.

For the analysis of septa formation, square microscopy cover glasses were positioned in SFM plates under an angle of 45°, and subsequently, 10 μl of a spore suspension were inoculated (10^8^ spores/ml). After 3–5 days (depending on the strain) of incubation at 30 °C, the cover glass was removed and 5 µg/ml of WGA-Alexa fluor 633 were added for cell-wall (peptidoglycan) staining. Samples were observed using an inverted Leica SP8 laser scanning microscope at wavelengths of 632 nm for excitation and 647 nm for emission.

Unstained samples (processed with ultrapure mQ water instead SYTO9/PI or WGA-Alexa fluor) were used as controls to fix the PMT gain levels at which autofluorescence was detected, which were much higher than the gain used to collect pictures. Autofluorescence, was extremely low, compared to the stained samples. At least three biological replicates were processed for each sample.

### Scanning electron microscopy (SEM)

Scanning electron microscopy (SEM) was carried out as described (Colson et al. 2008). For this, small blocks of *Streptomyces* cultures grown on SFM medium were fixed using glutaraldehyde, dehydrated and dried. Eventually, the samples in 100% acetone were completely dried in a critical point dryer. Cells were mounted on an SEM stub and sputter-coated with platinum palladium to capture the images at 15 kV. SEM pictures were used for measuring spore length. The lengths of more than 250 spores were measured in each strain using the ROI manager plugin of ImageJ. Statistical significance was measured by comparing spore lengths in wild-type and the four mutants using a T-test. Three biological replicates; 3 plates/mutant, and three methodological replicates; 3 blocks per plate were used for SEM experiments.

### Transmission electron microscopy (TEM)

For visualizing the spore chains of *Streptomyces*, small blocks were cut from SFM plates harvested in confluent cultures and processed for TEM essentially as described (Piette et al. 2005). The mycelium was washed with 1 × PBS before fixation with 1.5% glutaraldehyde, and blocks were then post-fixed with 1% osmium tetroxide for 30 min. The samples were dehydrated by passing them through an ethanol gradient and placed in propylene oxide for 15 min followed by incubation in a mixture of Epon and propylene oxide (1:1) and pure Epon (each step 45 min). Finally, the samples were embedded in Epon and sectioned into 70 nm slices, which were placed on 200-mesh copper grids. Samples were stained using uranyl-430 acetate (2%) and lead citrate (0.4%), if necessary, and imaged at 70 kV in a Jeol 1010 transmission electron microscope.

Three biological replicates; 3 plates/mutant, and three methodological replicates; 3 blocks per plate were used for SEM experiments.

### Stereo microscopy

Single colonies of *S. coelicolor* and the mutant strains were observed using a Leica M80 stereomicroscope. Pictures were taken with a Leica DFC295 camera.

### Antibiotic measurements

Undecylprodigiosin and actinorhodin were quantified spectrophotometrically according to Tsao et al. (1985) and Bystrykh et al. (1996). Cells were ruptured in the culture medium by adding 0.1 N KOH. After vortexing and centrifugation, actinorhodin was quantified in the supernatant (ɛ_640_ = 25,320). Undecylprodigiosin was measured after vacuum drying of the mycelium, followed by extraction with methanol, acidification with HCl (to 0.5 M), and a spectrophotometric assay (ɛ_530_ = 100,500). Reproducibility has been corroborated by at least three independent cultures.

### Resistance of spores to lysozyme, heating, and freezing

For heating (55 °C, 30 min) and freezing (− 20 °C, 24 h) shock treatments, suspensions of 10^6^ spores/ml were prepared in sterile distilled water and subjected to different treatments as detailed below. Germination of the spores before and after treatment was analysed by plating several dilutions and quantifying the number of colony-forming units (Rioseras et al. 2016). All quantifications were measured in triplicate. The data correspond to the average ± SD of the replicates. For lysozyme resistance, 10^6^ spores/ml were plated in LB agar medium and directly placing filter discs containing 50 μg, following the method of Kleinschnitz et al. (2011), and incubated at 30 °C for 3 days.

### *ftsZ*-eGFP alleles creation and observation at the confocal and fluorescence microscopes

The pHJL401 plasmid (Larson and Hershberger 1986) (Table 1), an E*. coli/Streptomyces* shuttle vector, was used for the constructions of the four different ftsZ* (Serine modifications) and the *egfp* expression under the control of the *ftsZ* promoter. The *ftsZ* promoter is cloned between sites *EcoR*I-*Stu*I, the ftsZ* alleles without stop codon are cloned *Stu*I-*BamH*I and the *egfp* is cloned downstream *BamH*I-*Not*I.

The strains with the eGFP fusion were analyzed by Axio Observer Zeiss confocal microscope. Excitation was performed with a 488 nm laser, and detection was performed with a 505–530 nm bandpass filter. The first 16 h of growth, were observed making time-lapse experiments as follows; cultures were pre-grown on GYM medium for 6 h at 30 °C for the germination of the spores, samples were then excised out and inverted into µ-dishes (Ibidi GmbH 35 mm, hight ibitreat). Pictures were taken every 10 min during 16 h following Yagüe et al. (2016). Wild-type cultures (without FtsZ-eGFP fusions) were used as controls to fix the PMT gain levels at which autofluorescence was detected, which were much higher than the gain used to collect the pictures. For analysing the FtsZ-eGFP at later time points, cover glasses were positioned in SFM plates under an angle of 45°, and subsequently, 10 μl of a spore suspension were inoculated (10^8^ spores/ml). At the indicated time-points, cover glasses were removed, mounted with ultrapure mQ water, and observed using a Leica DMRXA fluorescence microscope with a FITC filter. Pictures were taken with an ORCA-Flash4.0 V3 Digital CMOS camera. Wild-type strain cultures (without FtsZ-eGFP fusions) were used controls to fix the levels at which autofluorescence was detected. At least three biological replicates were processed for each sample.

### Phase contrast images microscopy

For analysing the sporulation of FtsZ*strains, cover glasses were positioned in SFM plates under an angle of 45°, and subsequently, 10 μl of a spore suspension were inoculated (10^8^ spores/ml). After 5–7 days, depending on the strain, cover glasses were removed, mounted with ultrapure mQ water, and observed using phase contrast under the Leica DMRXA microscope. Pictures were taken with an ORCA-Flash4.0 V3 Digital CMOS camera.

### Image processing

Microscopy images were processed (histogram intensity levels were adjusted and scale added) using the Fiji software (Schindelin et al. 2012). Figure composites were made using AdobePhotosop CS5.1 and Adobe Photoshop 2021.

## Results

### Construction of *Streptomyces coelicolor* strains harbouring ftsZ* alleles mimicking FtsZ phosphorylation/non-phosphorylation

*Streptomyces coelicolor* FtsZ (SCO2082) is differentially phosphorylated at Ser319 and Ser387 during the cell cycle (Rioseras et al. 2018; Manteca et al. 2011). These serine residues are located in the protein linker, just between the global domain and the C-terminal part of FtsZ (Fig. 1A). With the aim of analysing the effect of FtsZ serine-phosphorylation in *S. coelicolor*, we used a well-established methodology based on the substitution of the Ser residues by Glu or Ala, thus mimicking phosphorylation or non-phosphorylation, respectively (Zhao et al. 1994; Keller-Pinter et al. 2017; Hewitt et al. 2017; Morrison et al. 2003; Trutnyeva et al. 2005). Four different mutant variants were studied: two mutants previously created, namely FtsZ-EE (EE; mimicking two phosphorylations) and FtsZ-AA (AA; mimicking non-phosphorylation) (Rioseras et al. 2018); and two new mutants mimicking phosphorylation in only one of the serines, namely FtsZ-EA Glu-Ala (EA; mimicking phosphorylation at Ser319 and no phosphorylation at Ser387), and FtsZ-AE Ala-Glu (AE; mimicking no phosphorylation at Ser319 and phosphorylation at Ser387) (Fig. 1B). The strains, each expressing one of the FtsZ variants, were generated by the introduction of the mutant copies into an *ftsZ* null background. For this, the recombinant ftsZ*—transcribed from the native *ftsZ* promoter region (Flardh et al. 2000)—was introduced into integrative vector pNG3 (Gonzalez-Quinonez et al. 2016) and introduced into *S. coelicolor* M145. The native *ftsZ* was subsequently inactivated using Crispr-Cas9. As detailed below, each mutant showed a distinctive phenotype in antibiotic production, single colony morphology and sporulation (sporulation timing and spore resistance) indicating that the different FtsZ phosphomimetic alleles led to different phenotypes.

### Phosphorylation affects FtsZ Z-ladder formation during sporulation.

**Fig. 2:**
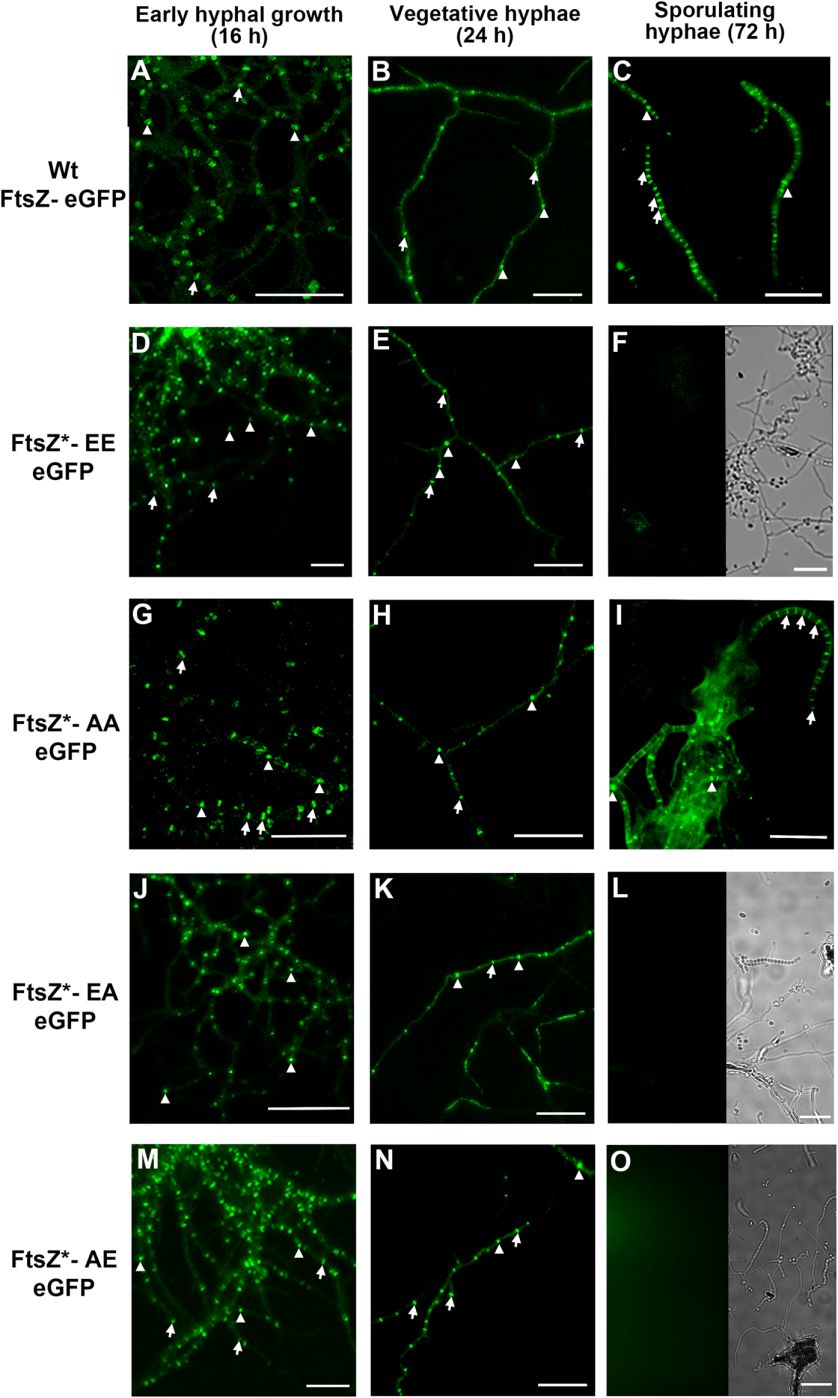
Z-rings and ladders formation in the ftsZ*-eGFP alleles. Fluorescence micrographs of the young compartmentalised hyphae (growing on GYM), substrate and aerial mycelium (growing on SFM) and sporulating hyphae (growing on SFM) are shown. Contrast mode images are shown for the FtsZ-EE, FtsZ-EA, and FtsZ-AE mutants to illustrate that they are sporulating. Only FtsZ-AA form sporulation Z-ladders. Representative images from at least three biological replicates are shown. Arrows indicate Z-rings. Arroheads indicate FtsZ-eGFP accumulations that do not cross the transverse axis of the hyphae and do not constitute mature Z-rings. Scale bars 10 µm

To study Z-ring and Z-ladder formation (1 µm spaced Z-rings), we followed the methodology developed by Grantcharova et al. (2005), where ftsZ-eGFP is ectopically expressed in a *S. coelicolor* strain carrying the native *ftsZ* gene. This approach was demonstrated to be useful to form functional FtsZ-eGFP Z-rings that can be observed during development and was largely replicated in *Streptomyces* [see for instance Willemse and van Wezel (2009), Yague et al. (2016), Bush et al. (2022)].We expressed FtsZ-eGFP fusions from the native *ftsZ* promoter region as previously described (Grantcharova et al. 2005). This was done for all four mutants (expressing FtsZ-AA, AE, EA and EE) and for wild-type *fstZ*. The constructs were introduced into the *S. coelicolor* M145 and Z-ring formation was analysed using fluorescence microscopy (Fig. 2). During *Streptomyces* development, there are two stages showing massive Z-ring formation. The first one during the early development (the first 16 h culture) corresponding to the early compartmentalised mycelium suffering a massive, but non-synchronic, cell division (named as first mycelium or MI) (Yague et al. 2016) and the second one corresponding to the sporulating hyphae suffering a massive synchronic cell division leading to the formation of Z-ladders (i.e. 1 µm spaced Z-rings) (Grantcharova et al. 2005). Both stages are separated by the substrate and aerial mycelium hyphae which are multinucleated having sporadic septation and few Z-rings (Grantcharova et al. 2005; Yague et al. 2016). We used GYM (a medium in which development is slow, facilitating vegetative growth analysis) to study vegetative Z-rings and SFM (one of the best medium to obtain fast and abundant sporulation) to study sporulation Z-ladders (Fig. 2). Z-ring formation was unaffected in all FtsZ variants during vegetative growth: FtsZ-eGFP rings (arrows in Fig. 2) and FtsZ-eGFP accumulations that do not cross the transverse axis of the hyphae and do not constitute mature Z-rings (arrowheads in Fig. 2) are present in the young compartmentalised hyphae (16 h) as well as in the substrate mycelium. Interestingly, the FtsZ-eGFP ladders associated with sporulation (Fig. 2C) were absent in the strains expressing the FtsZ-EE, FtsZ-EA, and FtsZ-AE FtsZ alleles (Fig. 2F, L, O). It is likely that sporulation ladders are formed also in these mutants, but they likely consist of wild-type FtsZ only, or with very low amounts of the phosphomimetic FtsZ-eGFP alleles that preclude their visualisation. By contrast, the FtsZ-AA mutant mimicking the non-phosphorylated version of FtsZ, showed normal Z-ladders (Fig. 2I).

### FtsZ phosphorylation affects antibiotic production and colony morphology

**Fig. 3:**
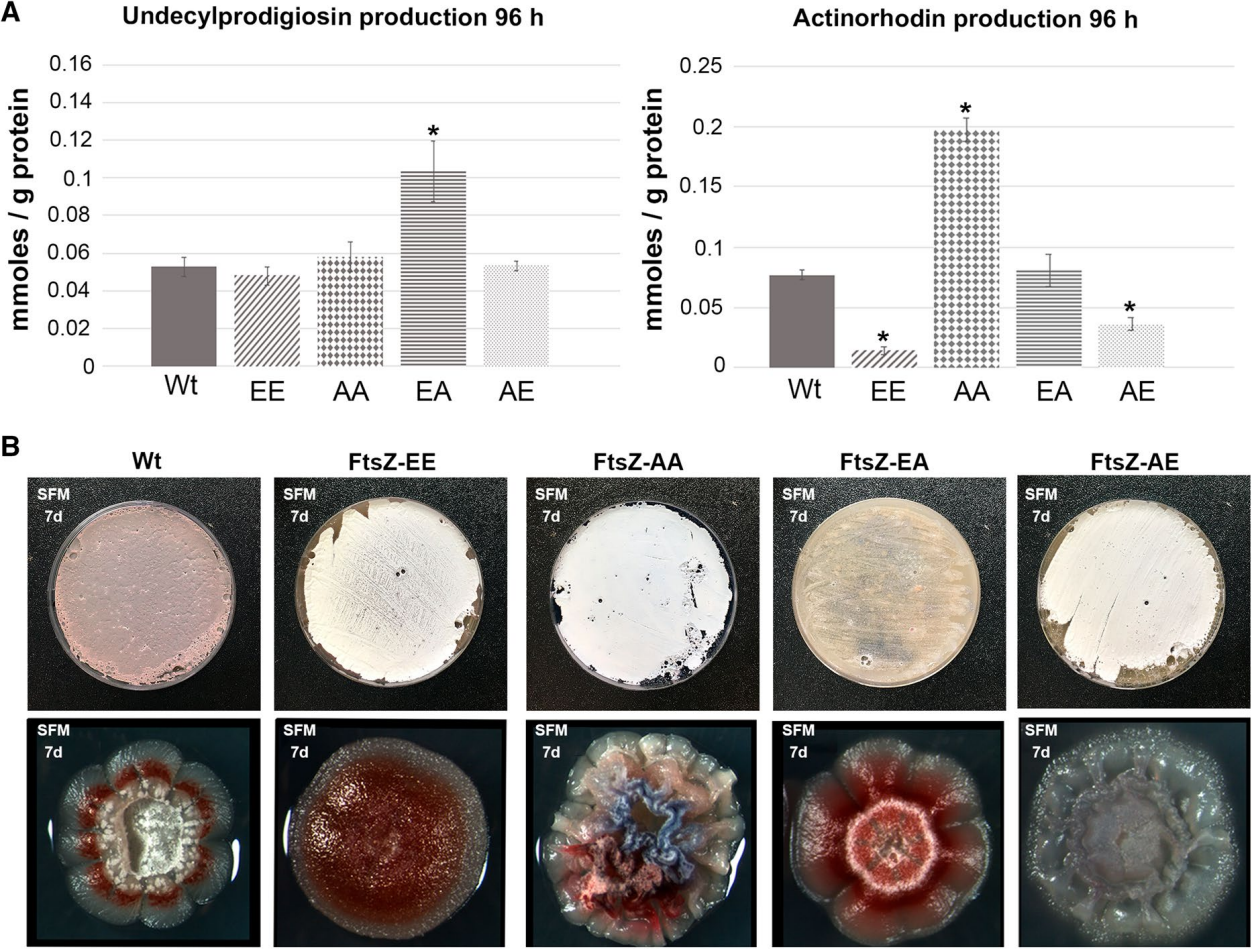
Antibiotic production in liquid cultures and growth on solid medium. **A** Actinorhodin and undecylprodigiosin productions. FtsZ-EA overproduces Red, FtsZ-AA overproduces Act while FtsZ-EE and FtsZ-AE have this production reduced. Three biological replicates were processed. Asterisks in the graphs mark significant differences (t-test, *p* value < 0.05). **B** Macroscopic view in SFM lawn growth and single colonies. All mutants show reduced and delay in sporulation and different colony morphologies. Representative images from at least three biological replicates are shown. More colonies of the FtsZ mutants are shown in Supplementary Fig. 1B

We previously reported altered antibiotic production in the double mutants (FtsZ AA and EE) (Rioseras et al. 2018). To study this phenomenon in more detail and also for the single mutants, we analysed undecylprodigiosin (Red, red-pigmented) and actinorhodin (Act, blue-pigmented) production in liquid cultures of the wild-type and the four mutants (Fig. 3A). FtsZ-EA produced more Red than all other strains (Fig. 3A). FtsZ-EE and FtsZ-AE showed strongly reduced Act and FtsZ-AA overproduced Act (Fig. 3A).

The macroscopic appearance was then analysed on solid SFM medium (Fig. 3B). Freshly harvested spores in same concentrations were plated on SFM agar plates. After 7 days, confluent sporulating cultures revealed differences between the mutants and their parental strain. The FtsZ-EE, FtsZ-AA, and FtsZ-AE mutants showed a white phenotype, indicative of aerial hyphae formation but a failure to produce grey-pigmented spores at this time point (Fig. 3B). Mutant FtsZ-EA failed to produce aerial hyphae, showing a phenotype comparable to that of *Streptomyces* bald mutants (Merrick 1976) (Fig. 3B). However, the FtsZ-EA mutant is not bald, as it is able to sporulate after prolonged incubation (10 days).

At the single colony level, morphological differences between the colonies were seen between the mutants and the parent *S. coelicolor* M145 (Fig. 3B); the wild-type strain colonies had colourless edges (no antibiotic production), a ring of red pigment (reflecting undecylprodigiosin production), and some aerial mycelium at the top of the colony. FtsZ-EE colonies failed to develop aerial hyphae, producing undecylprodigiosin; FtsZ-AA colonies are highly irregular and wrinkled, producing both Red and Act and developed aerial hyphae at the top; FtsZ-EA colonies produced significantly fewer aerial hypha and spores than the wild-type strain; FtsZ-AE single colonies show irregular edges and light blue pigmentation and produced aerial mycelium in the centre (Fig. 3B; see pictures illustrating more wild-type and FtsZ-EE-AA-EA-AE mutant colonies in supplementary Fig. 1B).

### FtsZ phosphorylation affects spore length, morphology, and resistance

**Fig. 4:**
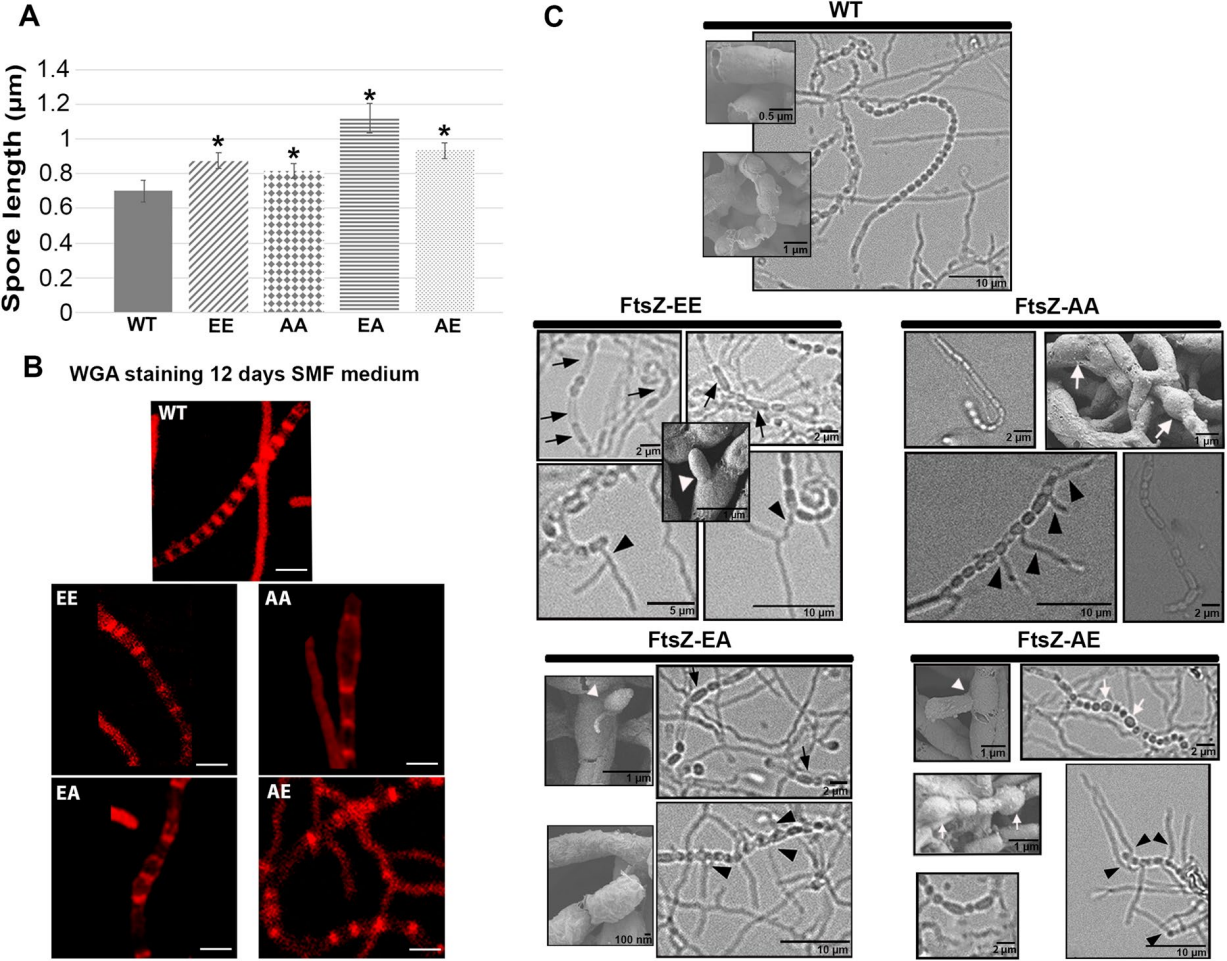
Aberrant sporulation. **A** Spore length measurements. All mutants show larger spores than the wild-type. Asterisks in the graphs mark significant differences (t-test). **B** WGA staining in SFM cultures. Scale bar 2 µm. **C** Contrast mode images and SEM micrographs of spore chains (SFM cultures). Longer/immature spores (black arrows); swollen spores (white arrows) spores germinating in the spore chain (arrowheads)

Sporulation was analysed in detail, using plates with same quantity of spores in every strain (10^5^ espores/plate) by phase-contrast microscopy, fluorescence microscopy and cryo-scanning electron microscopy (Fig. 4). Spore length was thereby quantified using scanning electron micrographs (Fig. 4A and supplementary Fig. 2). The average spore-length of the mutants was significantly longer than those of the wild-type strain (Fig. 4A). In line with the fact that spores in mutants are longer, WGA-Alexa fluor 633 staining to detect de novo septum synthesis during sporulation, revealed that the spacing between the septa in aerial hyphae was larger in the mutants than in the wild-type strain (Fig. 4B), which correlates well with an increase in the spore diameters in the mutants. Precocious germination was observed in the spore chains of all the mutants (black arrowheads in phase-contrast and white arrowheads in SEM micrographs in Fig. 4C). Such ectopic germination was seen previously in *dasA* and *sepF* mutants (Zhang et al. 2020a; Colson et al. 2008). SEM images also revealed that FtsZ-EA mutant spores have an aberrant wrinkled spore coat (Fig. 4C). FtsZ-AA and FtsZ-AE show swollen spores not observed in the wild-type strain (compare FtsZ-AA and FtsZ-AE SEM images with the wild-type SEM images in Fig. 4C and Supplementary Fig. 3A).

**Fig. 5:**
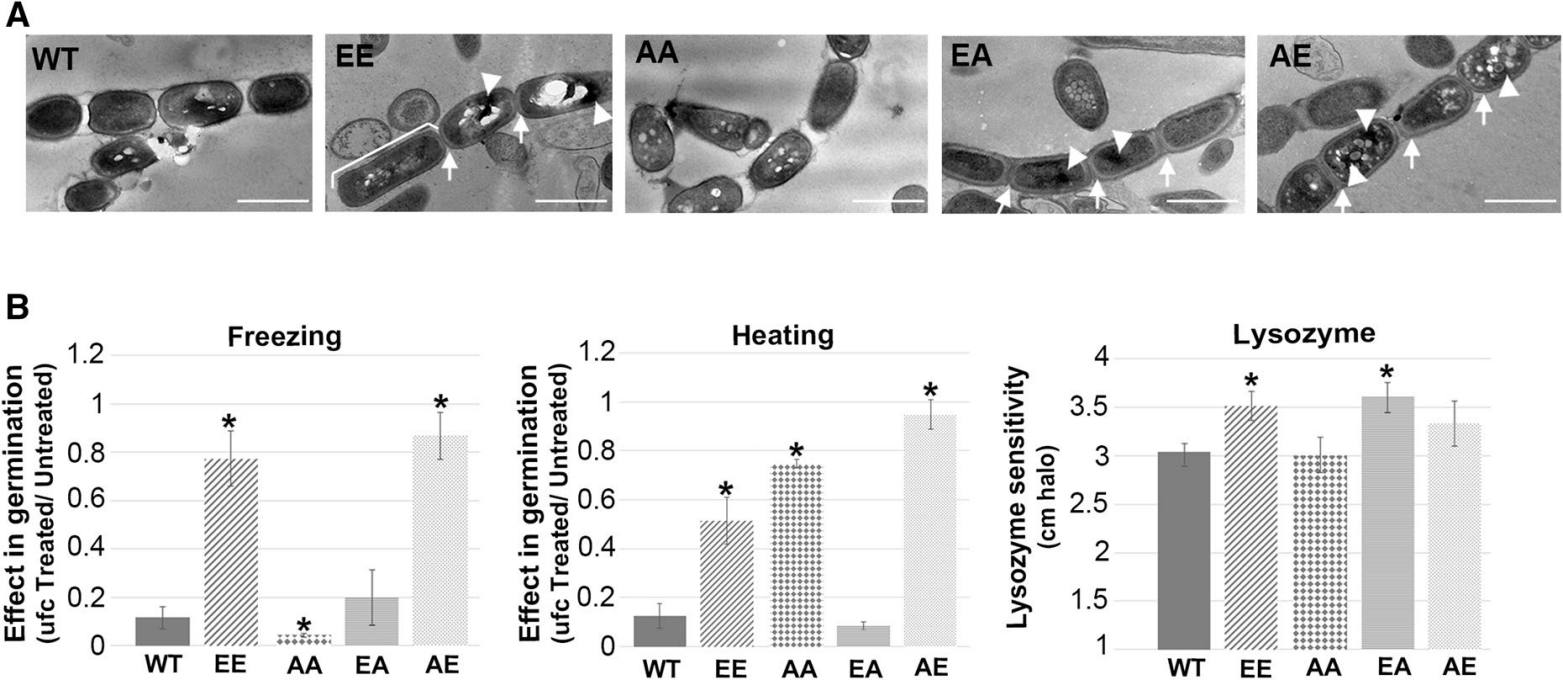
Spore chains and shock treatments. **A** Transmission electron micrographs of spore chains (SFM cultures). Scale bar 1 µm. Thick spore envelope (arrows); immature spores (bracket); irregular nucleoid compaction (asterisks). **B** Spore stress resistance. Asterisks in the graphs mark significant differences (t-test, *p* value < 0.05)

Spores were further analysed by transmission electron microscopy (TEM) (Fig. 5). The FtsZ-EE, AE and EA spore envelopes were thicker than those of the parent (arrows in Fig. 5A and Supplementary Fig. 3B). Many of the FtsZ-EE mutant spore chains contained rectangular spores (bracket in Fig. 5A, also visible Fig. 4C, arrows, and Supplementary Figs. 3C, 4C) that might be immature (McVittie 1974; Keijser et al. 2003). Nucleoids in FtsZ-EE, AE and EA could be not condensed as well as in the parental strain [arrowheads pointing black spots in Fig. 5A, see reference Zhang et al. (2016)].

Spore resistance to lysozyme, heating, and freezing was tested (Fig. 5B). Fresh spores were harvested from SFM-grown cultures of all strains and diluted to a concentration of 10^6^ spores/ml (based on viable counts). Non-treated spores were used as a control. After treatment, serial dilutions were plated to quantify viability as colony-forming units per ml (cfu/ml). We tested the resistance of the spores to freezing (24 h at − 20 °C), heat shock (30'at 55 °C), and lysozyme (50 μg). FtsZ-EE and FtsZ-AE spores were much more resistant to freezing than wild-type spores, while FtsZ-AA and FtsZ-EA spores showed comparable resistance as those of the parent (Fig. 5B). In terms of heat stress, FtsZ-EE, AA and AE spores were much more resistant than those of the parent, while FtsZ-AA spores have similar heat resistance as wild-type spores. Finally, small but statistically significant changes in lysozyme sensitivity were observed, with FtsZ-EE, EA, and AE, spores slightly more sensitivity than the *S. coelicolor* M145 wild-type strain, while FtsZ-AA spores had similar sensitivity as those of the parental strain (Fig. 5B).

### Substrate and aerial hypha morphology is affected by the FtsZ phosphorylation

**Fig. 6:**
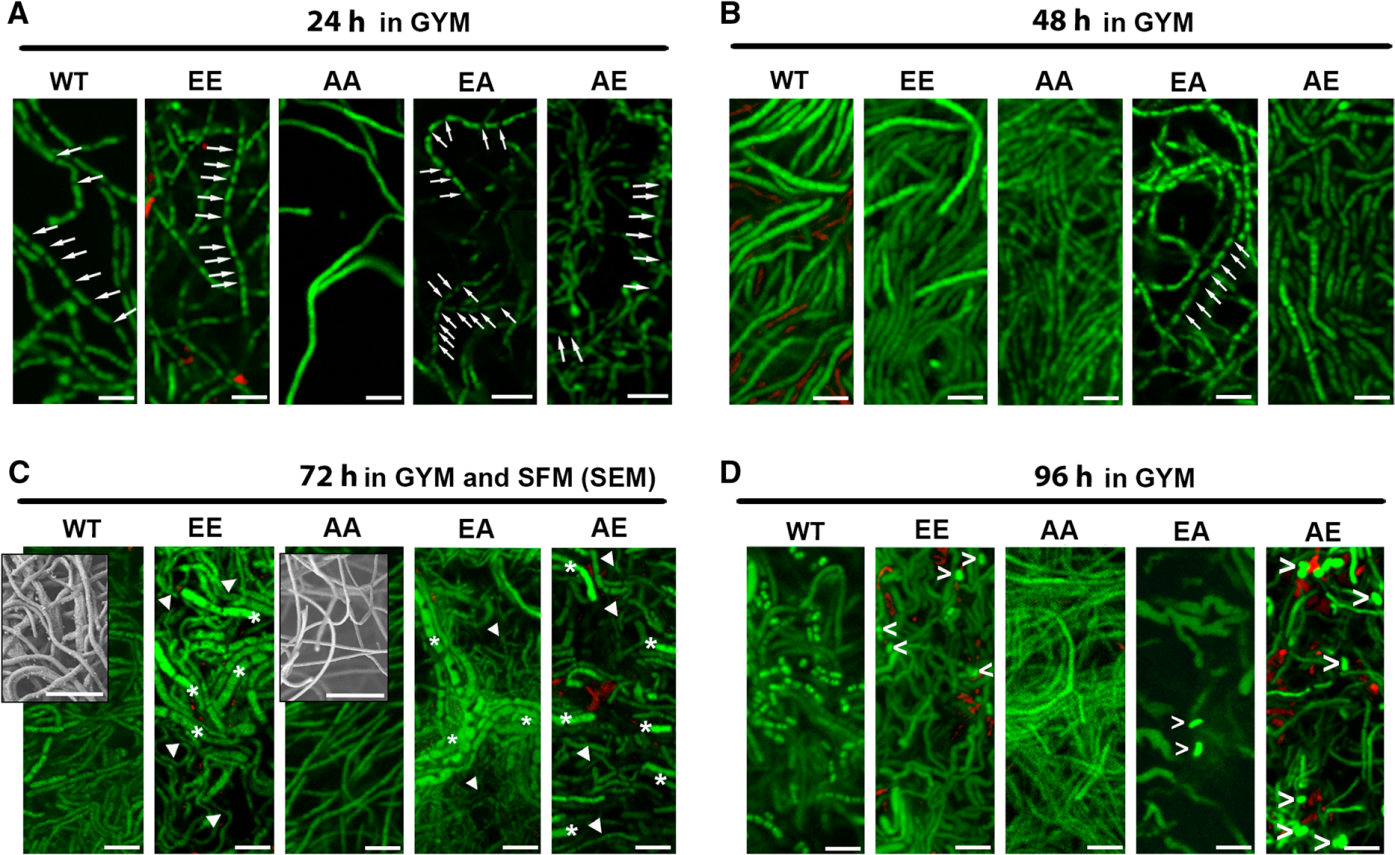
Confocal micrographs of GYM solid cultures stained with SYTO 9 and PI showing that young compartmentalised hyphae, substrate mycelium and sporulation timings are affected in the FtsZ mutants. Dead cellular segments of the young compartmentalised hyphae appear as discontinuities when stained with SYTO 9/PI because its DNA is degraded (Yague et al. 2016) (arrows); substrate and aerial mycelium hyphae do not show discontinuities when they are stained with SYTO 9 and PI; reduced sporulation in the FtsZ-EE, -EA and A-E mutants ins labelled by triangles ( >). Representative images from at least three biological replicates are shown. 6C shows electron micrographs illustrating thin hypha in Ftsz-AA compared to the wild type. Scale bar 5 µm

As defective sporulation and changes in secondary metabolism were observed, we analysed if the substrate mycelium was also affected in the FtsZ phosphorylation mutants. For that purpose, the wild-type and EE, EA, AE, AA mutants were plated as confluent lawns on GYM agar, a culture medium in which the development is much slower than in SFM, allowing a more precise separation of the developmental stages (Manteca et al. 2008). Hyphae were stained with SYTO9 and PI and observed under the laser scanning confocal microscope. In *Streptomyces* development, there are three kind of differentiated hyphae: during the early development (the first 16-h culture) the compartmentalised mycelium suffers a programmed cell death in which live and dead cells alternate in the same hyphae, at the end of this programmed cell death, dead cellular segments appear as discontinuities in the hyphae when stained with SYTO 9/PI because its DNA is degraded (arrows in Fig. 6) (Manteca et al. 2006; Yague et al. 2016); living segments differentiates to the substrate multinucleated hyphae that do not show discontinuities when they are stained with SYTO 9 and PI (Yague et al. 2016); substrate hyphae differentiate to aerial mycelium which suffers a synchronic and massive cell division that generate spores chains (Claessen et al. 2006).

At 24 h on GYM cultures the wild-type strain and the EE, EA, AE mutants show the discontinuities characterising the early young compartmentalised hyphae (Yague et al. 2016) (arrows in Fig. 6A), while the AA mutant does not, indicating an accelerated development of the multinucleated substrate mycelium in this last mutant (Manteca et al. 2008). In 48 h on GYM cultures, the EA mutant showed a delay in development, with the discontinuities of the young compartmentalised mycelium still visible (Manteca et al. 2008) (arrows in Fig. 6B). After 72 h, the EE, AA, EA, AE mutants showed a mix of thin (arrowheads Fig. 6C and supplementary Fig. 4) and thick hyphae (asterisks in Fig. 6C and supplementary Fig. 4), that were not visible in the wild-type strain (Fig. 6C). At this time point, AA hyphae were surprisingly thin, with a hyphal diameter of only 0.3–0.5 µm showed in both, GYM and SFM (Fig. 6C) cultures (SEM zooms in Fig. 6C and supplementary Fig. 3B). After 96 h, the mutants hardly sporulated (exceptions highlighted with triangles in Fig. 6D), while the parental strain sporulated very well (Fig. 6D).

## Discussion

The tubulin homologue FtsZ forms the cell division scaffold in bacteria and plays a key role in the subsequent recruitment of the divisome components. FtsZ plays a key role in the recruitment of the divisome components. In streptomycetes, canonical cell division, i.e. leading to the cytokinesis into two daughter cells, takes place only during sporulation. By contrast, during vegetative growth, FtsZ is required for the formation of occasional cross-walls, but this process is independent of most divisome proteins. An important question about *Streptomyces* cell division is what sets these two processes apart, and what the role is of FtsZ. The N-terminal GTPase domain of *Streptomyces* FtsZ plays a major role in the septation of *Streptomyces* spore chains (Sen et al. 2019). In rod-shaped bacteria, the FtsZ C-terminal part is known to be a hotspot for protein interactions, such as with FtsA, SepF, ZipA, ZapA, ZapD and others (Hale and de Boer 1997; Pichoff and Lutkenhaus 2002; Low et al. 2004; Hamoen et al. 2006; Singh et al. 2008; Durand-Heredia et al. 2012; Krol et al. 2012). These interactors play an important role in the assembly, localization, polymerization, and membrane tethering of the Z-ring. Of these proteins, only SepF is found in streptomycetes.

Shot-gun phosphoproteomic studies showed that FtsZ (SCO2082) is phosphorylated at two serine residues, Ser319 and Ser387 (Rioseras et al. 2018; Manteca et al. 2011; Hirakata et al. 2019). These two phosphorylation sites are located in the protein linker close to the C-terminal FtsZ domain (Fig. 1). The important role of the conserved C-terminal end of FtsZ in protein–protein interactions suggests that the phosphorylation of the two serine residues identified might play an important role in *Streptomyces* Z-ring dynamics*.* Our study describes the analysis of mutants expressing variants of FtsZ wherein one or both Ser residues were mutated to either Ala (mimicking the non-phosphorylated state) or Glu (mimicking the phosphorylated state), or a combination thereof (FtsZ-AA, AE, EA or EE). We thereby looked at the effect of the mutants on morphogenesis and antibiotic production of *S. coelicolor*, in a background where the parental *ftsZ* had been deleted. In addition, the localization of FtsZ-eGFP fusions was analysed, for mutants and wild-type protein, in this case, and following the workflow designed by Grantcharova et al. (2005), in the presence of a wild-type copy of *ftsZ*, to ensure that sporulation was normal. The fluorescent hybrid FtsZ*-eGFP proteins expressing variants EE, EA, and AE did not form fluorescent Z-ladders (Fig. 2). This strongly suggests that these mutant FtsZ isoforms have a much lower propensity to form sporulation Z-ladders than the native FtsZ-eGFP, and/or are competed out by wild-type FtsZ. Conversely, the AA allele (which mimics the fully non-phosphorylated state) formed apparently normal FtsZ-eGFP-ladders (Fig. 2). These results suggest that the phosphorylation status determines the ability of FtsZ to form Z-ladders during sporulation-specific cell division, either directly or indirectly due the change in its interaction with partners that support its polymerization, and hence, is a major determinant for sporulation. Further work will be necessary to determine the specific level of FtsZ phosphorylation necessary to allow normal sporulation.

The FtsZ phosphorylation could precipitate pleiotropic effects due to the stress triggered by abnormal growth and development affecting antibiotic production (Fig. 3A), single colony morphology (Fig. 3B) and sporulation (sporulation timing and spore resistance) (Figs. 3B, 4 and 5C). Cell division and hypha compartmentalisation were described to be central in *Streptomyces* development, affecting secondary metabolism which is activated at the substrate and aerial mycelium hyphae (Manteca et al. 2008) and it is dramatically enhanced in cells with significant chromosomal changes that are not able to sporulate (Zhang et al. 2020b). Each mutant showed a distinctive phenotype in antibiotic production, single colony morphology and sporulation (sporulation timing and spore resistance to physicochemical stress) indicating that the different FtsZ phosphomimetic alleles led to different phenotypes. The exception is the precocious germination observed in spore chains, which was observed in all the ftsZ mutants (arrowheads in Fig. 4C), and we cannot rule out that it is a consequence of the expression of the *ftsZ* alleles outside its natural position in the chromosome, instead of an effect of the alteration of Ser phosphorylation.

The role of Ser/Thr/Tyr phosphorylation in the regulation of cell shape and sporulation has been reported for *Mycobacterium* (Molle and Kremer 2010; Kang et al. 2005; Thakur and Chakraborti 2006; Prisic et al. 2010) and *Streptomyces* (Vollmer et al. 2019; Ladwig et al. 2015). The PknA and PknB Ser/Thr/Tyr kinases strongly influence *Mycobacterium* cell shape, with either overexpression or depletion resulting in major alterations in cell morphology (Kang et al. 2005). Such phenotypes are not uncommon and are found in various other bacteria that are affected in the expression of Ser/Thr/Tyr kinases (Kang et al. 2005). In *Streptomyces*, the Ser/Thr/Tyr kinases PkaI and PkaH regulate the activity of the so-called *Streptomyces* spore-wall synthesizing complex (SSSC), modulating spore size (Vollmer et al. 2019; Ladwig et al. 2015). The activity of DivIVA, which mediates polar growth and hyphae branching elongation, is modulated by the Ser/Thr kinase AfsK (Willemse et al. 2011). An S*. coelicolor pkaE* null mutant was affected in antibiotic production and sporulation, and FtsZ was recognised as one of the proteins phosphorylated by PkaE (Hirakata et al. 2019). We here describe that FtsZ phosphorylation affects FtsZ polymerisation and bacterial cell shape.

Ser319 and Ser387 are highly conserved in most of the *Streptomyces* FtsZ sequences deposited in the GeneBank database. However, some species have Ala instead Ser319 (we found Ala in *S. venezuelae* and *S. griseus*) and some species have Thr instead Ser387 (we found Thr in *S. griseus*). Interestingly, the amino acids surrounding Ser319 and Ser387 were conserved in all the *Streptomyces* FtsZ sequences that appeared in our GeneBank BLAST searches. These data indicate the importance of the Ser319 and Ser387 surrounding regions and that both Ser positions can be modified (at least to Ala and Thr) reporting viable phenotypes. Alternative phosphorylation site/s in the FtsZ orthologues lacking Ser319 or Ser387 might be possible.

Taken together, our results indicate that FtsZ serine phosphorylation modulates the dynamics of FtsZ polymerisation during sporulation, thereby pleiotropically affecting development and antibiotic production. Cell division is central in the development and the FtsZ phosphorylation could precipitate pleiotropic effects due to the stress triggered by abnormal growth and development. Further work will be necessary to study whether the pleiotropic effect of FtsZ Ser/Thr/Tyr phosphorylation observed in *Streptomyces* might be general in bacteria.

## Supplementary Information

Below is the link to the electronic supplementary material.

Supplementary file1 (PDF 6291 KB)

Supplementary file2 (PDF 4605 KB)

Supplementary file3 (PDF 1624 KB)

Supplementary file4 (PDF 653 KB)
