# Supplementary material for: FtsZ phosphorylation pleiotropically affects Z-ladder formation, antibiotic production, and morphogenesis in Streptomyces coelicolor
Source: Antonie Van Leeuwenhoek. 2022 Nov 16;116(1):1–19. doi: 10.1007/s10482-022-01778-w (PMC9823044; doi:10.1007/s10482-022-01778-w)

Supplementary figure 1A. Left; *ftsZ* Mutant . Right; control strain harbouring a wild-type copy of ftsZ (pNG3 plasmid) in trans in a background wherein the native locus has been inactivated (same as in the mutants FtsZ-AA,EE,EA and AE).

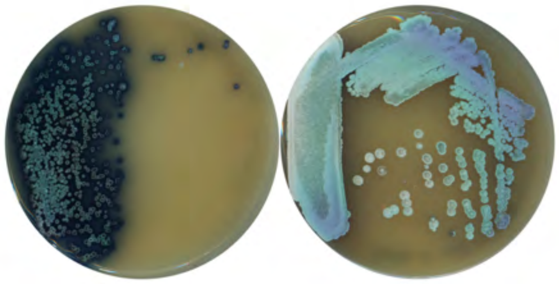

Supplementary figure 1B. Stereo microscope images of the singles colonies. All pictures were taken at same zoom magnification 1X.

Streptomyces coelicolor wild type

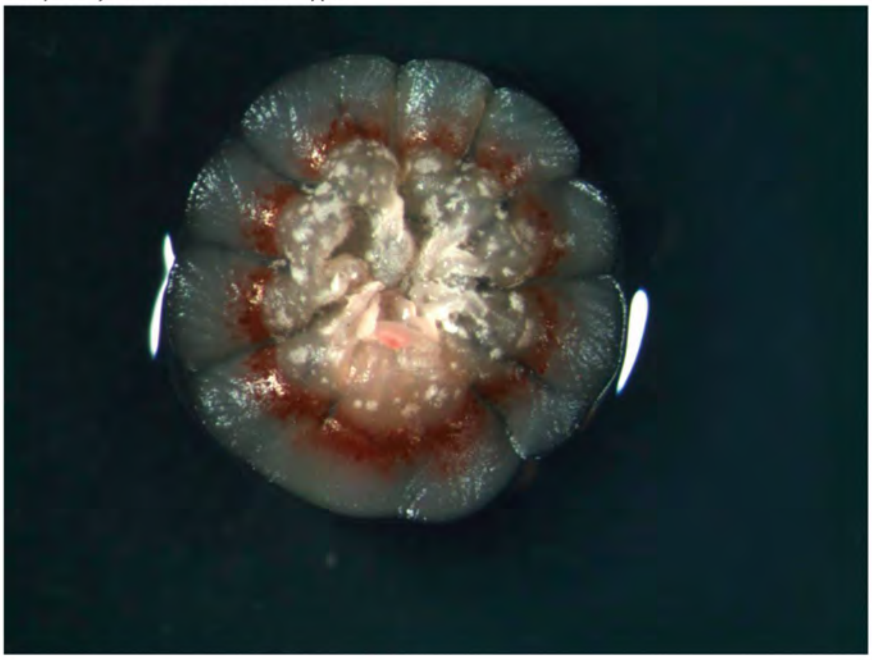

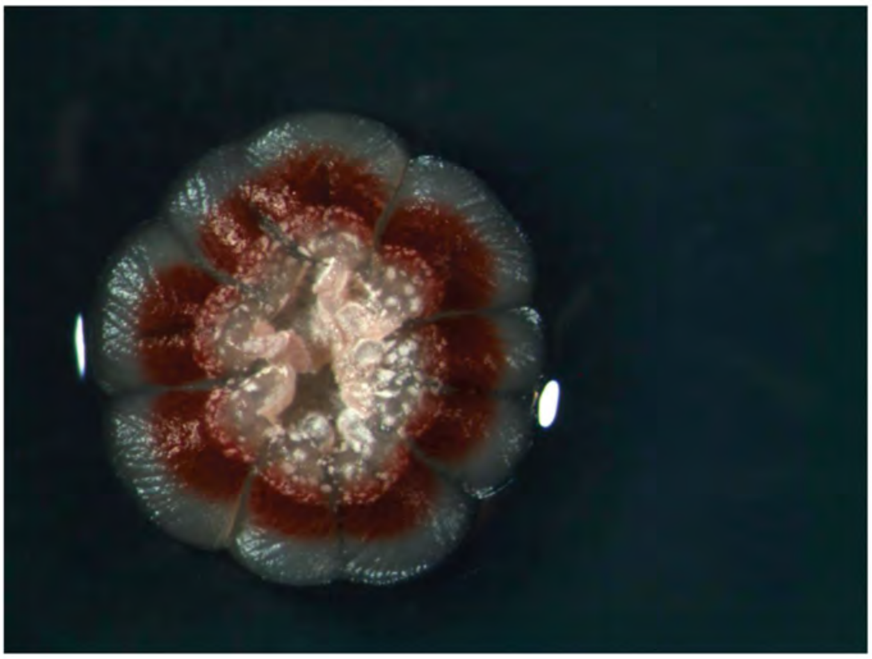

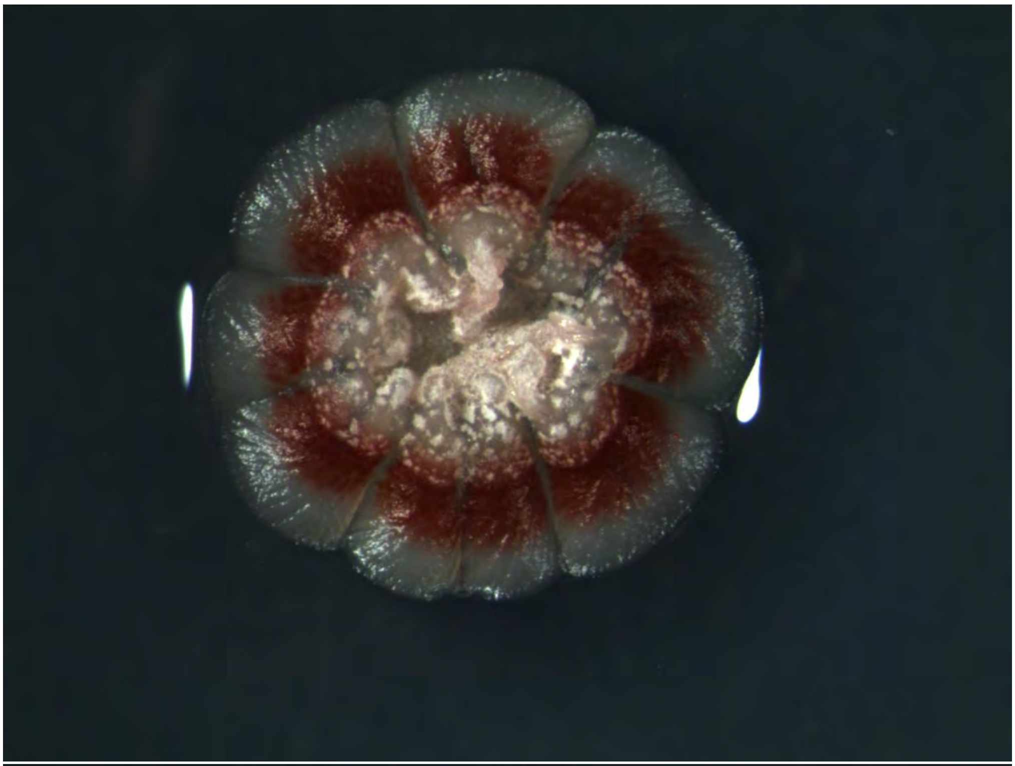
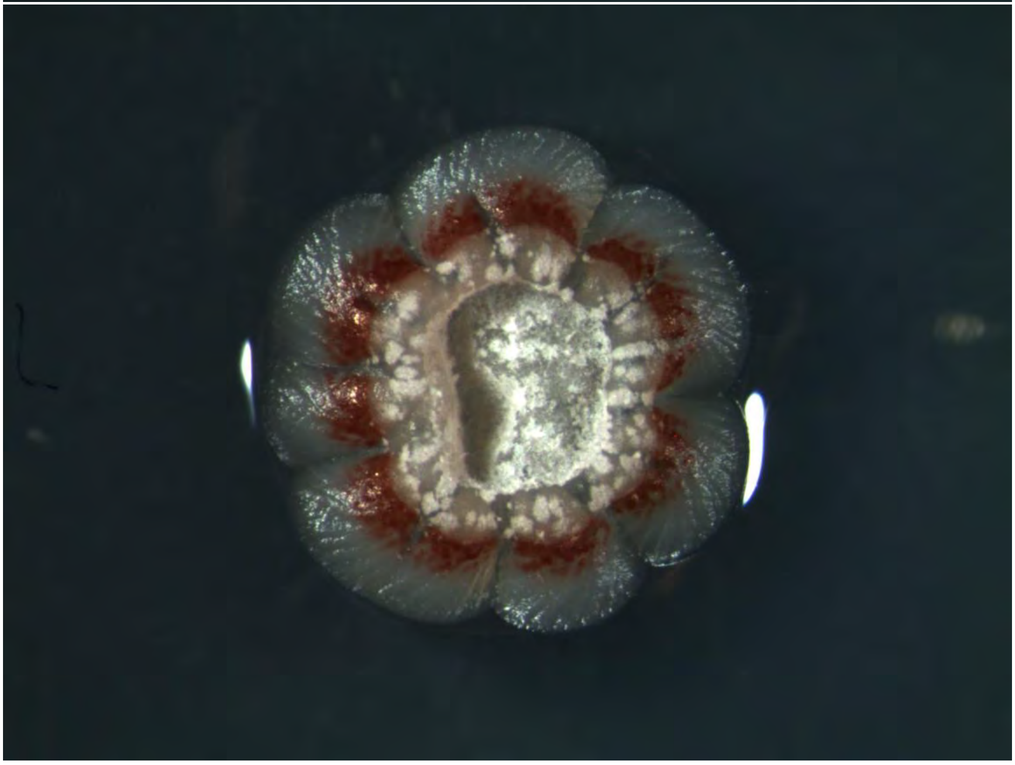

Mutant 1 (EE)

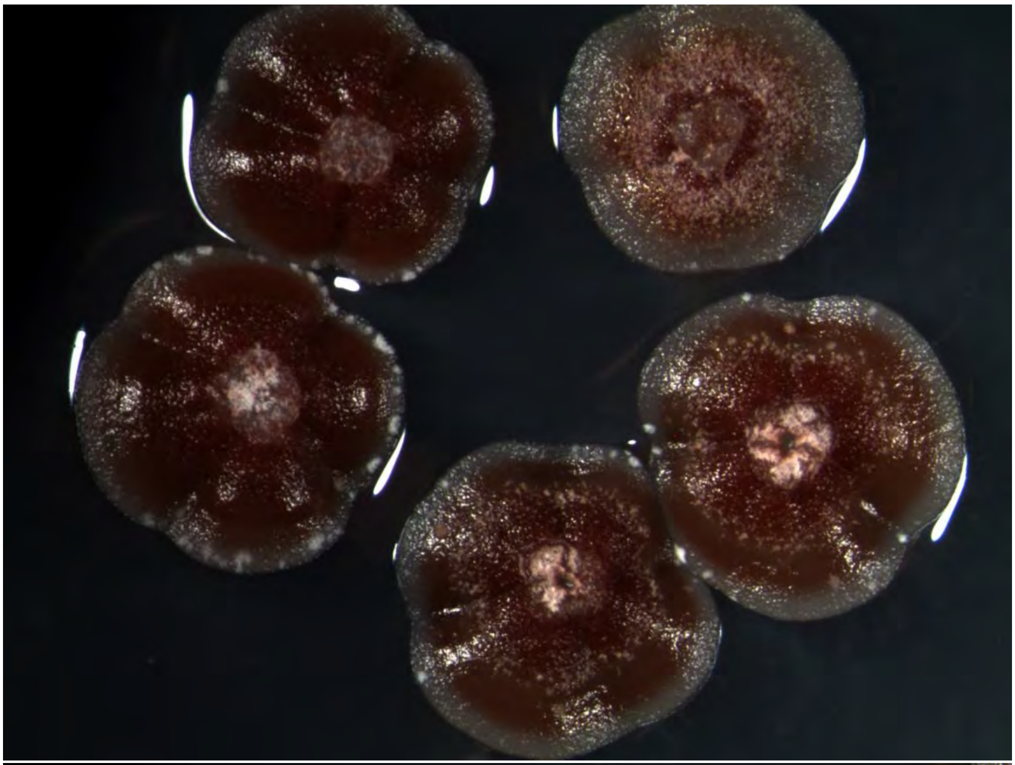

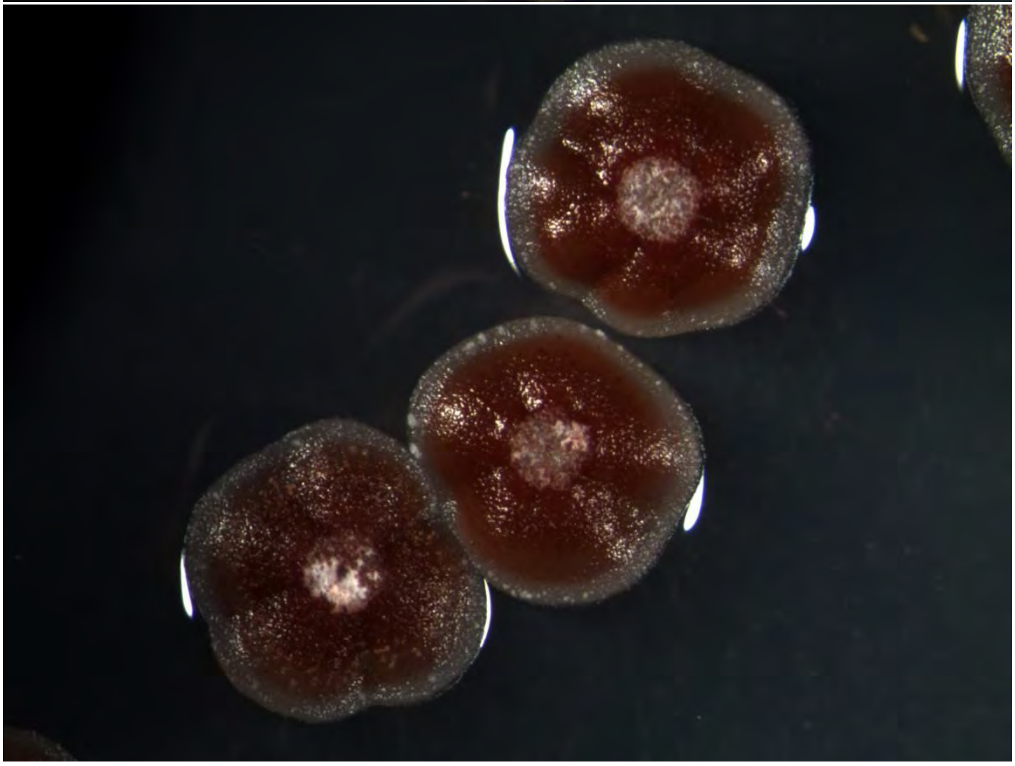

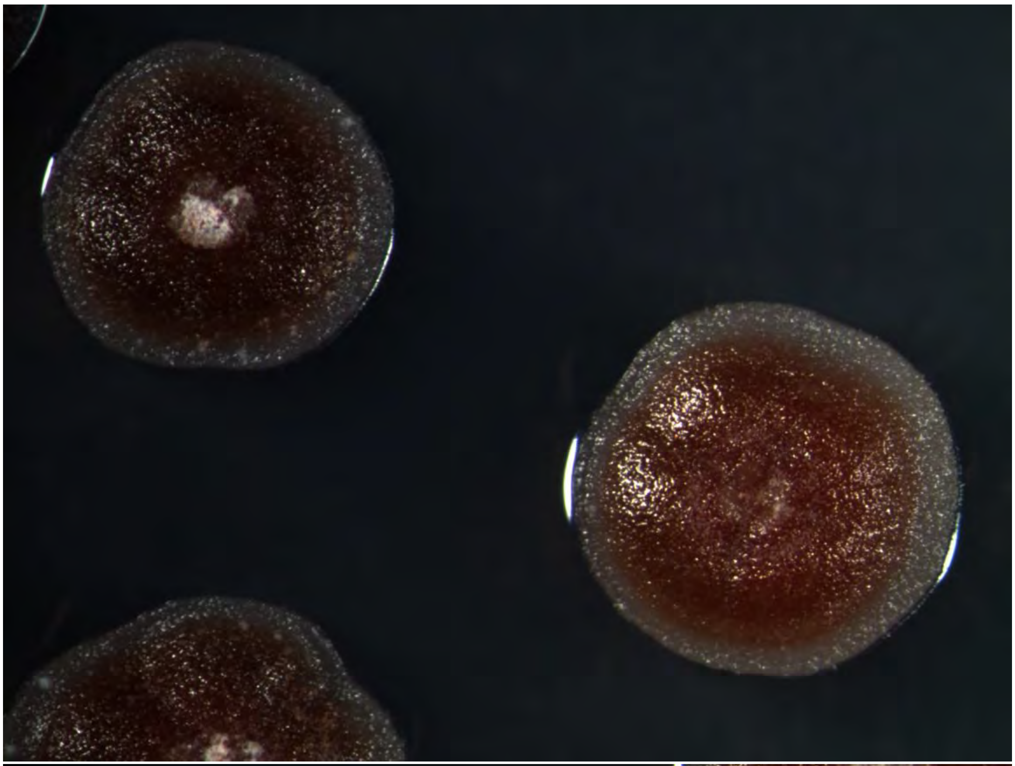
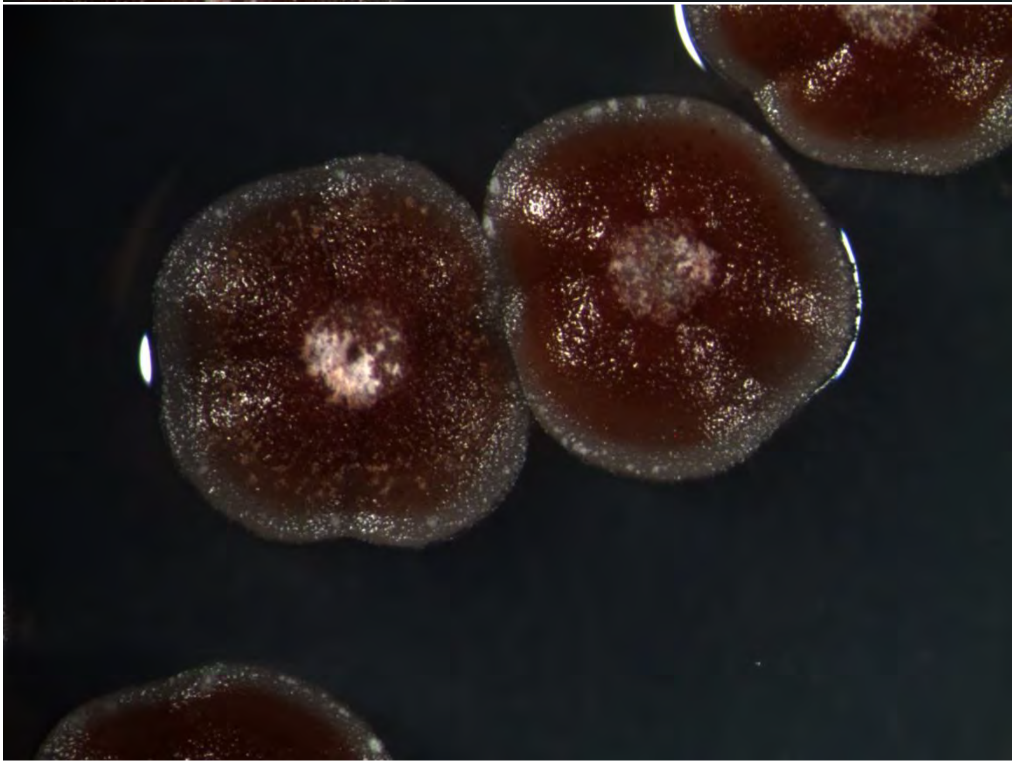

Mutant 2 (AA)

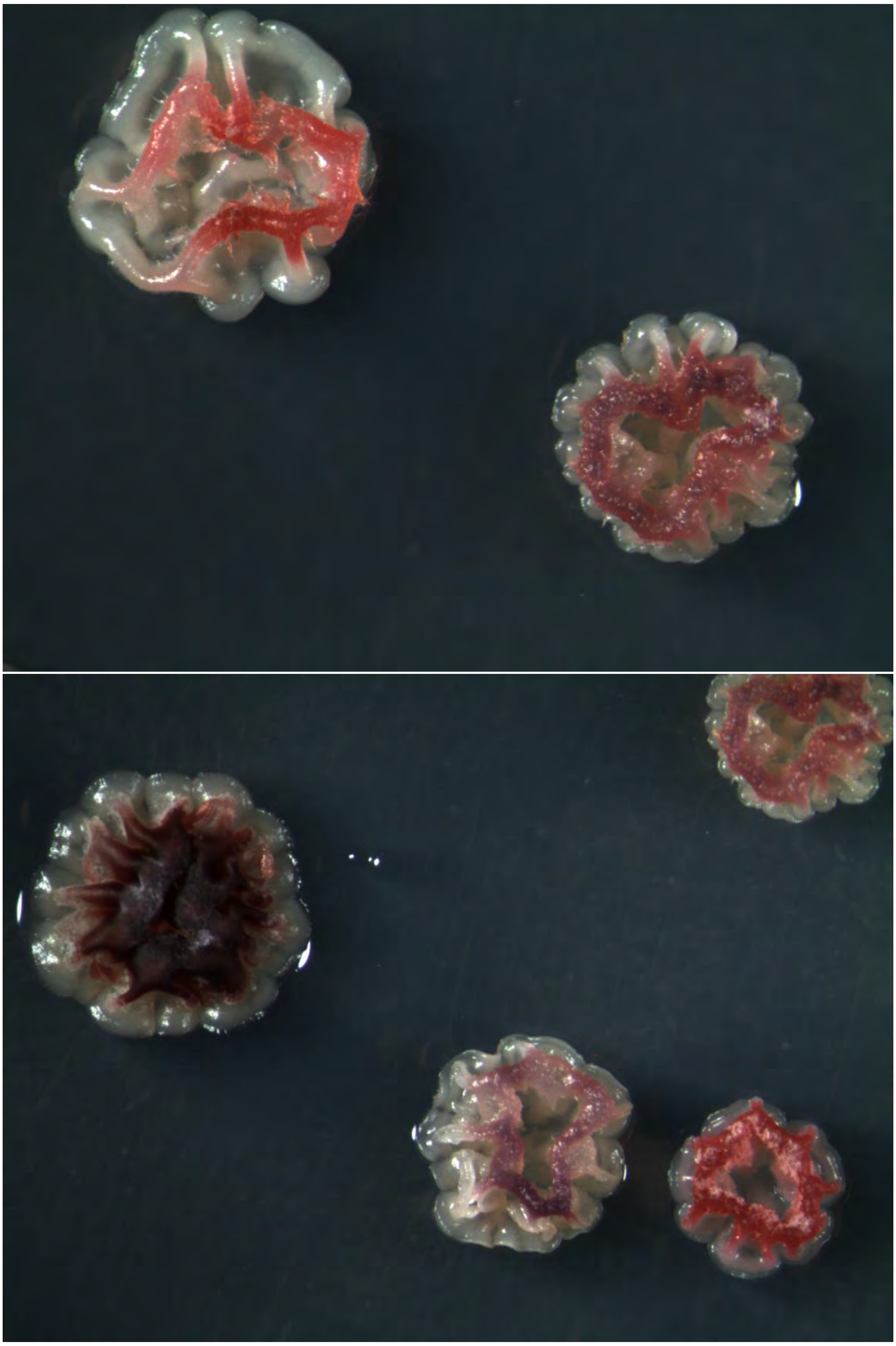

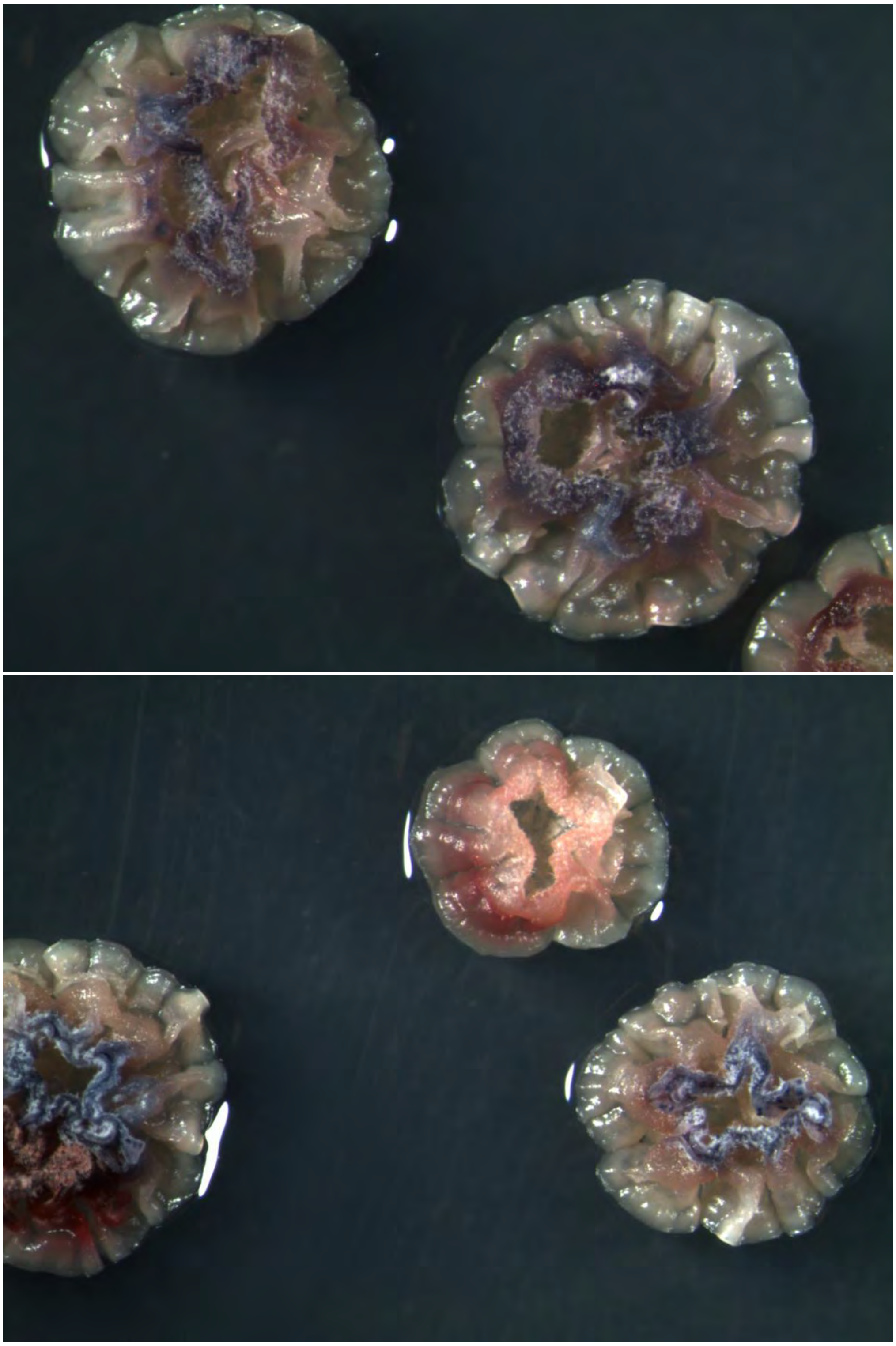

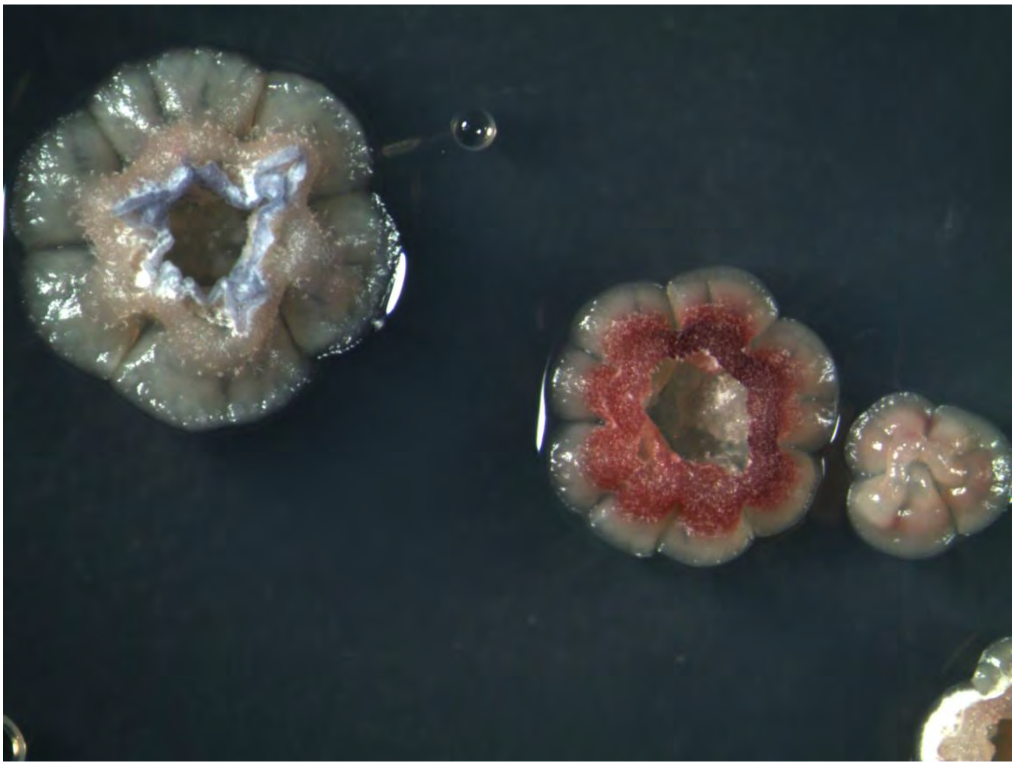

Mutant 3 (EA)

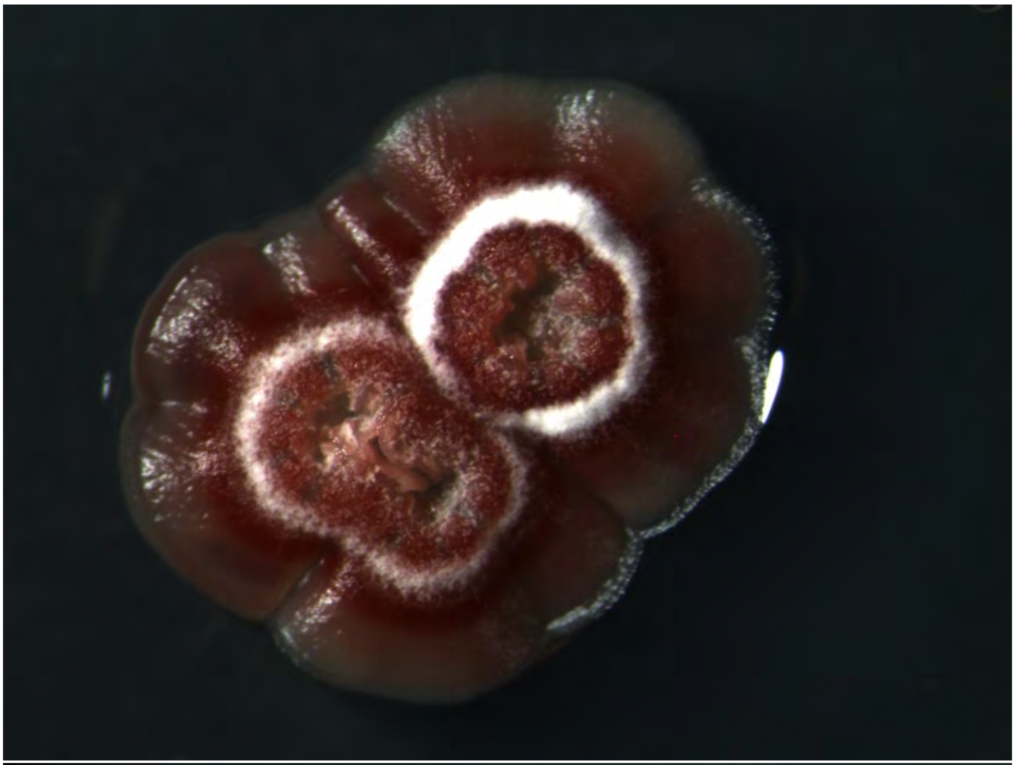

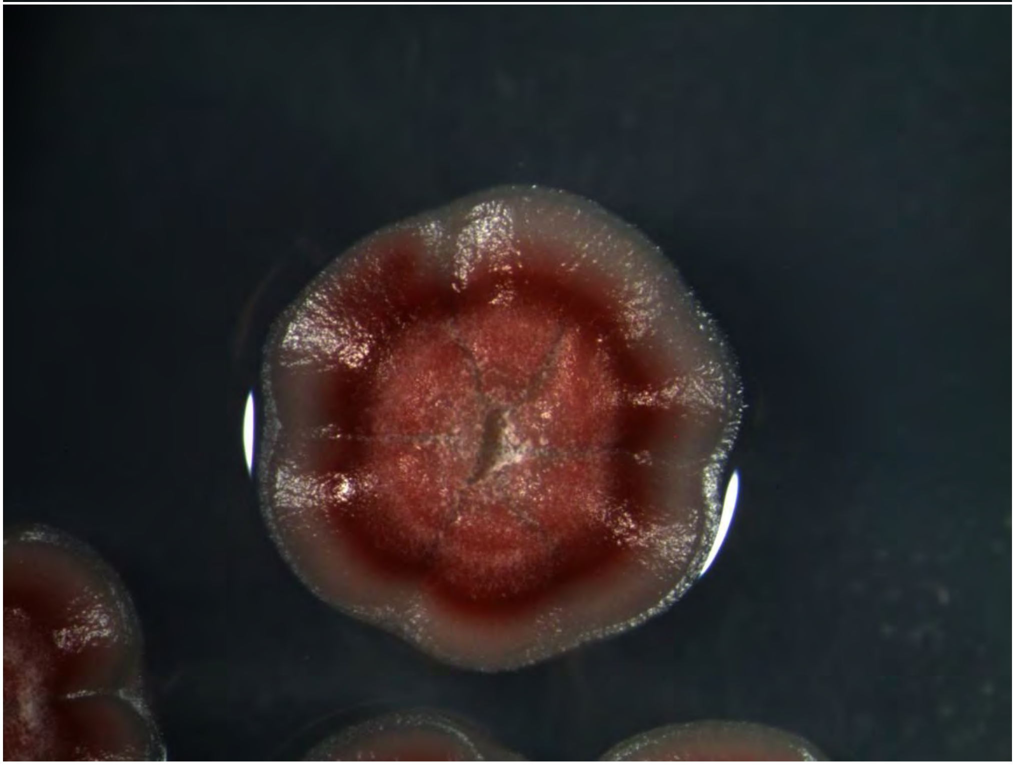

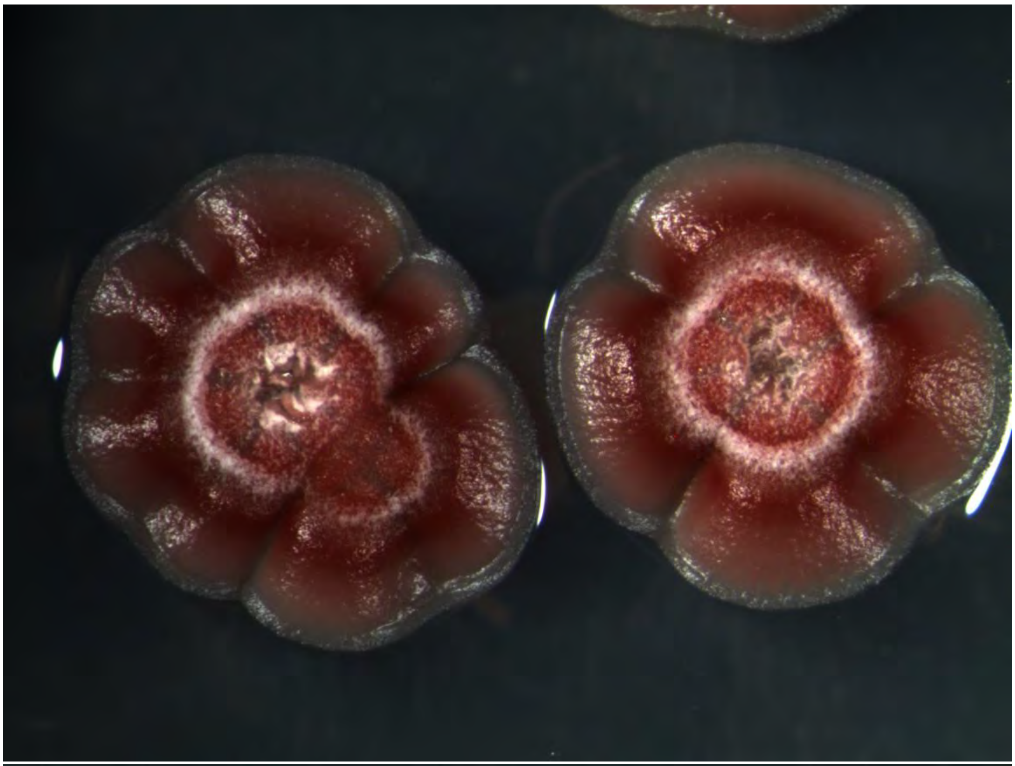

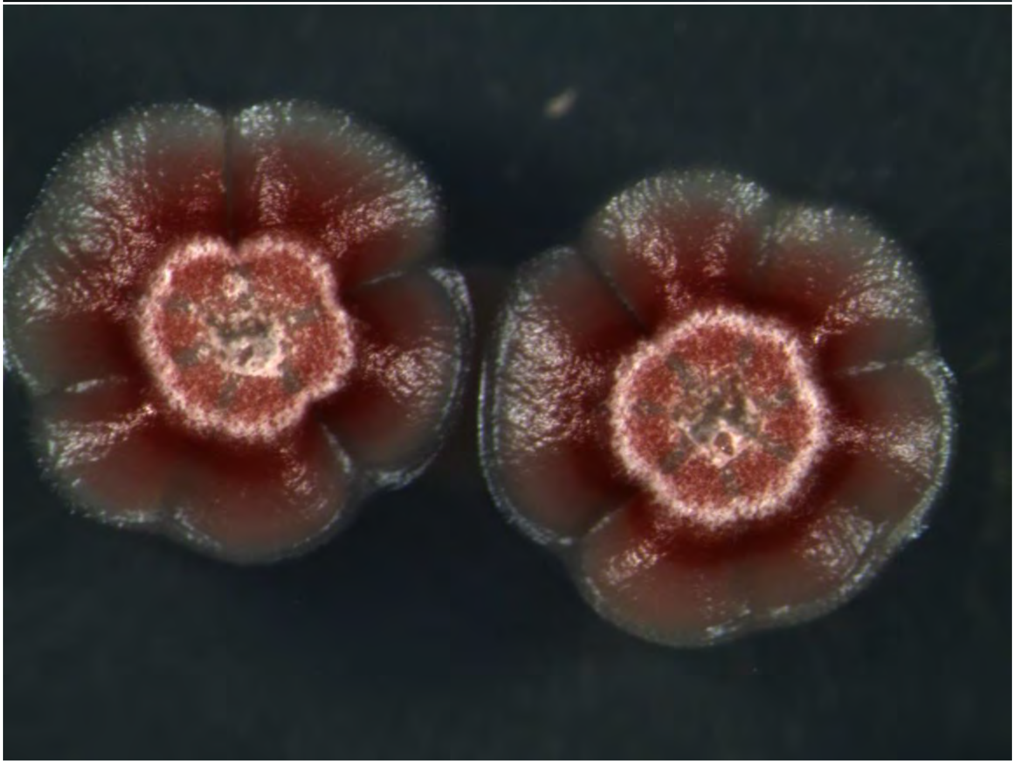

Mutant 4 (AE)

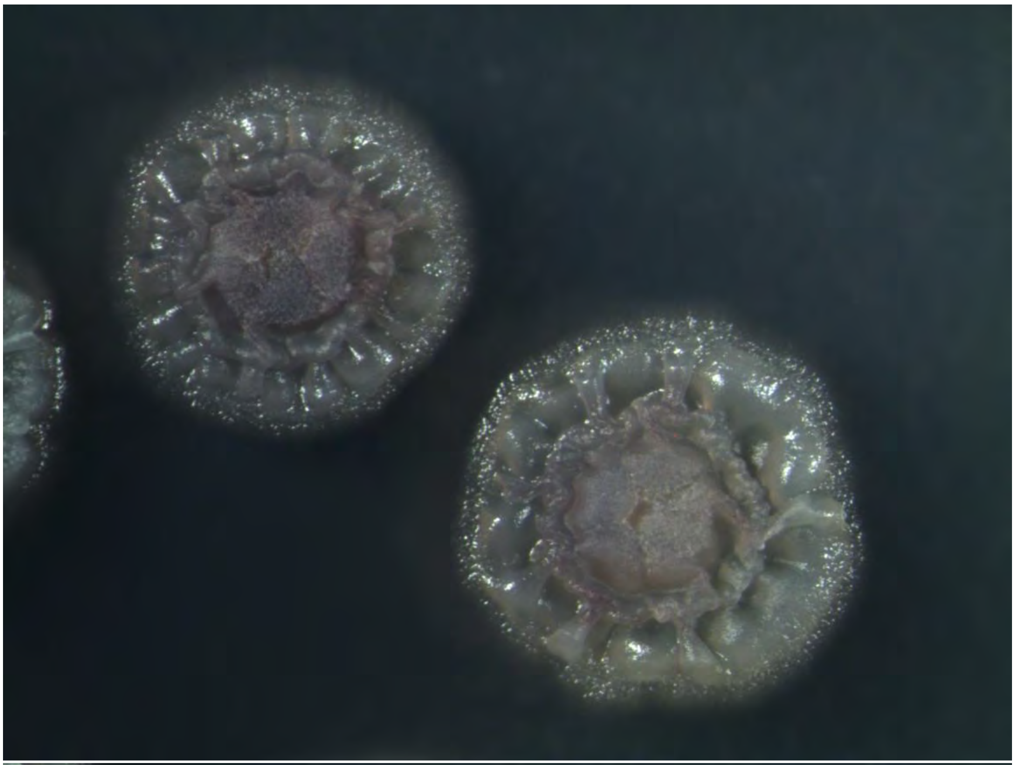

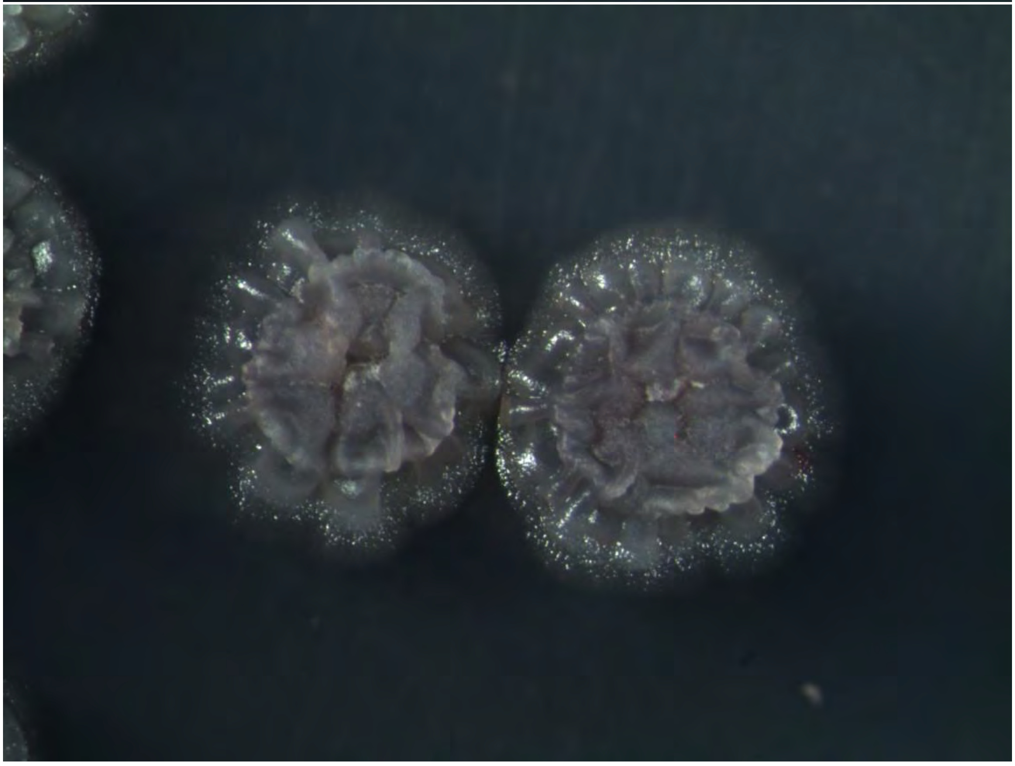

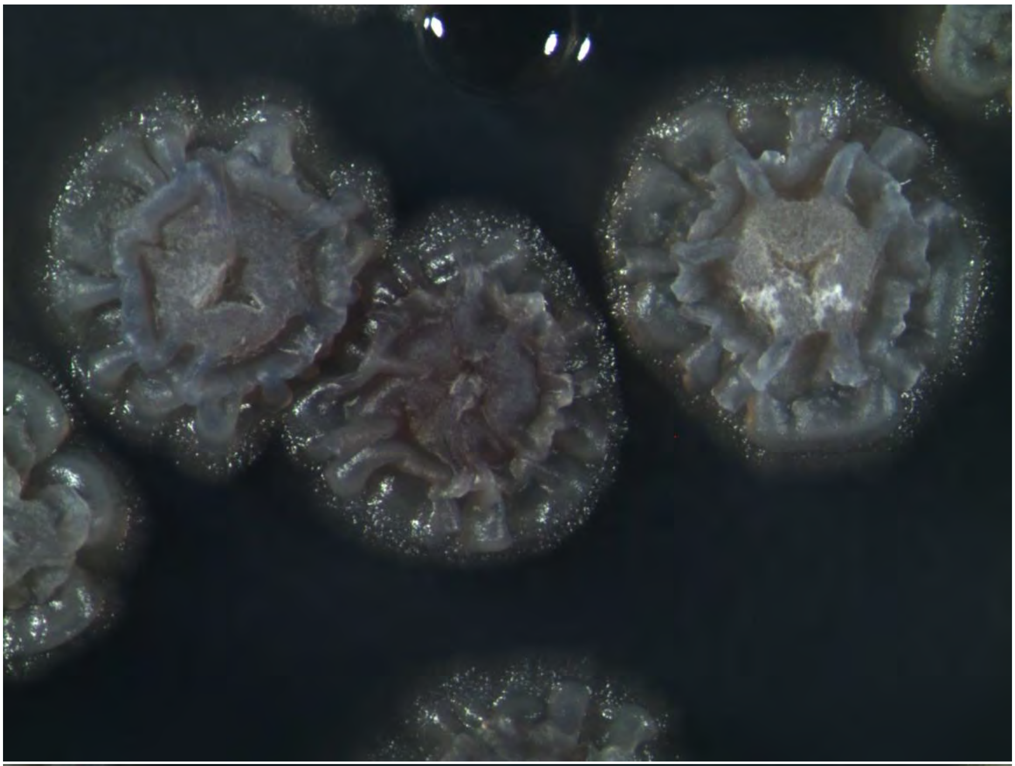

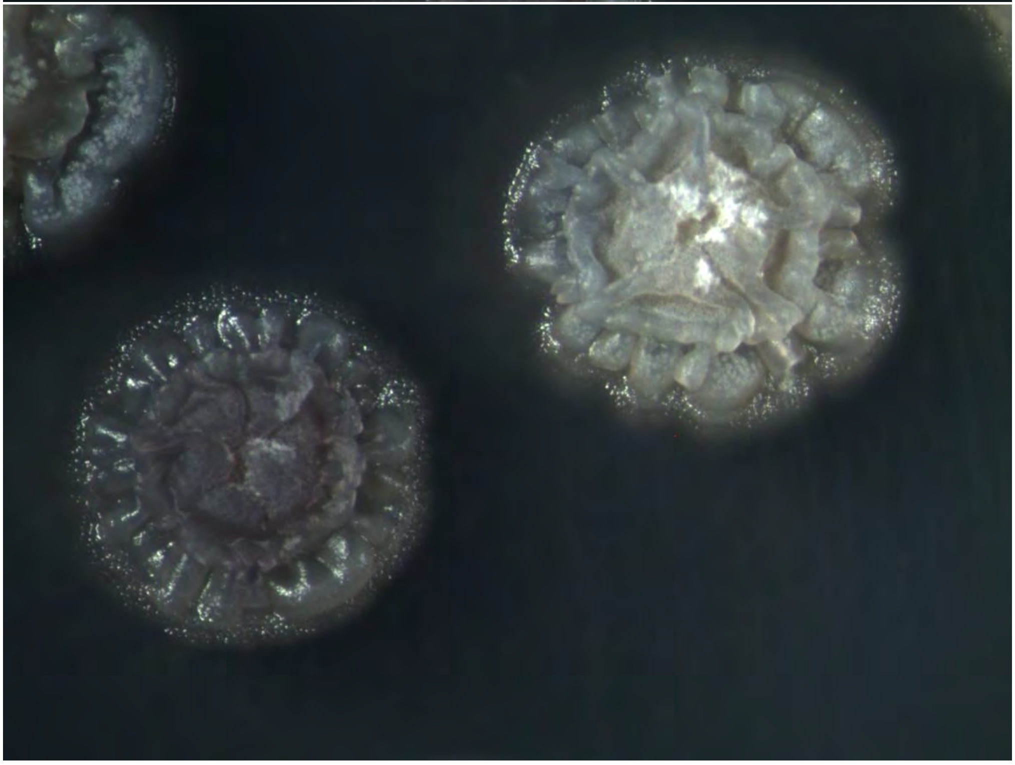

Supplement: Supplementary file 1 — Supplementary file1 (PDF 6291 KB) [file 10482_2022_1778_MOESM1_ESM.pdf]
