# Supplementary material for: FtsZ phosphorylation pleiotropically affects Z-ladder formation, antibiotic production, and morphogenesis in Streptomyces coelicolor
Source: Antonie Van Leeuwenhoek. 2022 Nov 16;116(1):1–19. doi: 10.1007/s10482-022-01778-w (PMC9823044; doi:10.1007/s10482-022-01778-w)
Supplement: Supplementary file 2 — Supplementary file2 (PDF 4605 KB) [file 10482_2022_1778_MOESM2_ESM.pdf]

Supplementary figure 2. The scanning electron microscopy images used for measuring spore lengths.

***Streptomyces coelicolor* wild type**

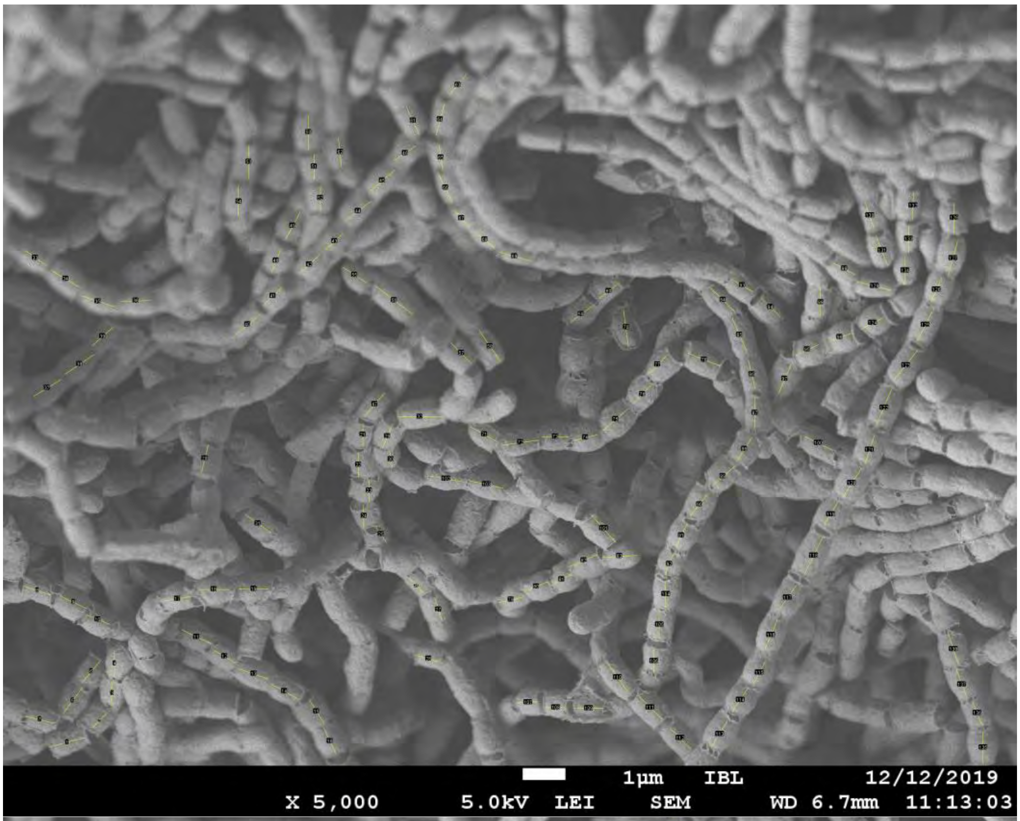

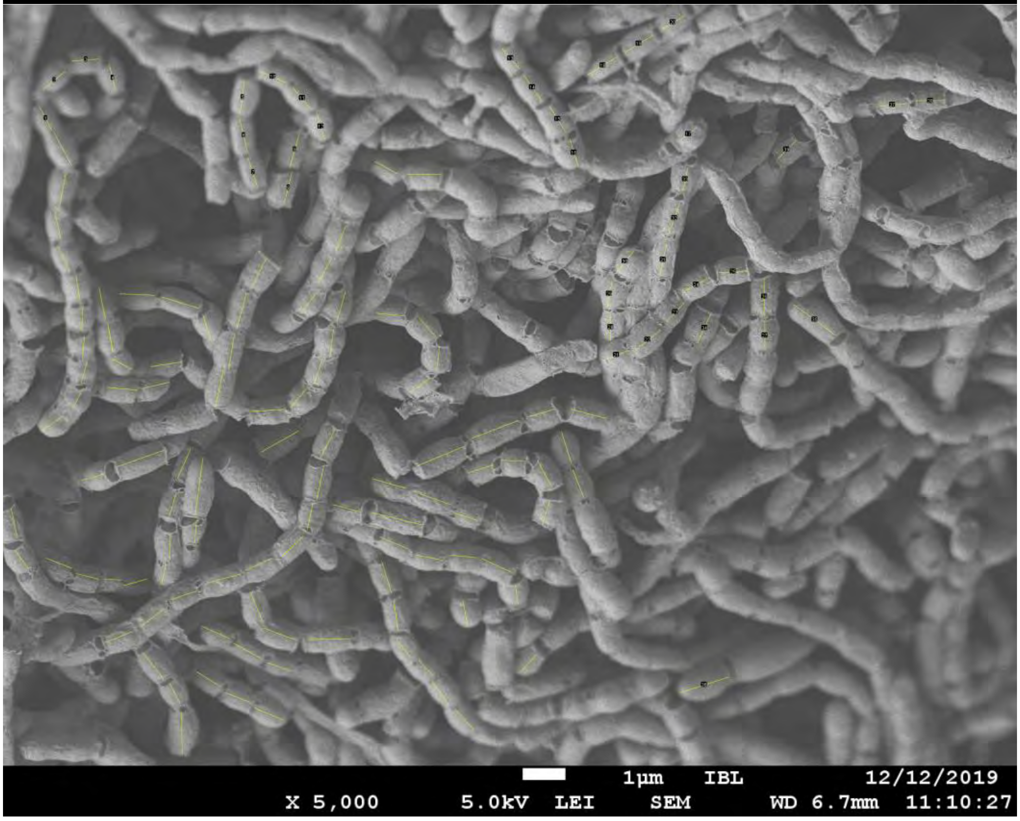

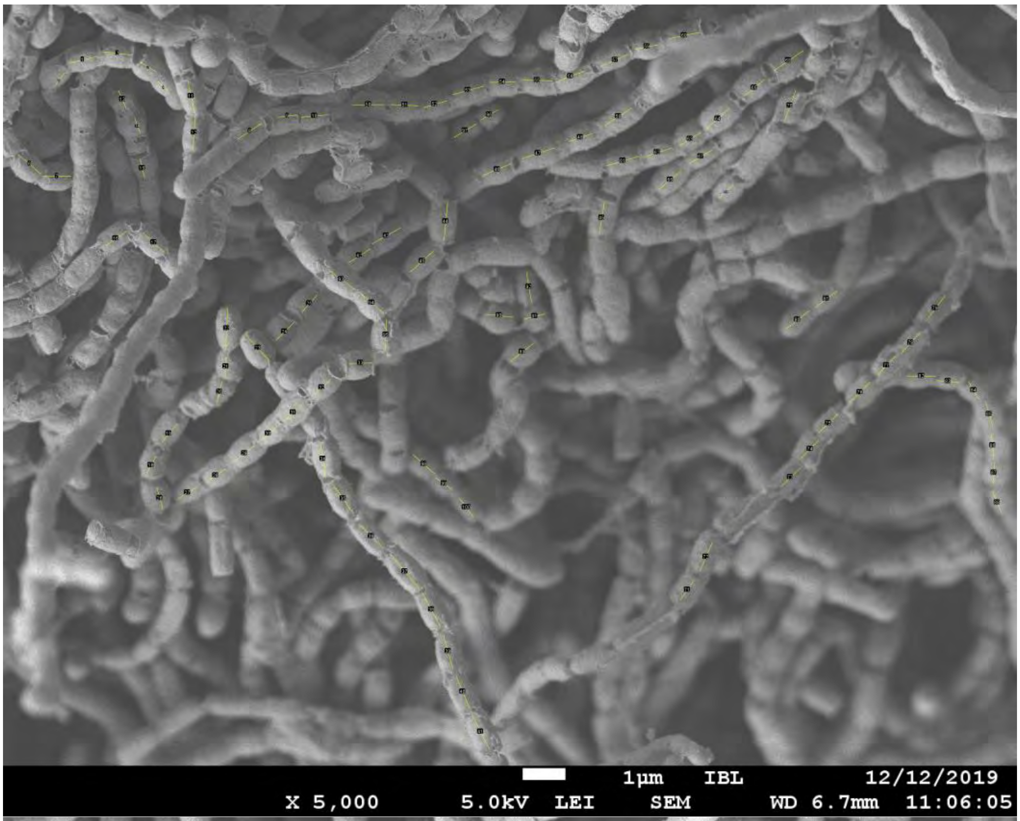
1µm IBL 12/12/2019
X 5,000 5.0kV LEI SEM WD 6.7mm 11:06:05
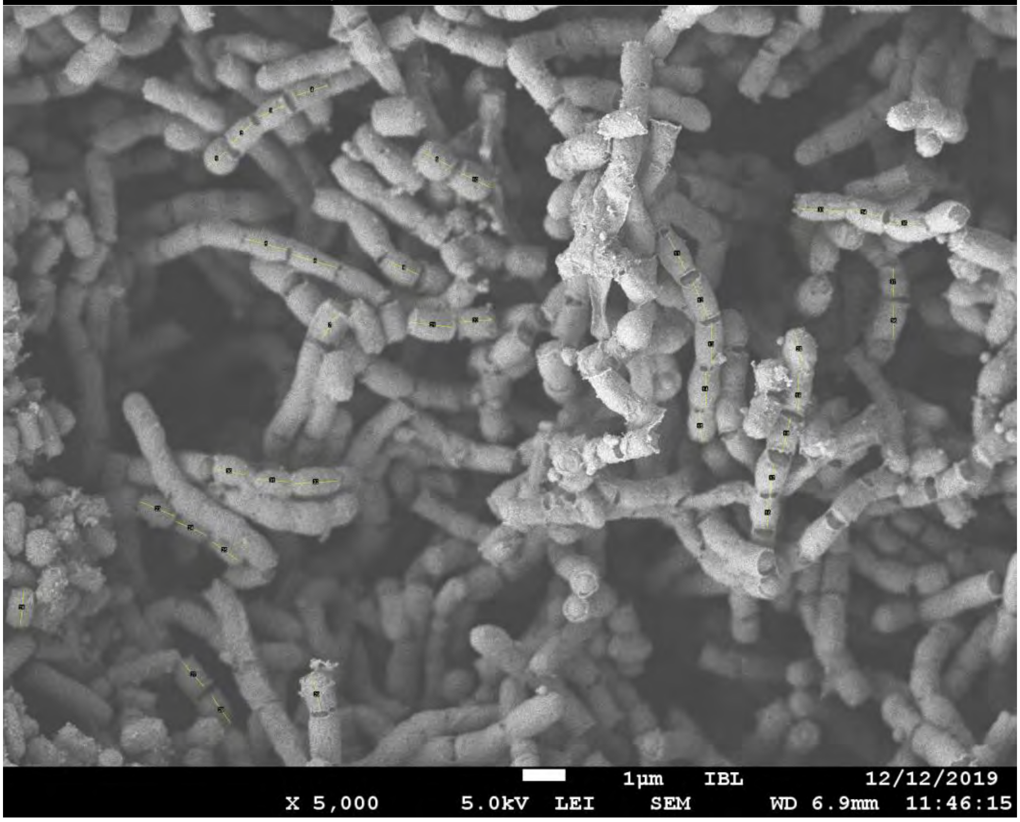
1µm IBL 12/12/2019
X 5,000 5.0kV LEI SEM WD 6.9mm 11:46:15

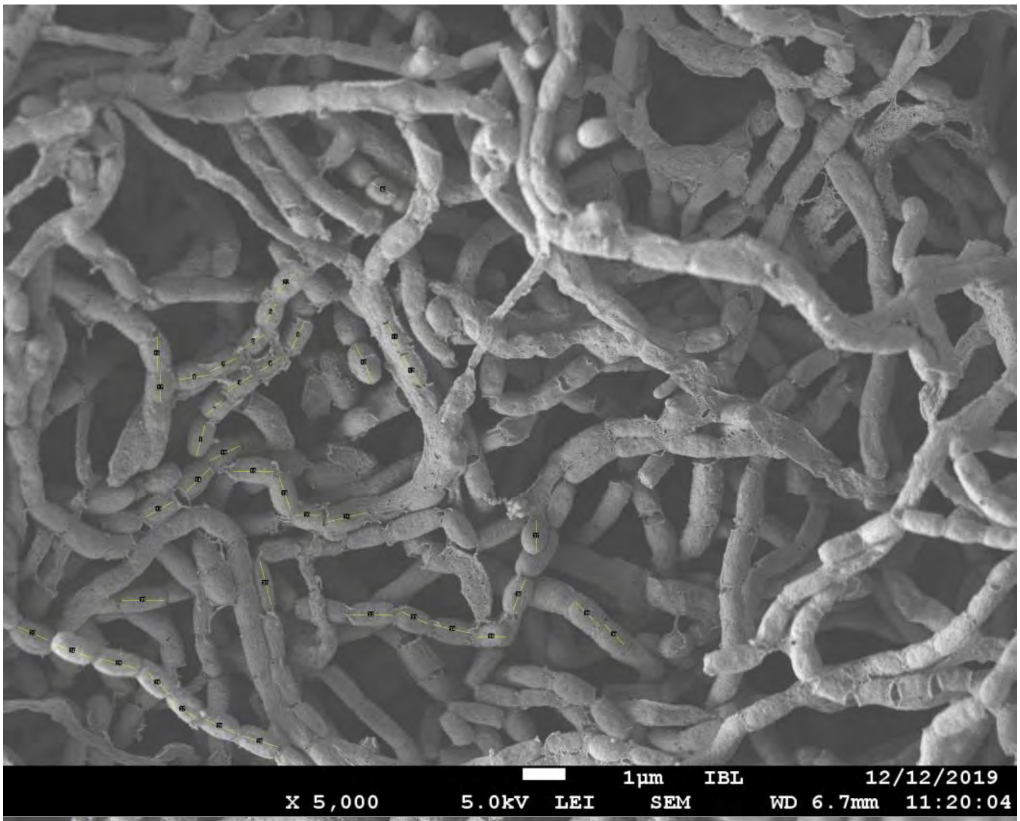
1µm IBL 12/12/2019
X 5,000 5.0kV LEI SEM WD 6.7mm 11:20:04

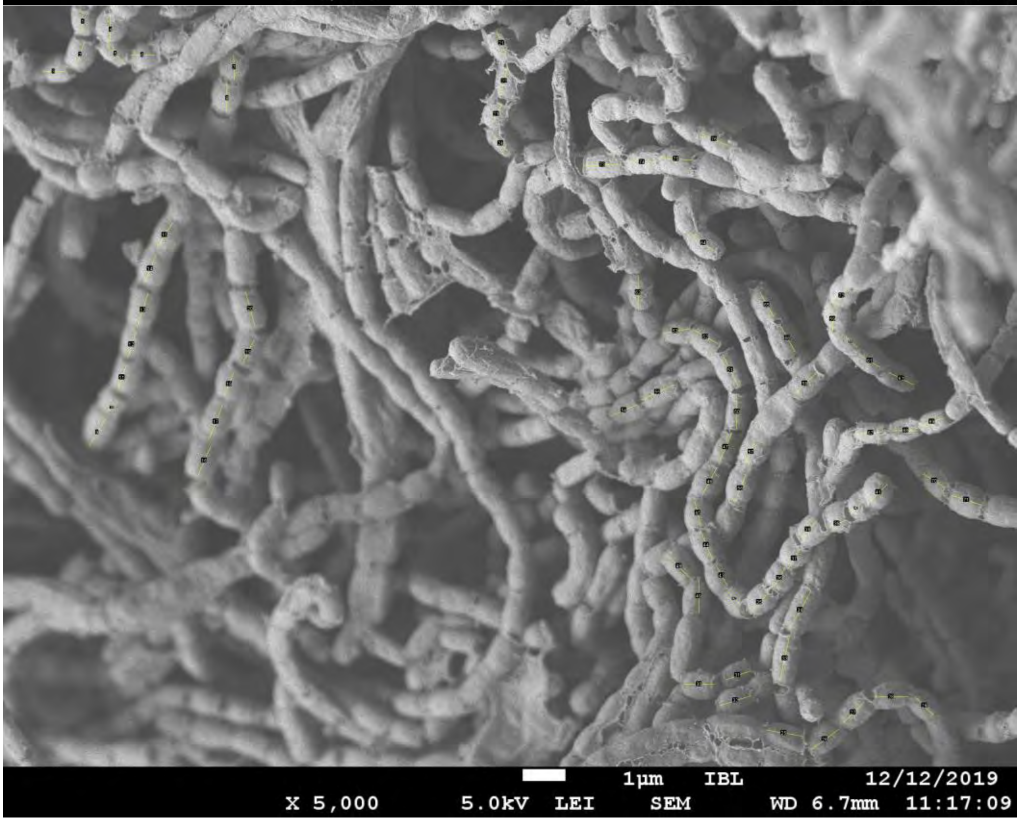
1µm IBL 12/12/2019
X 5,000 5.0kV LEI SEM WD 6.7mm 11:17:09

Mutant 1 (EE)

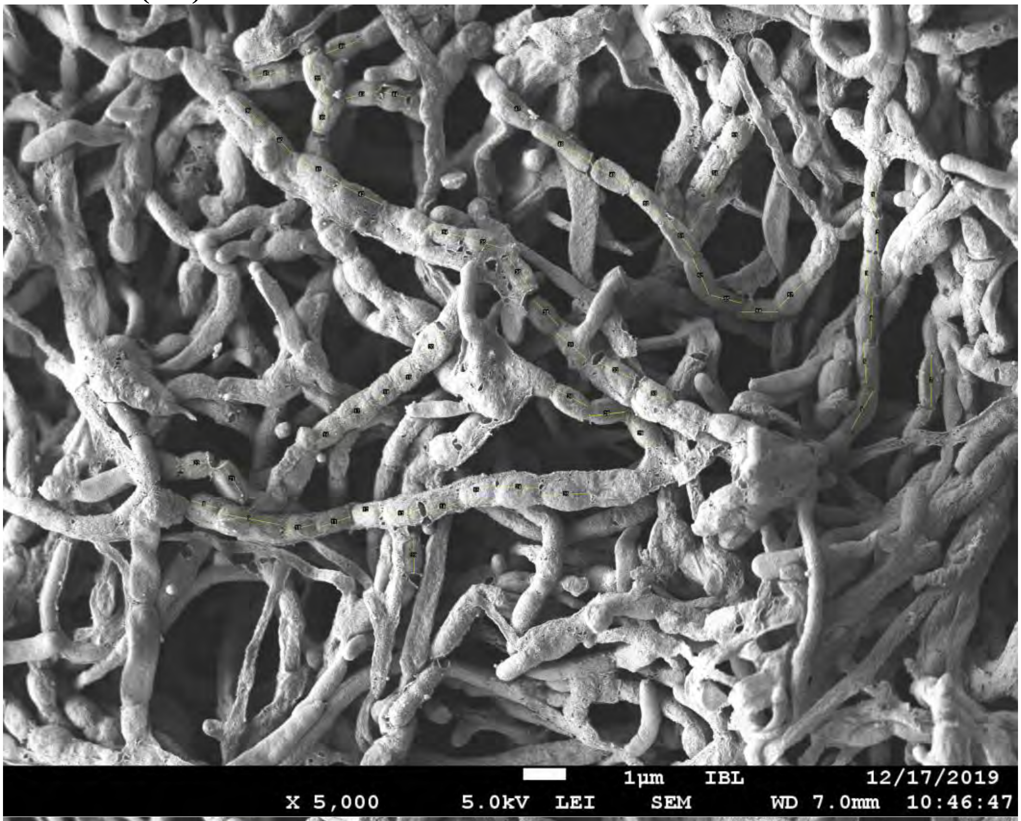
1µm IBL 12/17/2019
X 5,000 5.0kV LEI SEM WD 7.0mm 10:46:47

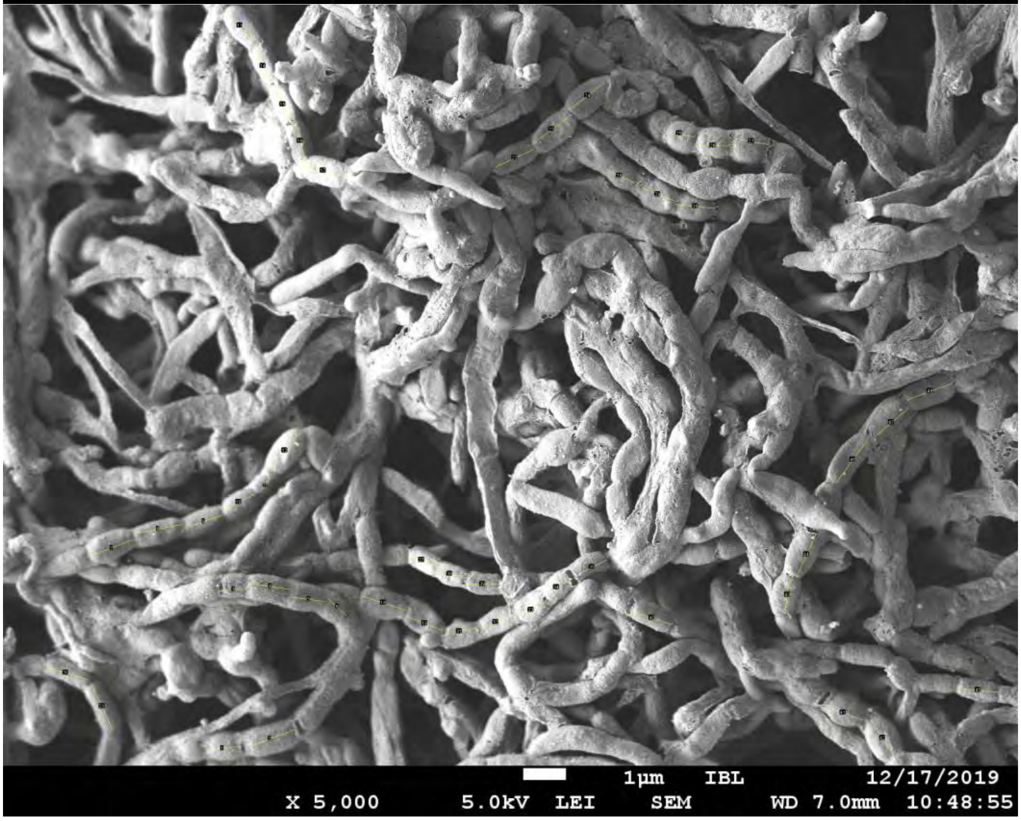
1µm IBL 12/17/2019
X 5,000 5.0kV LEI SEM WD 7.0mm 10:48:55

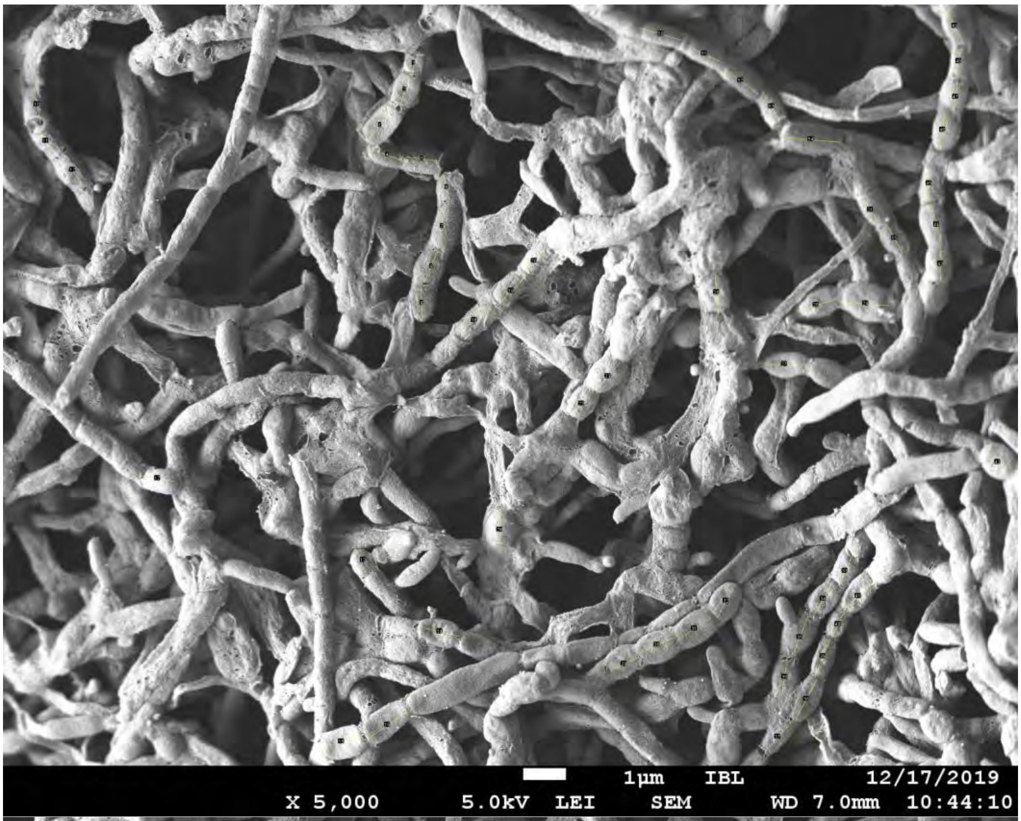
1µm IBL 12/17/2019
X 5,000 5.0kV LEI SEM WD 7.0mm 10:44:10

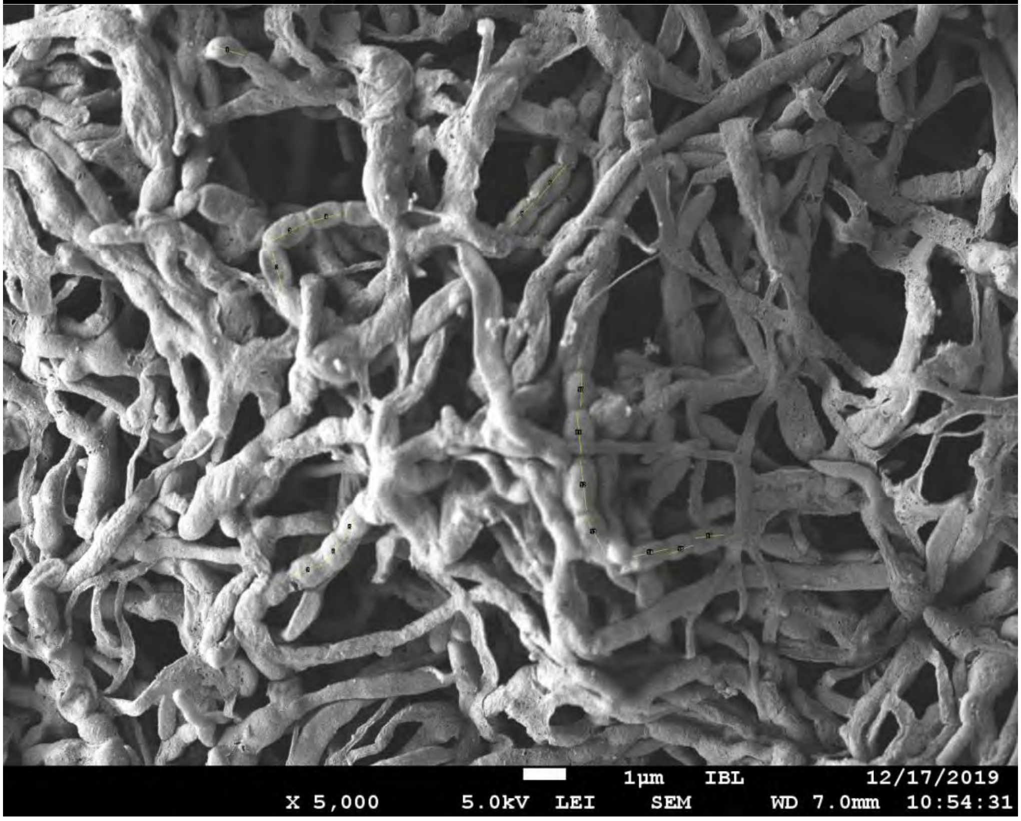
1µm IBL 12/17/2019
X 5,000 5.0kV LEI SEM WD 7.0mm 10:54:31

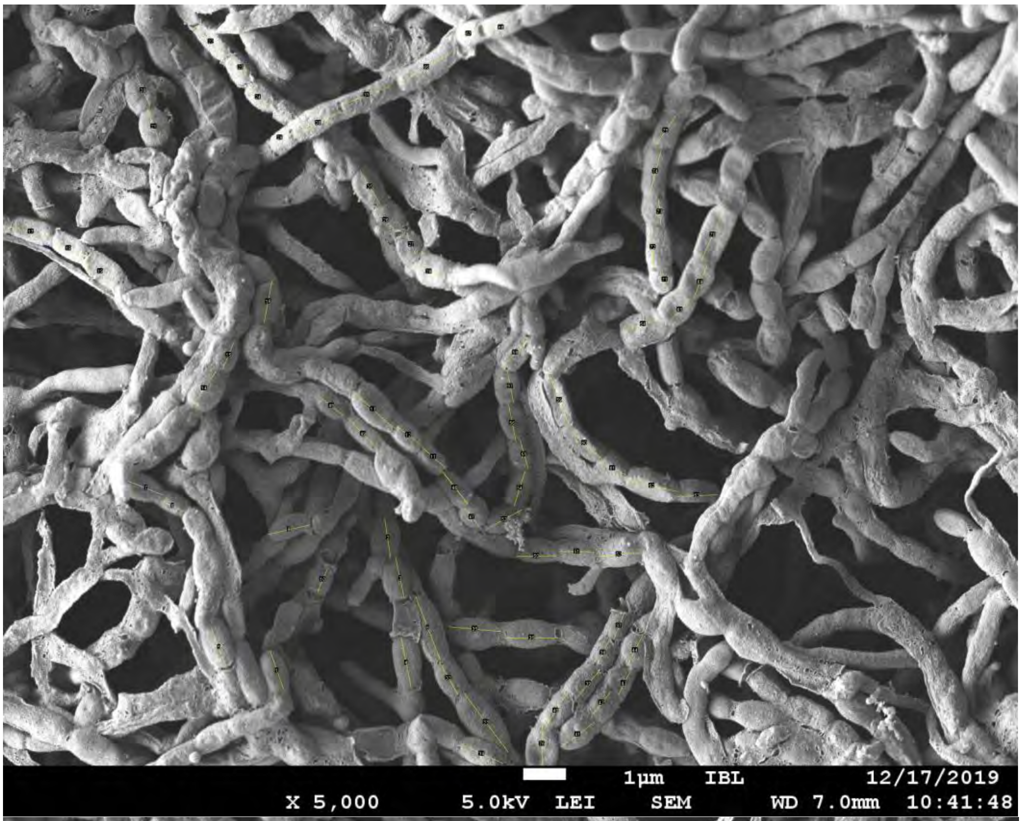
1µm IBL 12/17/2019
X 5,000 5.0kV LEI SEM WD 7.0mm 10:41:48

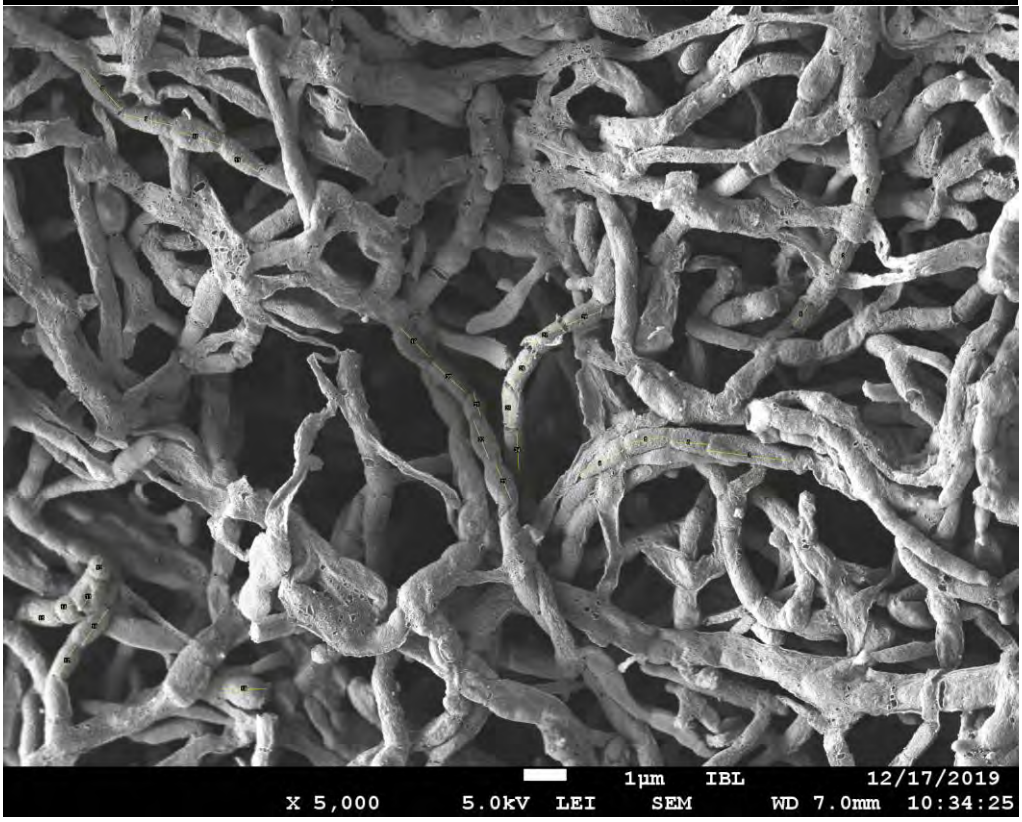
1µm IBL 12/17/2019
X 5,000 5.0kV LEI SEM WD 7.0mm 10:34:25

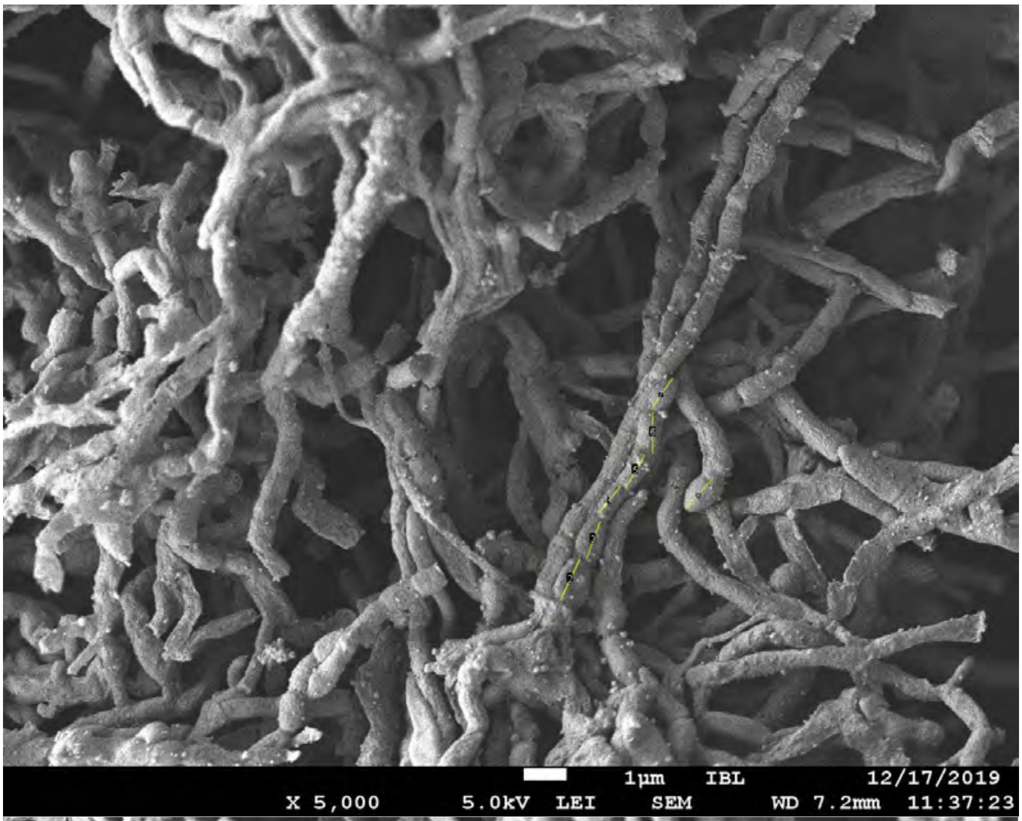
1µm IBL 12/17/2019
X 5,000 5.0kV LEI SEM WD 7.2mm 11:37:23

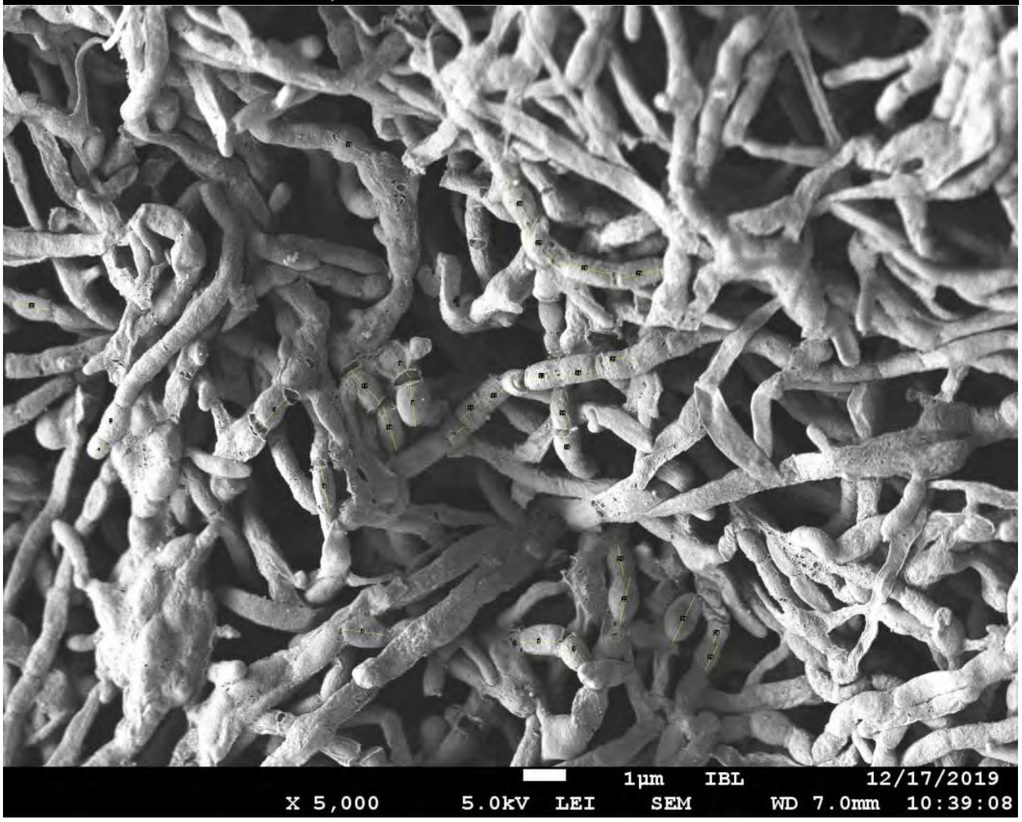
1µm IBL 12/17/2019
X 5,000 5.0kV LEI SEM WD 7.0mm 10:39:08

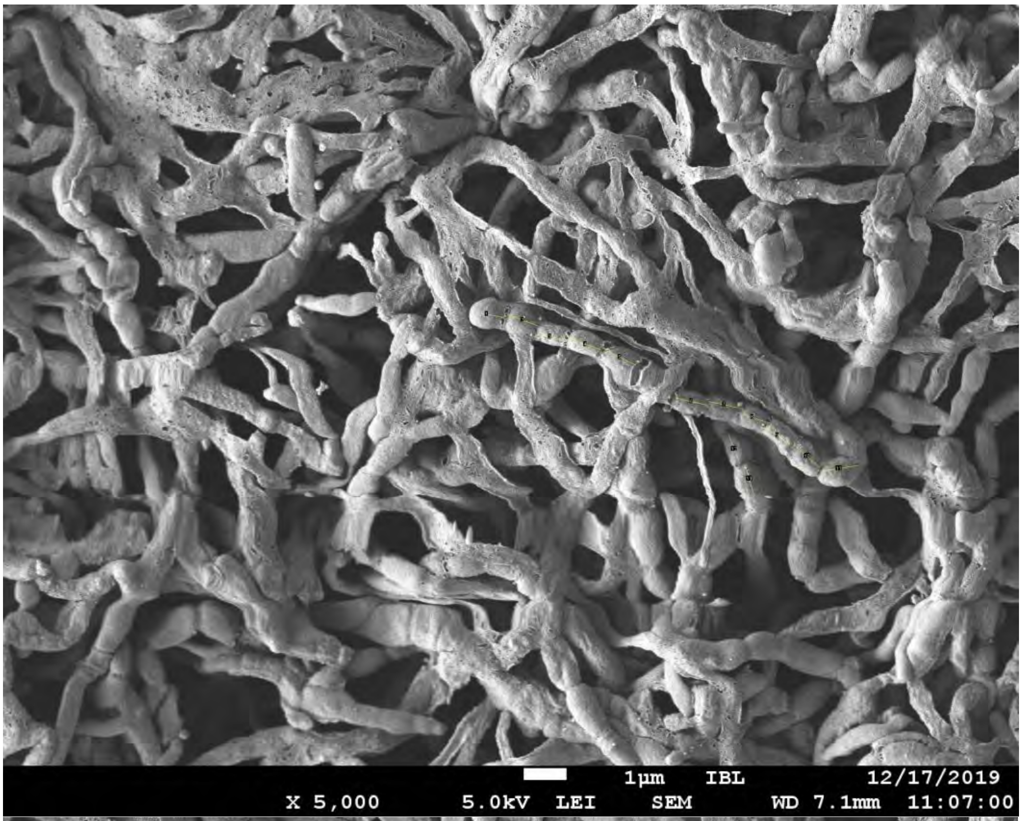
1µm IBL 12/17/2019
X 5,000 5.0kV LEI SEM WD 7.1mm 11:07:00

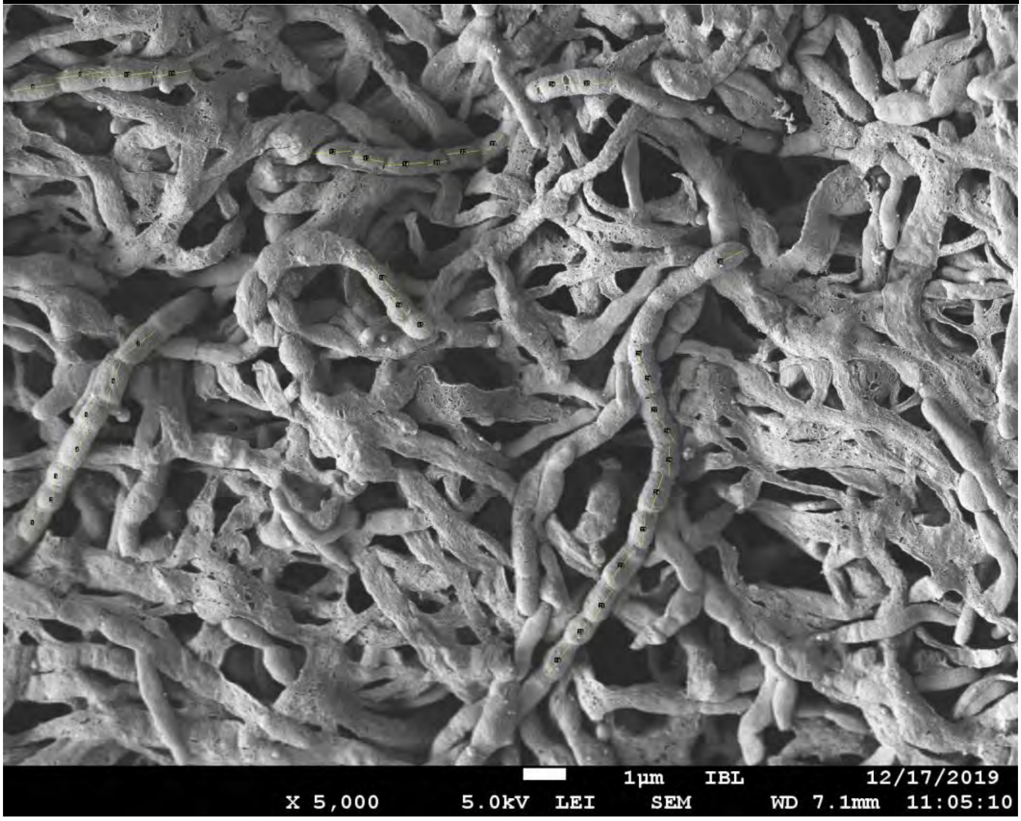
1µm IBL 12/17/2019
X 5,000 5.0kV LEI SEM WD 7.1mm 11:05:10

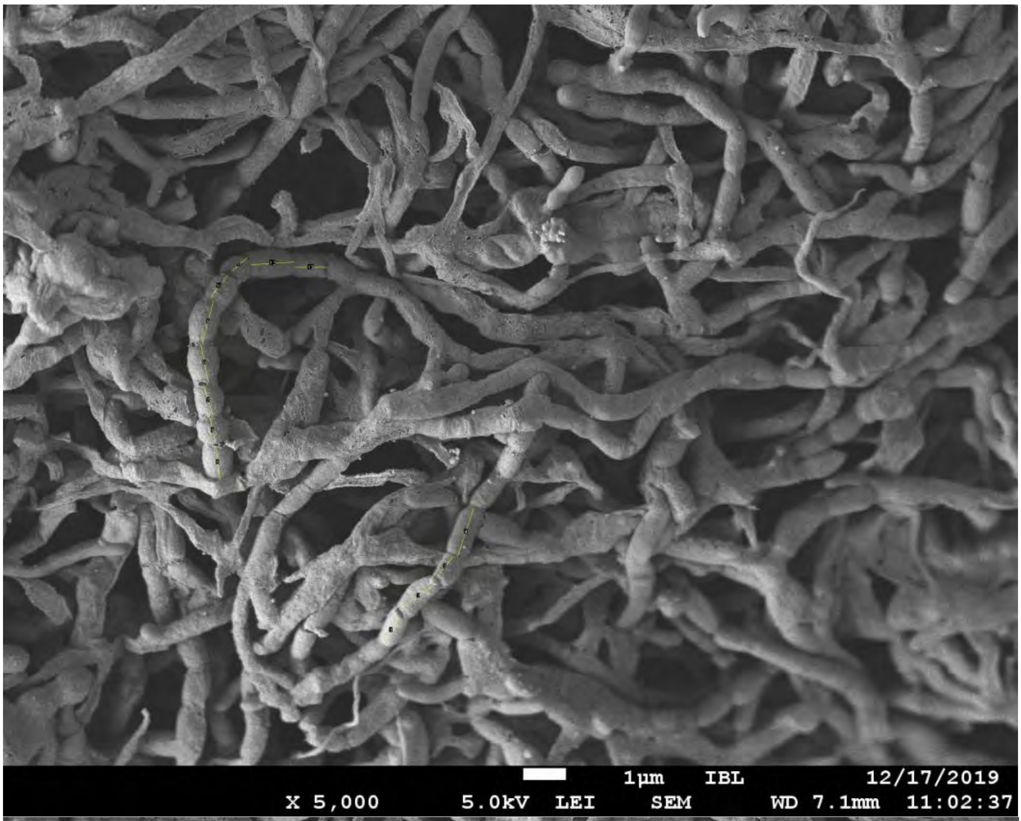
1μm IBL 12/17/2019
X 5,000 5.0kV LEI SEM WD 7.1mm 11:02:37

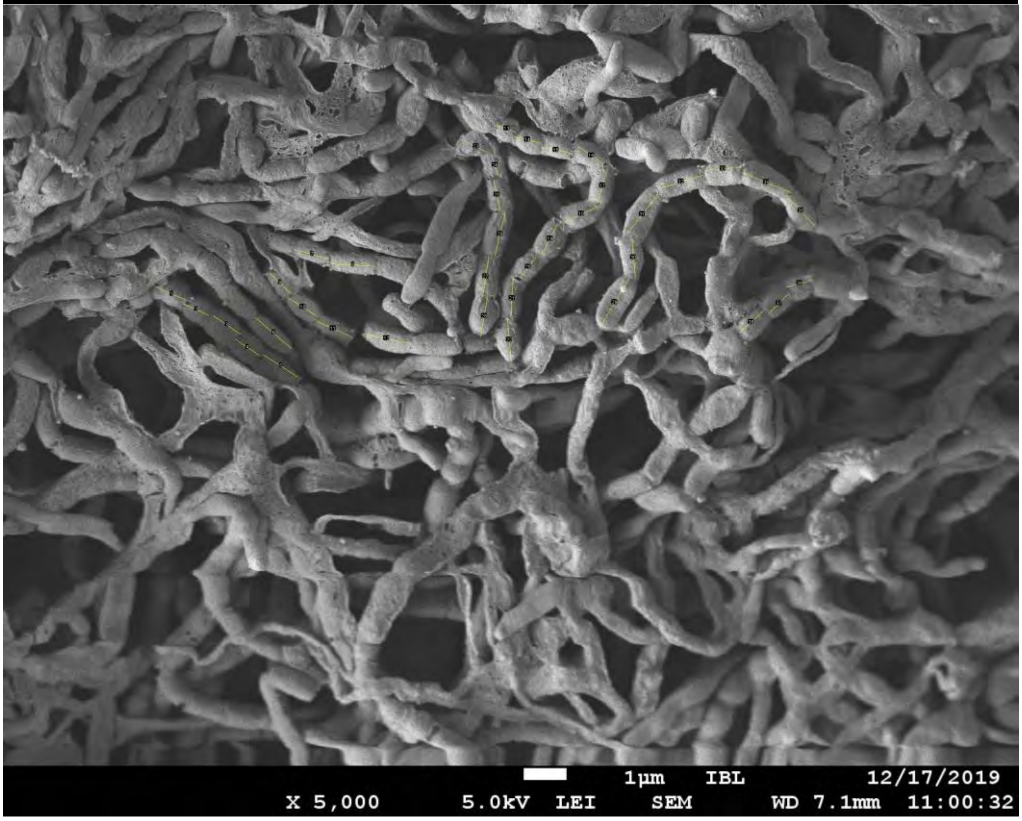
1μm IBL 12/17/2019
X 5,000 5.0kV LEI SEM WD 7.1mm 11:00:32

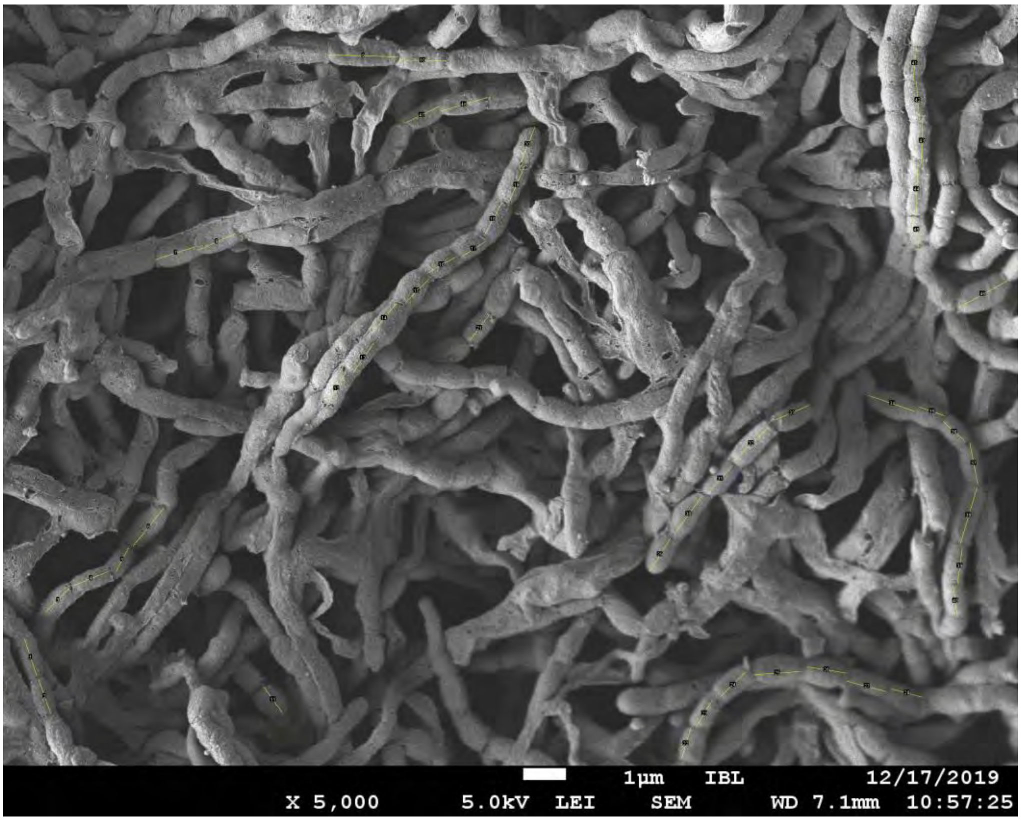
1μm IBL 12/17/2019
X 5,000 5.0kV LEI SEM WD 7.1mm 10:57:25

Mutant 3 (EA)

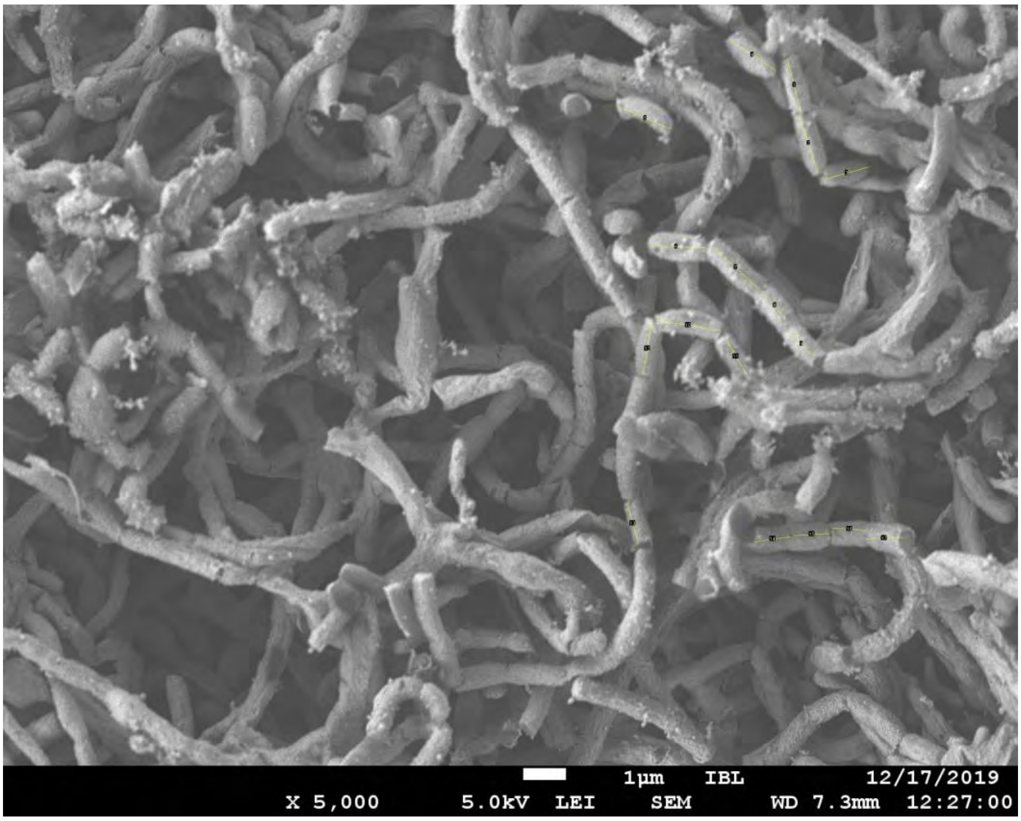
1μm IBL 12/17/2019
X 5,000 5.0kV LEI SEM WD 7.3mm 12:27:00

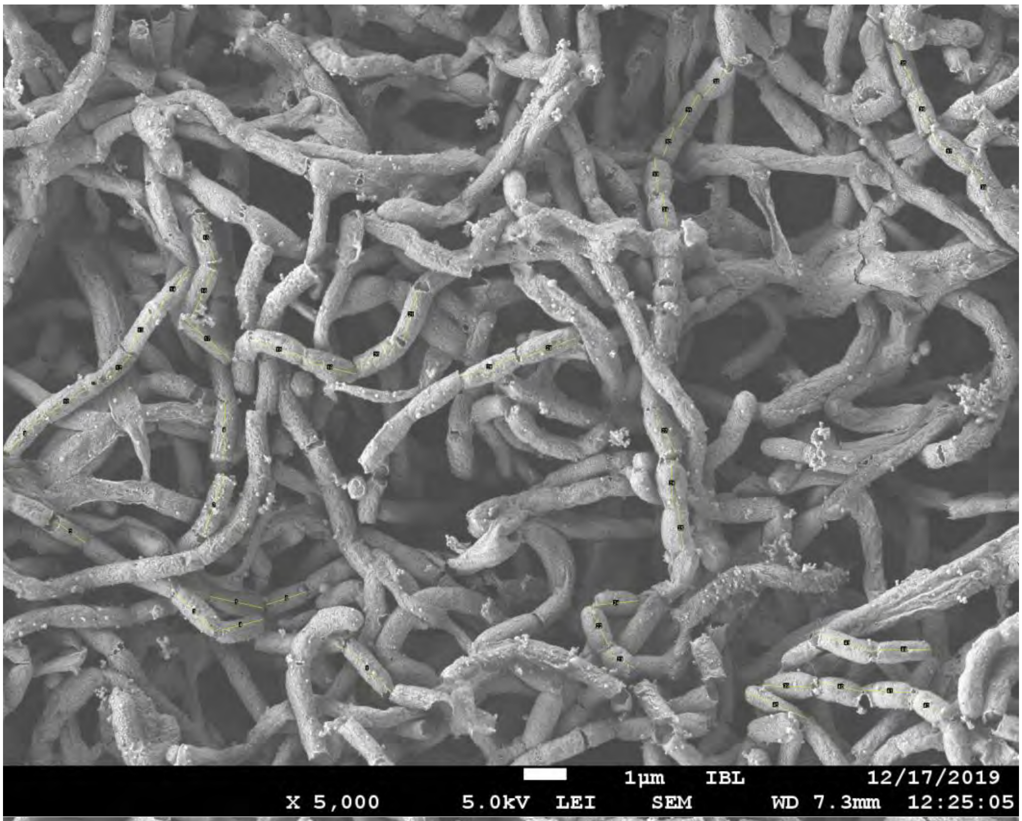

1μm IBL 12/17/2019
X 5,000 5.0kV LEI SEM WD 7.3mm 12:25:05

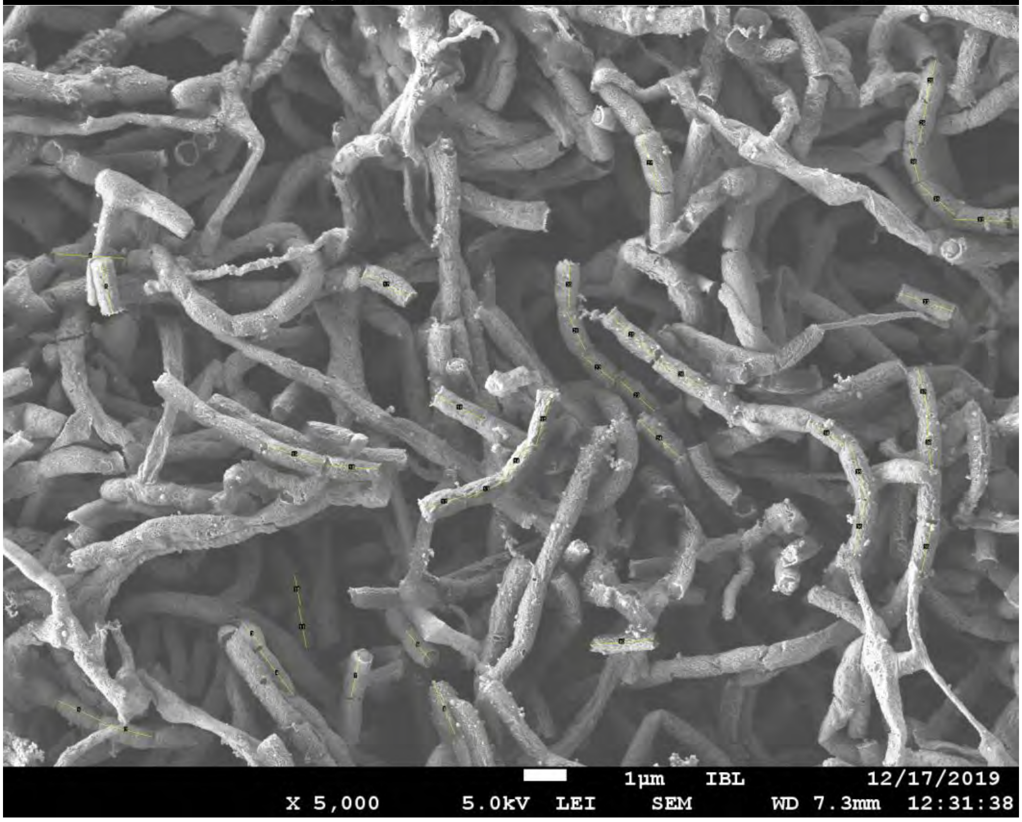

1μm IBL 12/17/2019
X 5,000 5.0kV LEI SEM WD 7.3mm 12:31:38

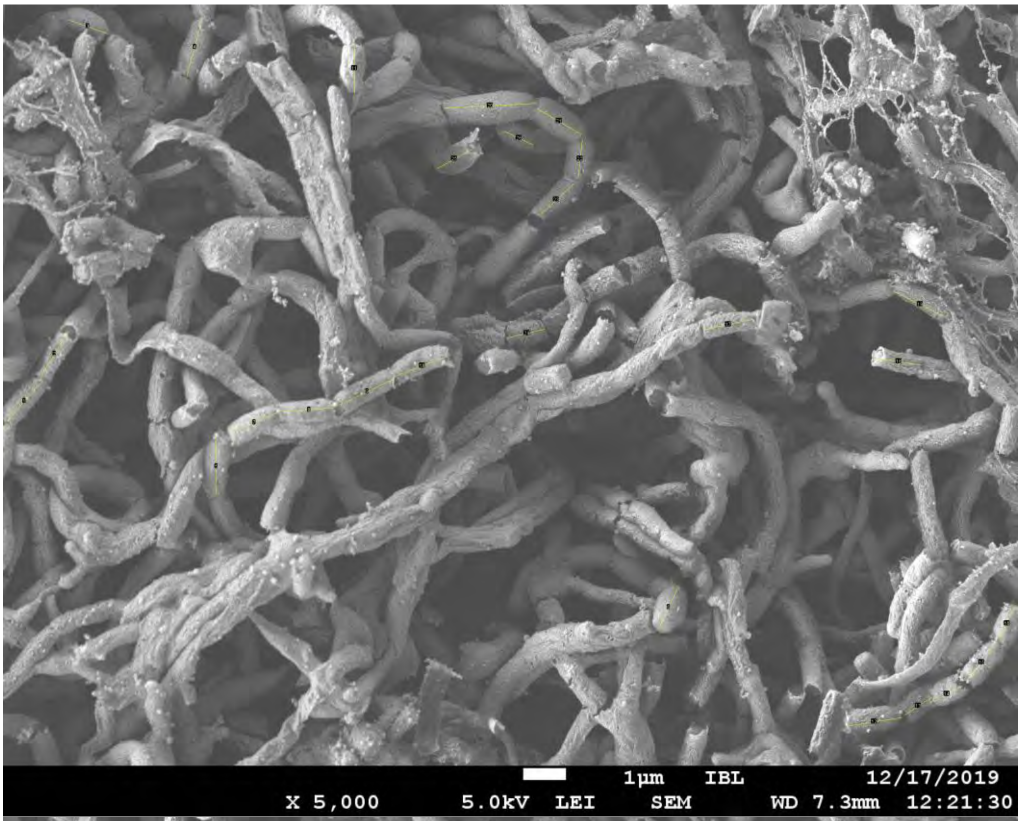
1µm IBL 12/17/2019
X 5,000 5.0kV LEI SEM WD 7.3mm 12:21:30

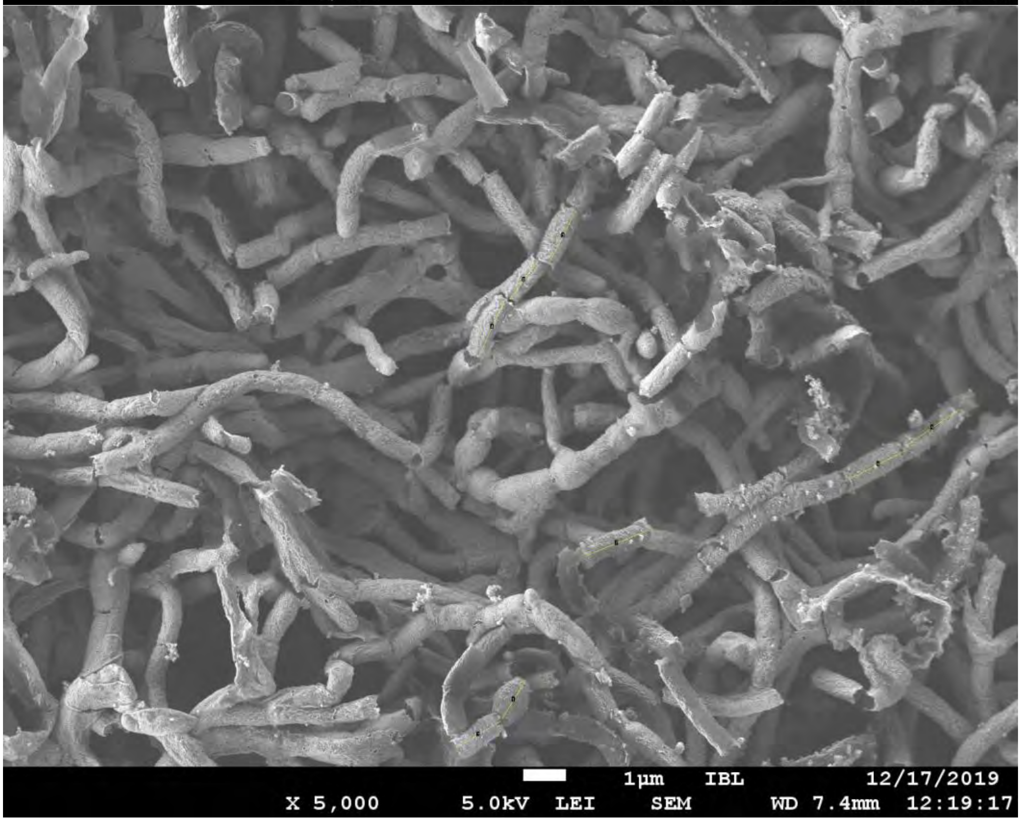
1µm IBL 12/17/2019
X 5,000 5.0kV LEI SEM WD 7.4mm 12:19:17

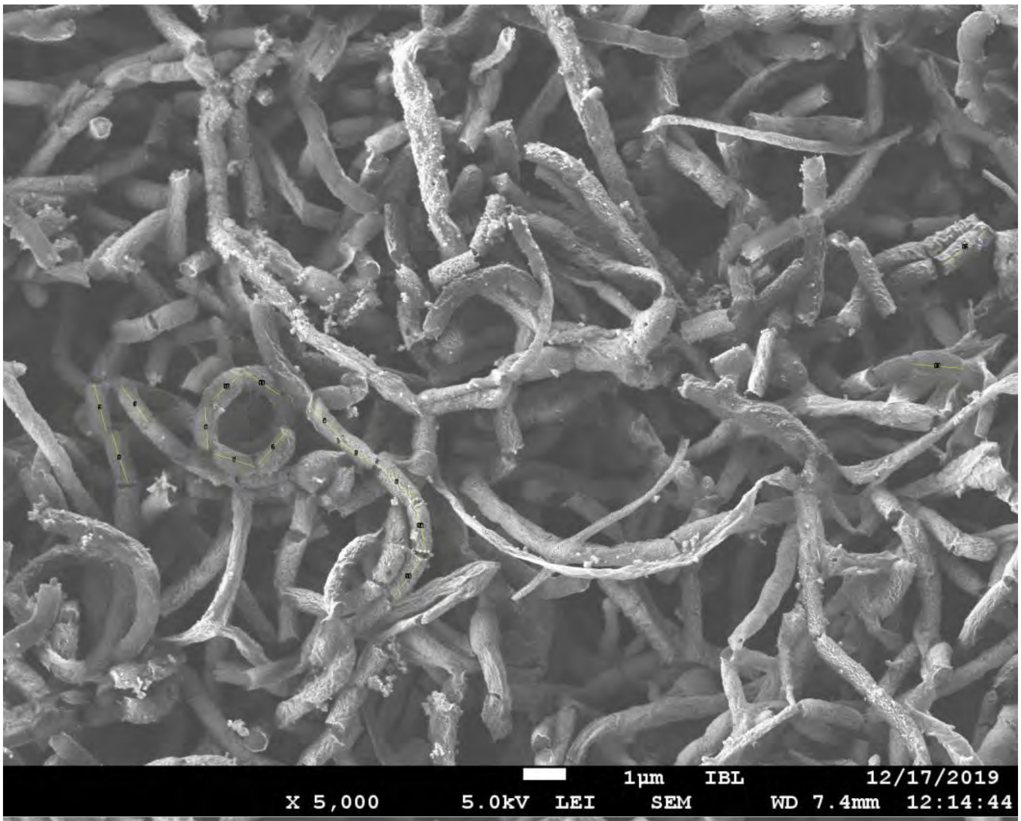
1µm IBL 12/17/2019
X 5,000 5.0kV LEI SEM WD 7.4mm 12:14:44

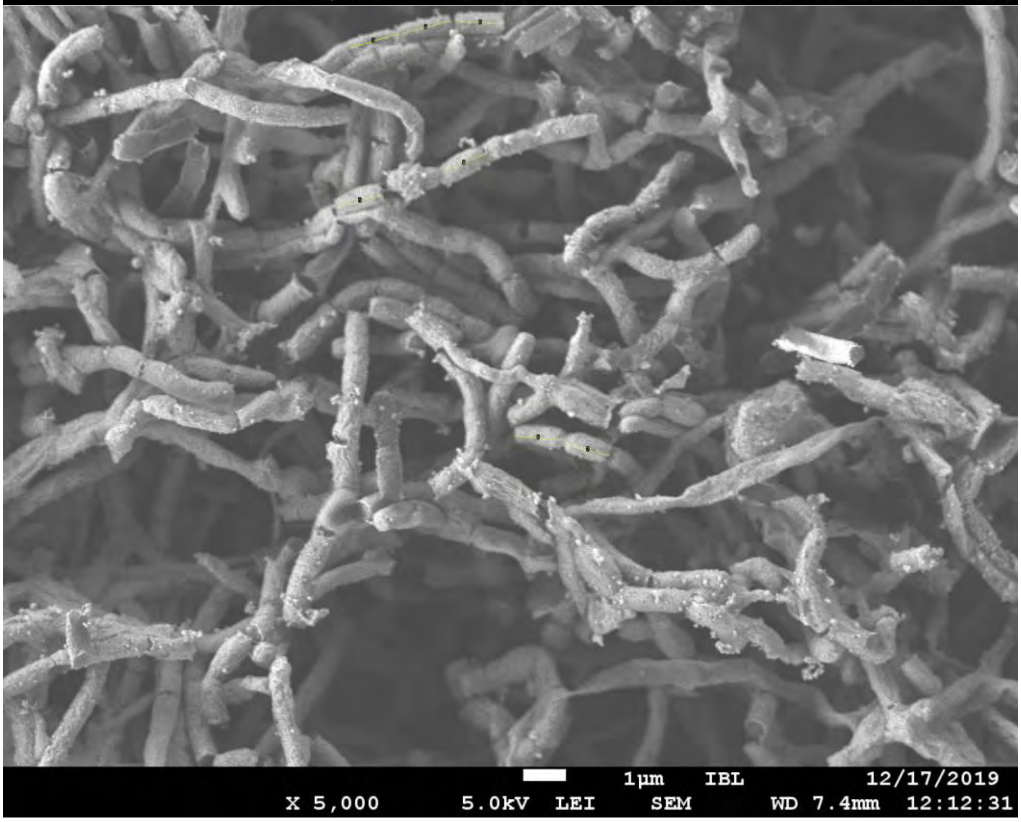
1µm IBL 12/17/2019
X 5,000 5.0kV LEI SEM WD 7.4mm 12:12:31

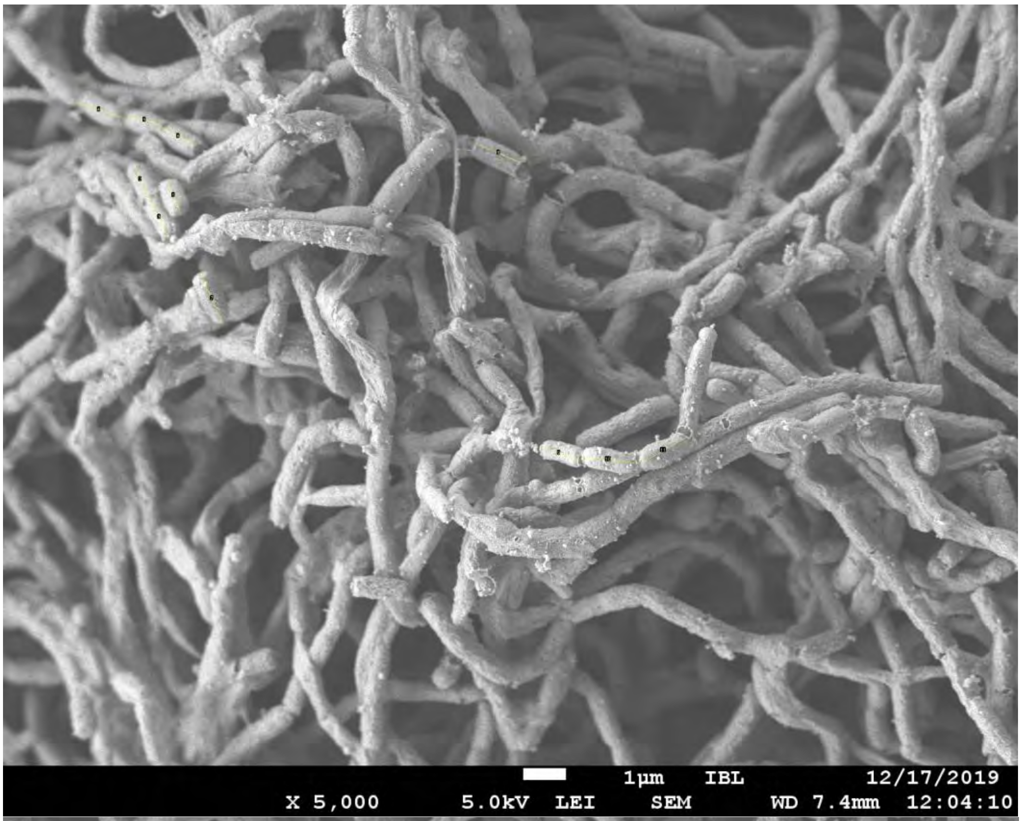
1μm IBL 12/17/2019
X 5,000 5.0kV LEI SEM WD 7.4mm 12:04:10

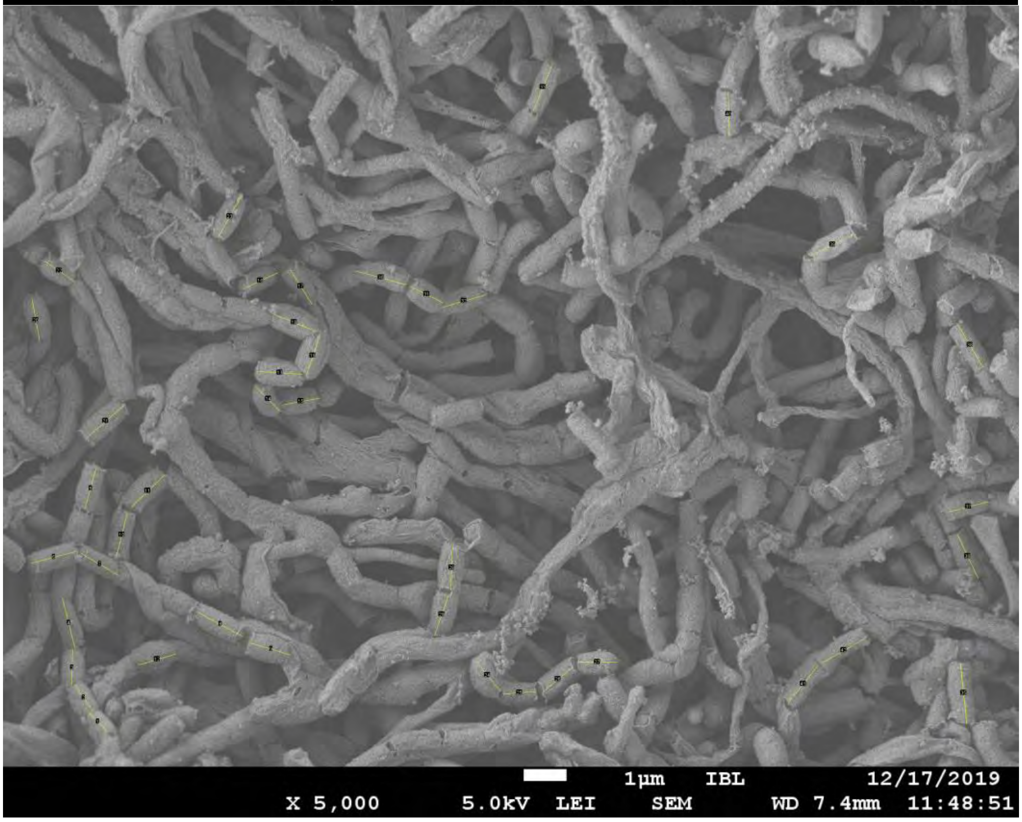
1μm IBL 12/17/2019
X 5,000 5.0kV LEI SEM WD 7.4mm 11:48:51

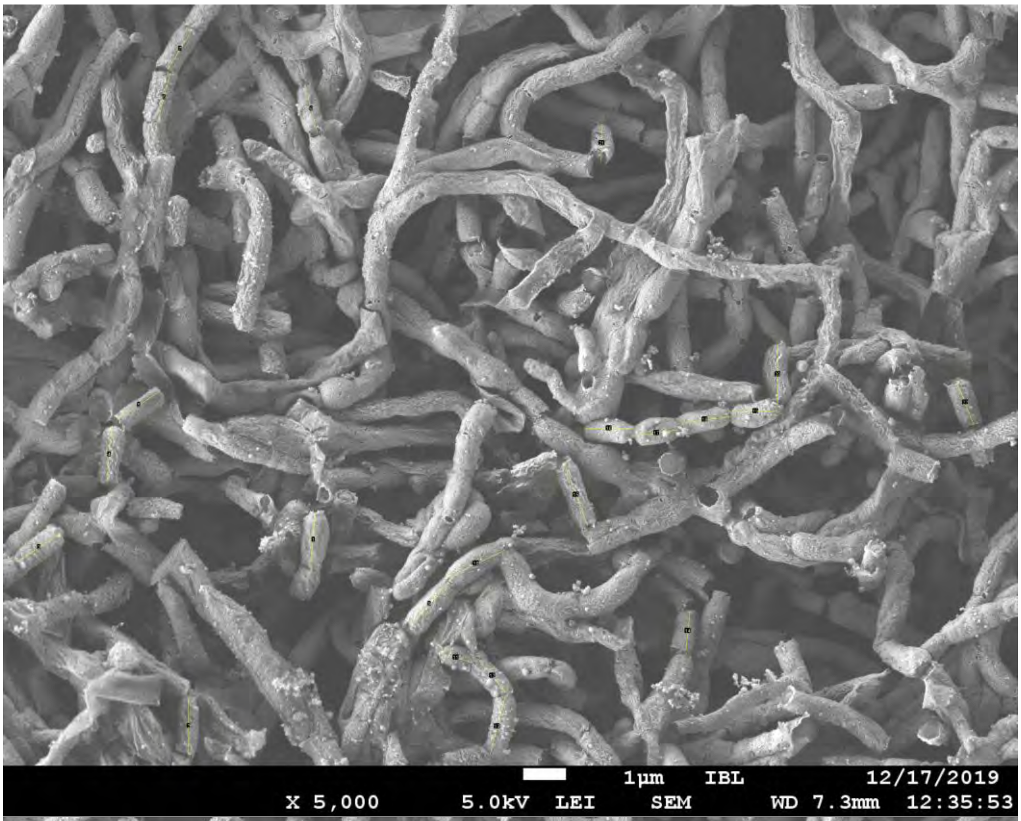
1μm IBL 12/17/2019
X 5,000 5.0kV LEI SEM WD 7.3mm 12:35:53

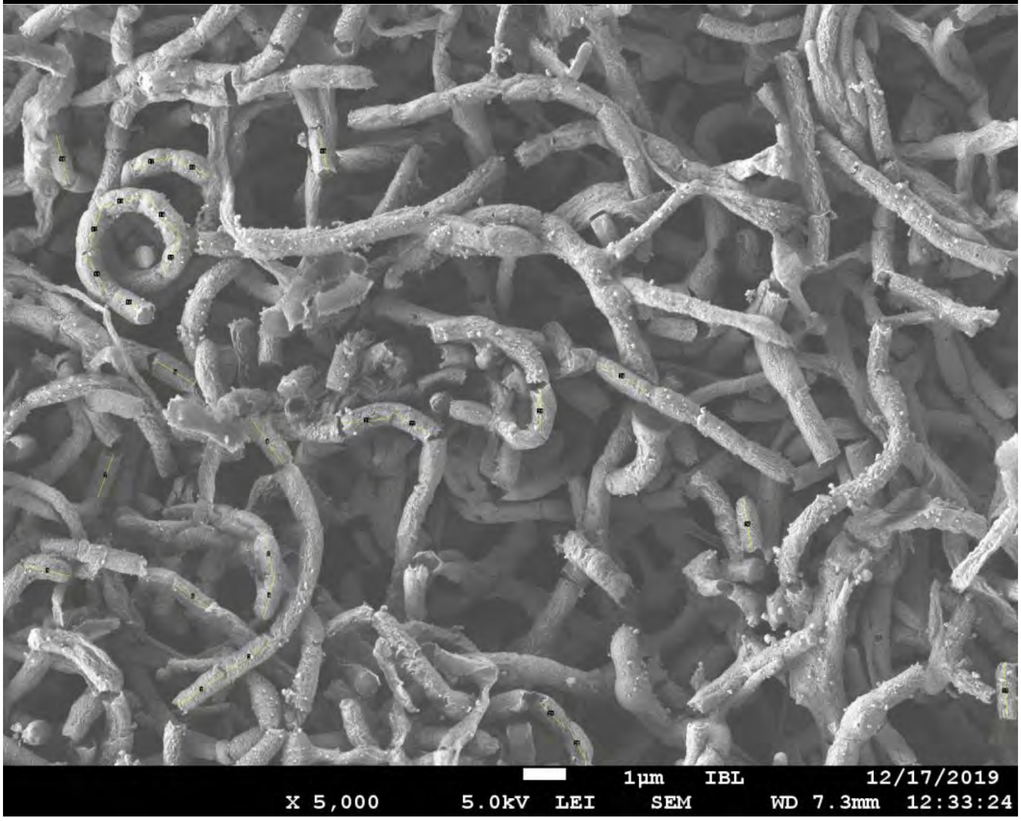
1μm IBL 12/17/2019
X 5,000 5.0kV LEI SEM WD 7.3mm 12:33:24

Mutant 4 (AE)

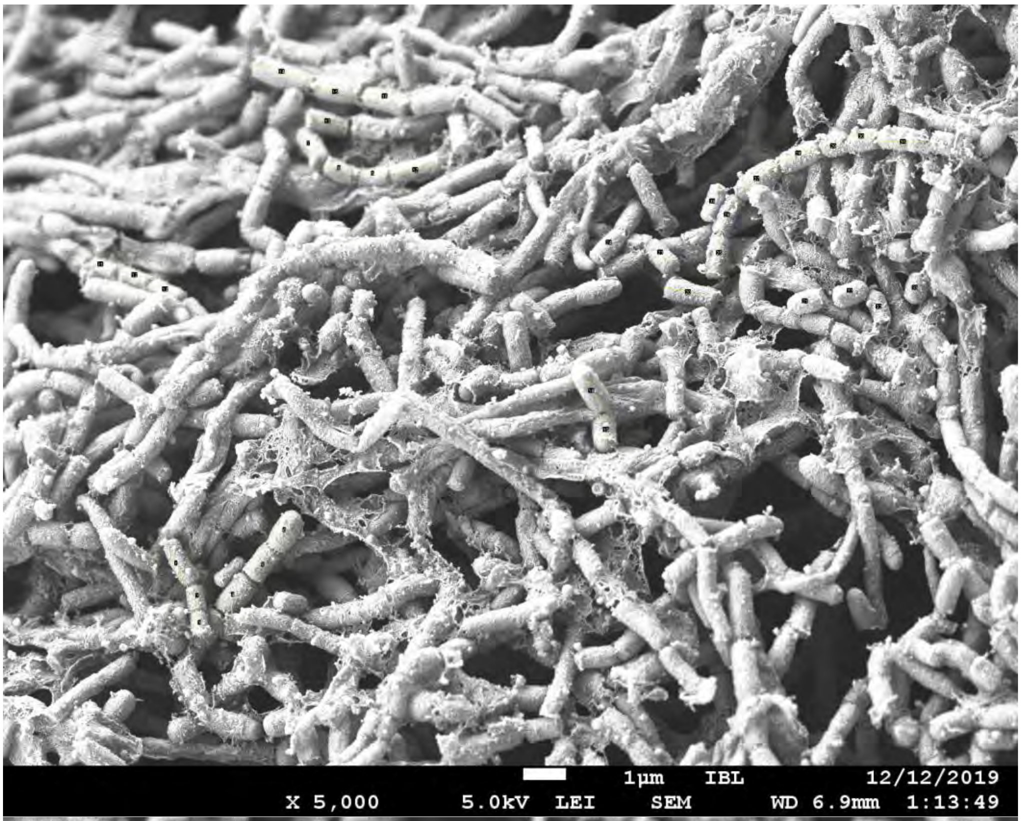

1μm IBL 12/12/2019
X 5,000 5.0kV LEI SEM WD 6.9mm 1:13:49

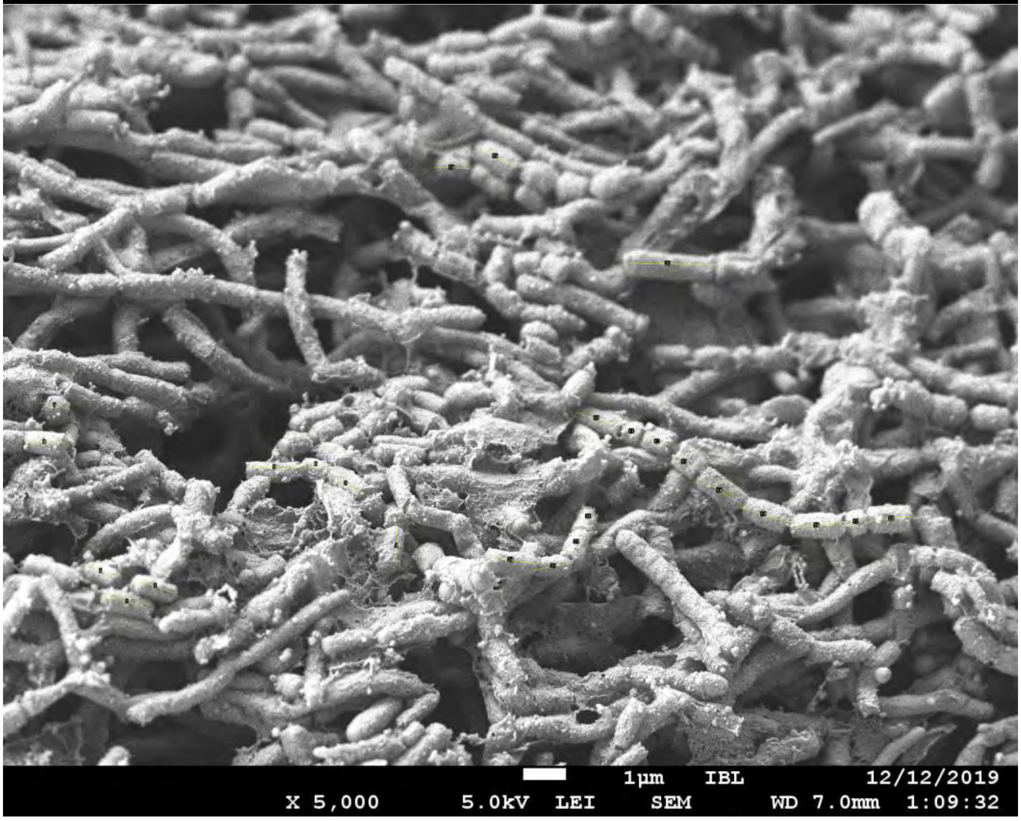

1μm IBL 12/12/2019
X 5,000 5.0kV LEI SEM WD 7.0mm 1:09:32

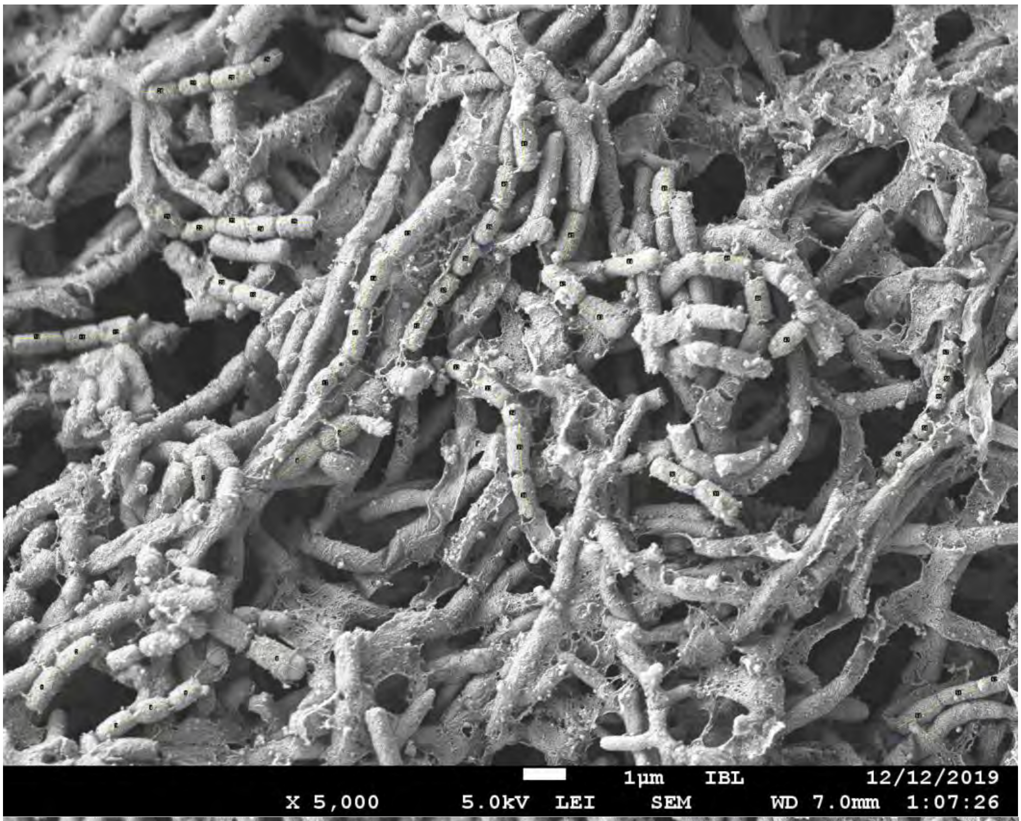
1µm IBL 12/12/2019
X 5,000 5.0kV LEI SEM WD 7.0mm 1:07:26

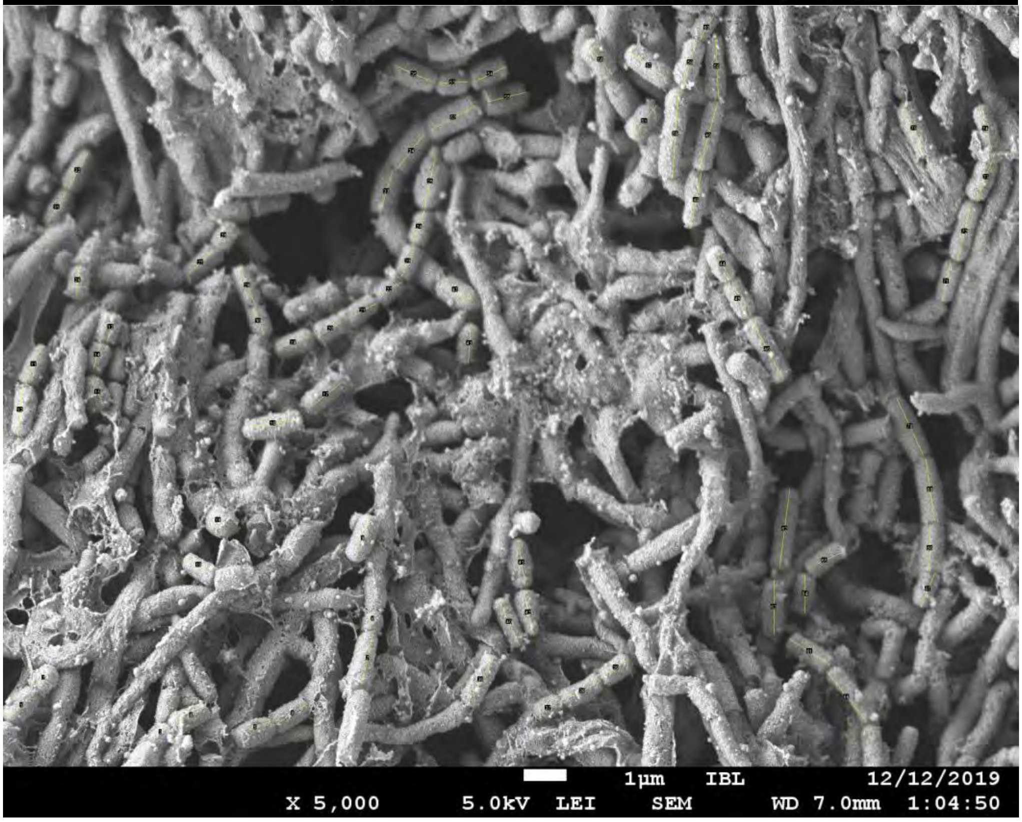
1µm IBL 12/12/2019
X 5,000 5.0kV LEI SEM WD 7.0mm 1:04:50

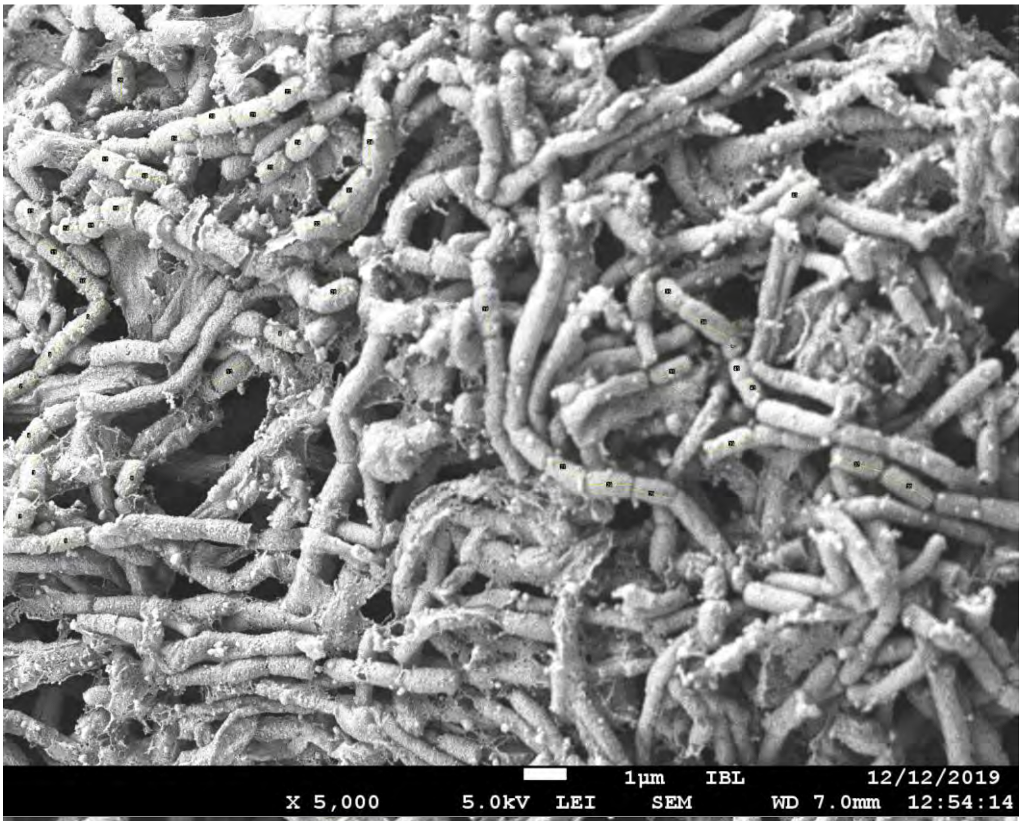
1µm IBL 12/12/2019
X 5,000 5.0kV LEI SEM WD 7.0mm 12:54:14

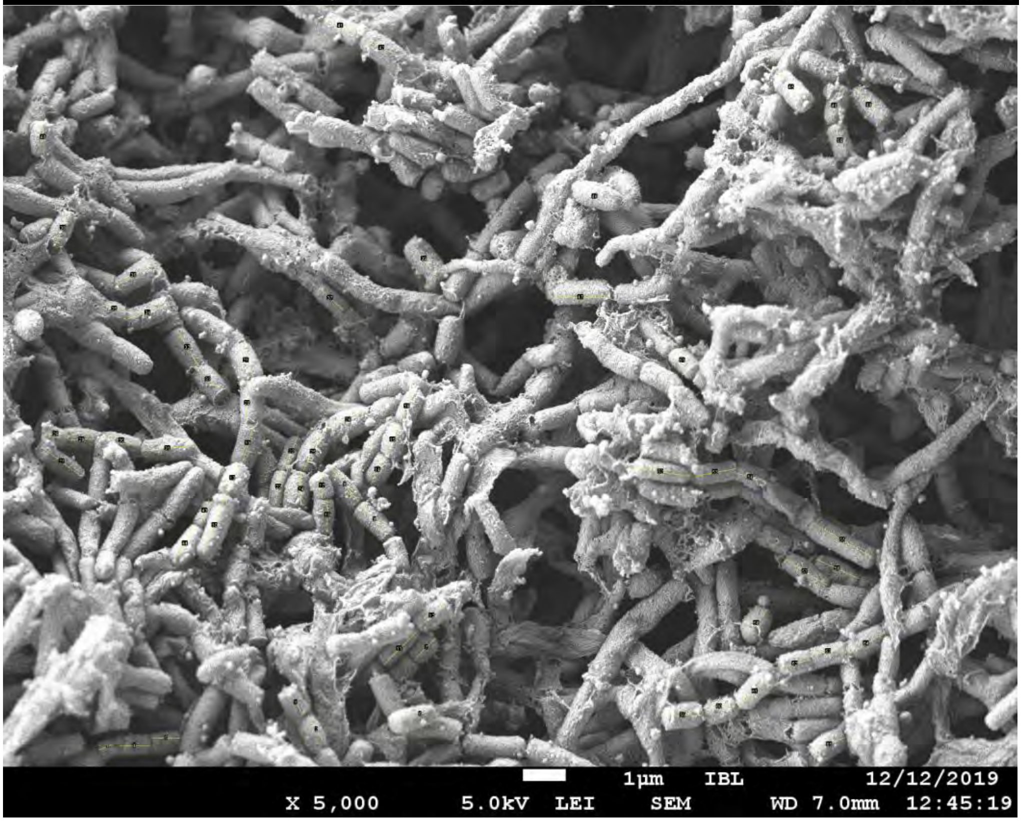
1µm IBL 12/12/2019
X 5,000 5.0kV LEI SEM WD 7.0mm 12:45:19

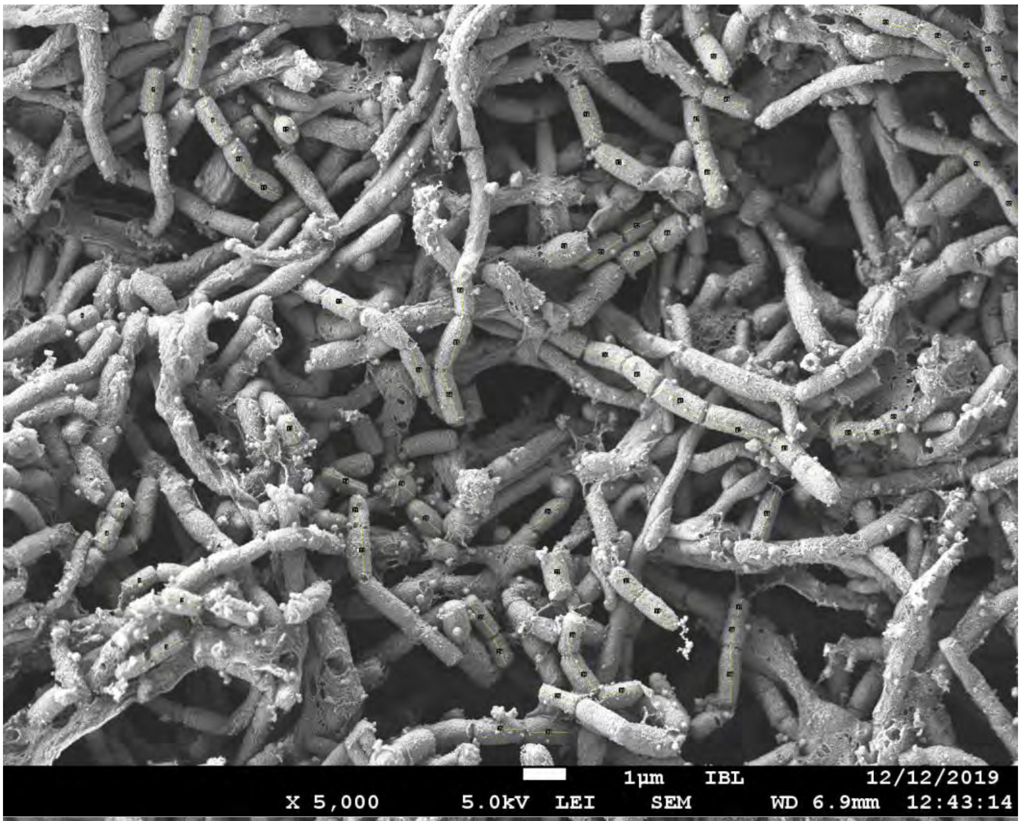
1μm IBL 12/12/2019
X 5,000 5.0kV LEI SEM WD 6.9mm 12:43:14

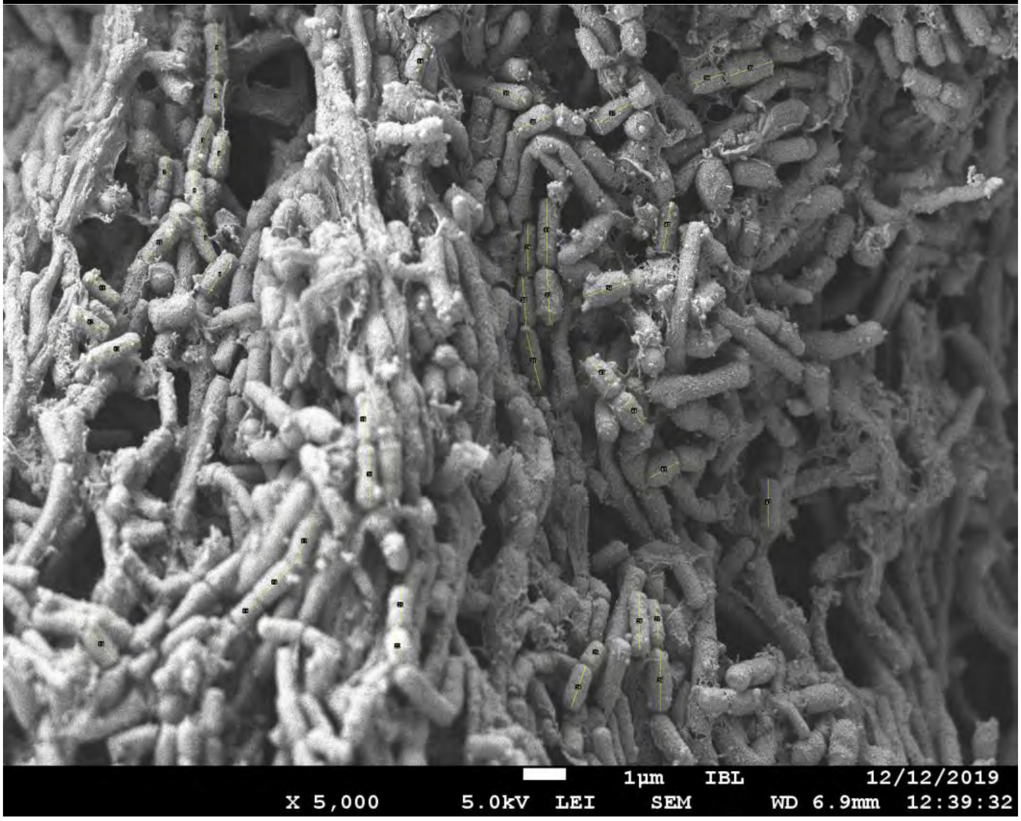
1μm IBL 12/12/2019
X 5,000 5.0kV LEI SEM WD 6.9mm 12:39:32

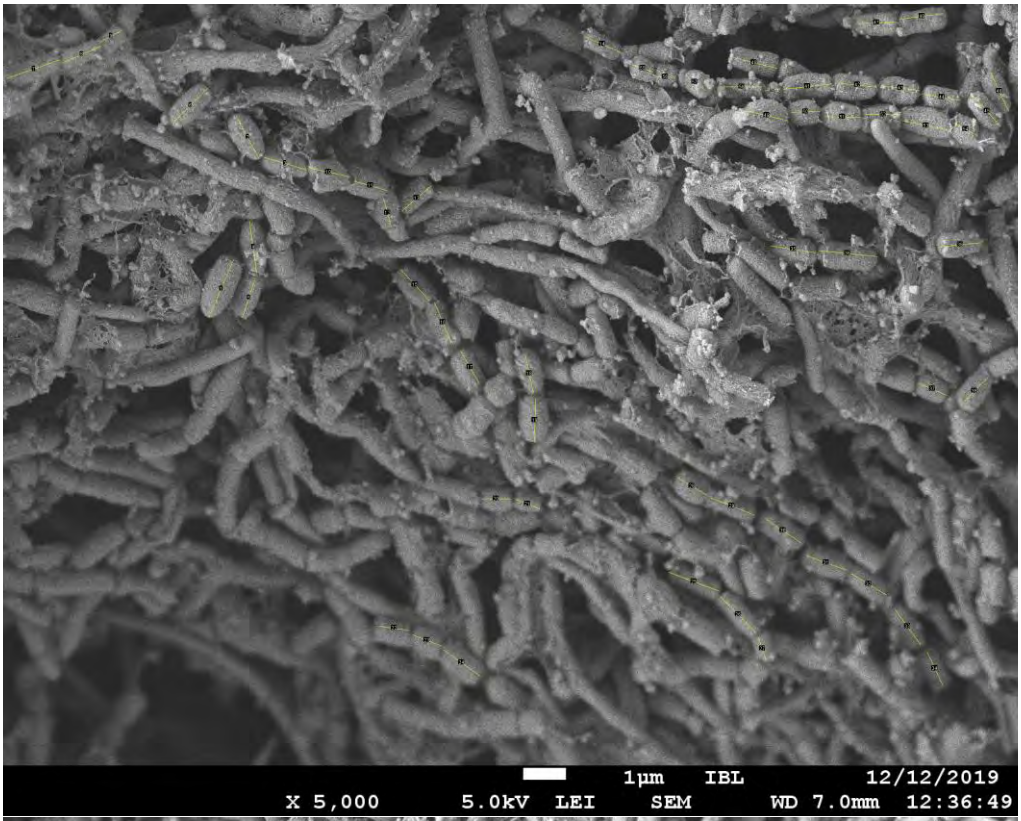
1μm IBL 12/12/2019
X 5,000 5.0kV LEI SEM WD 7.0mm 12:36:49

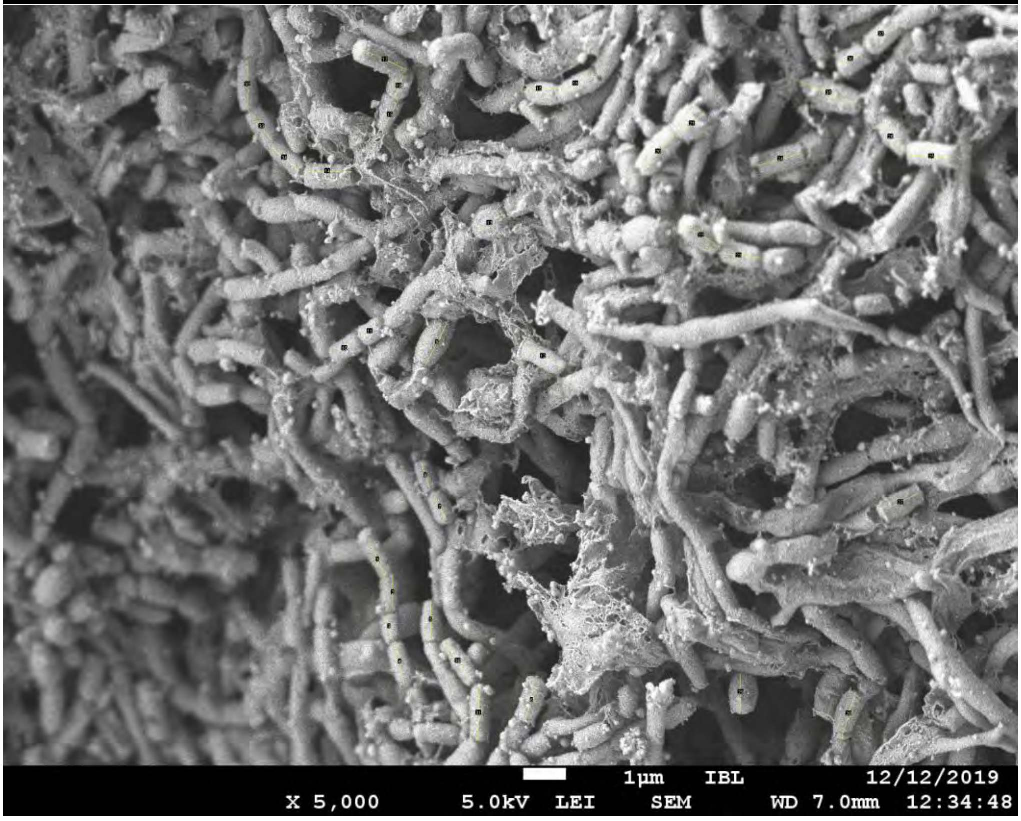
1μm IBL 12/12/2019
X 5,000 5.0kV LEI SEM WD 7.0mm 12:34:48

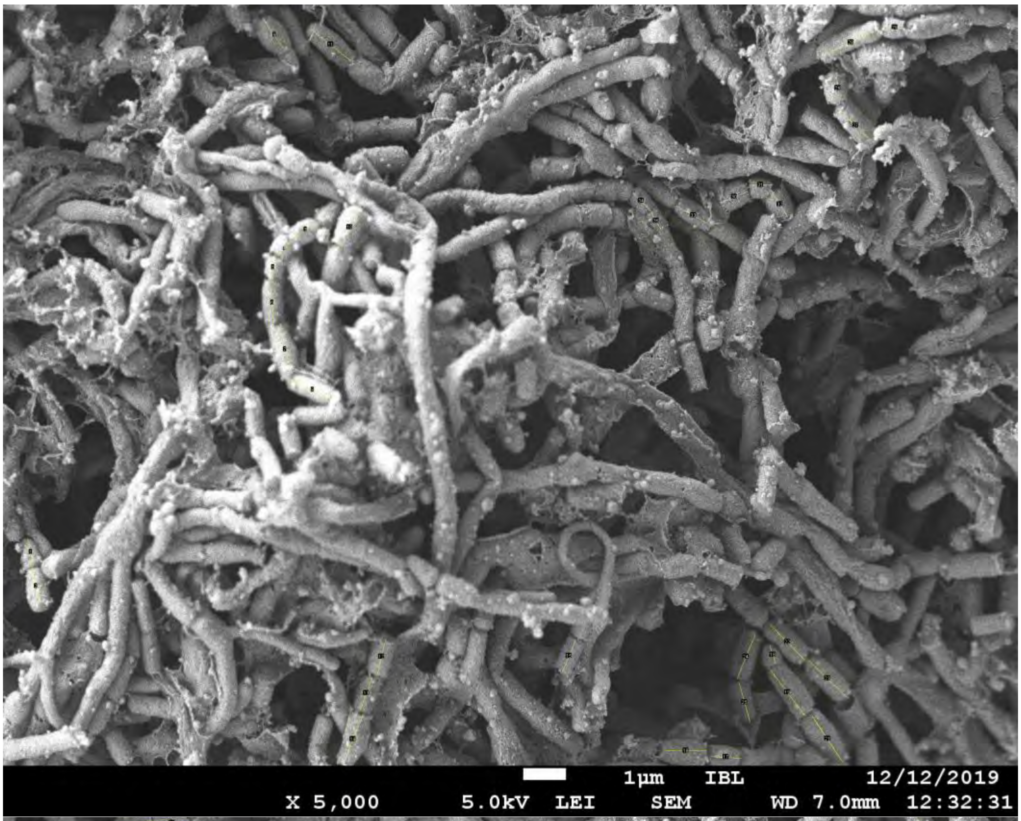
1µm IBL 12/12/2019
X 5,000 5.0kV LEI SEM WD 7.0mm 12:32:31

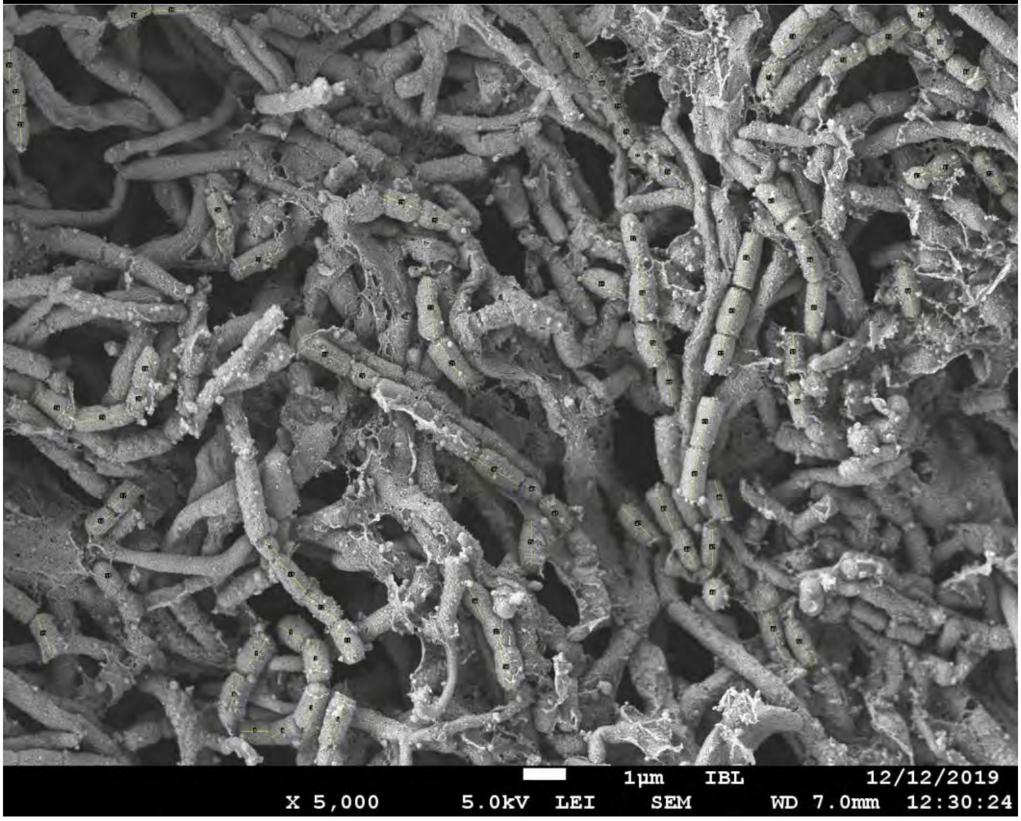
1µm IBL 12/12/2019
X 5,000 5.0kV LEI SEM WD 7.0mm 12:30:24

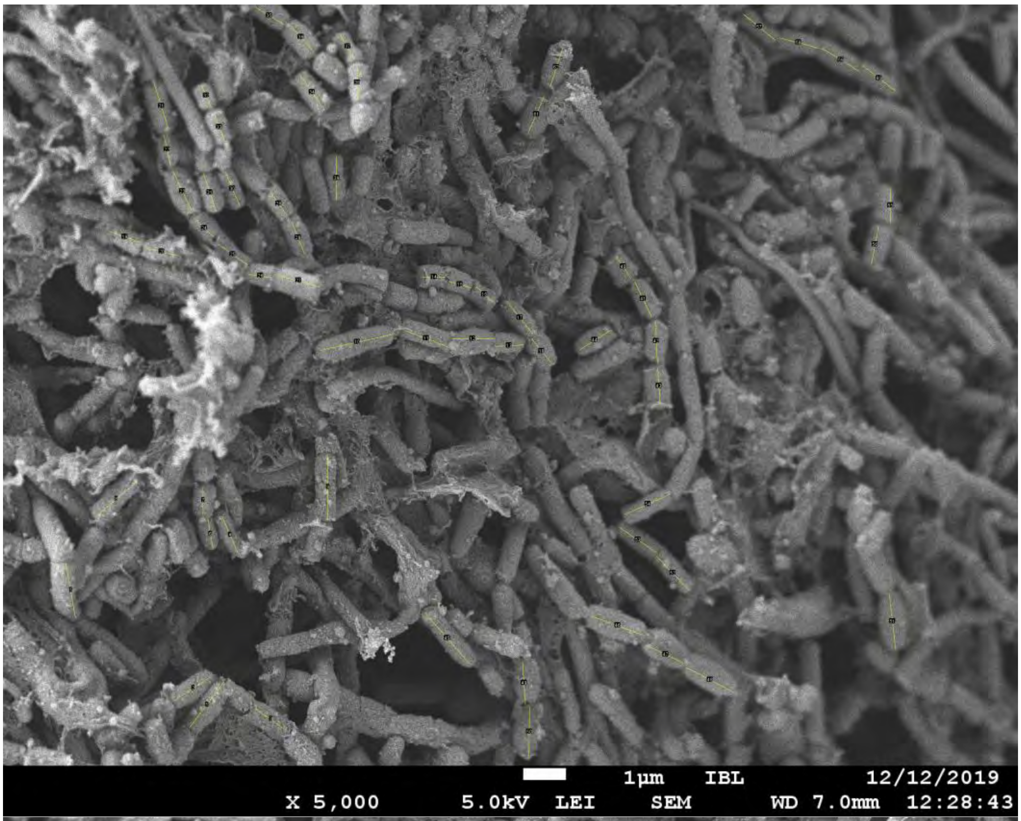
1µm IBL 12/12/2019
X 5,000 5.0kV LEI SEM WD 7.0mm 12:28:43

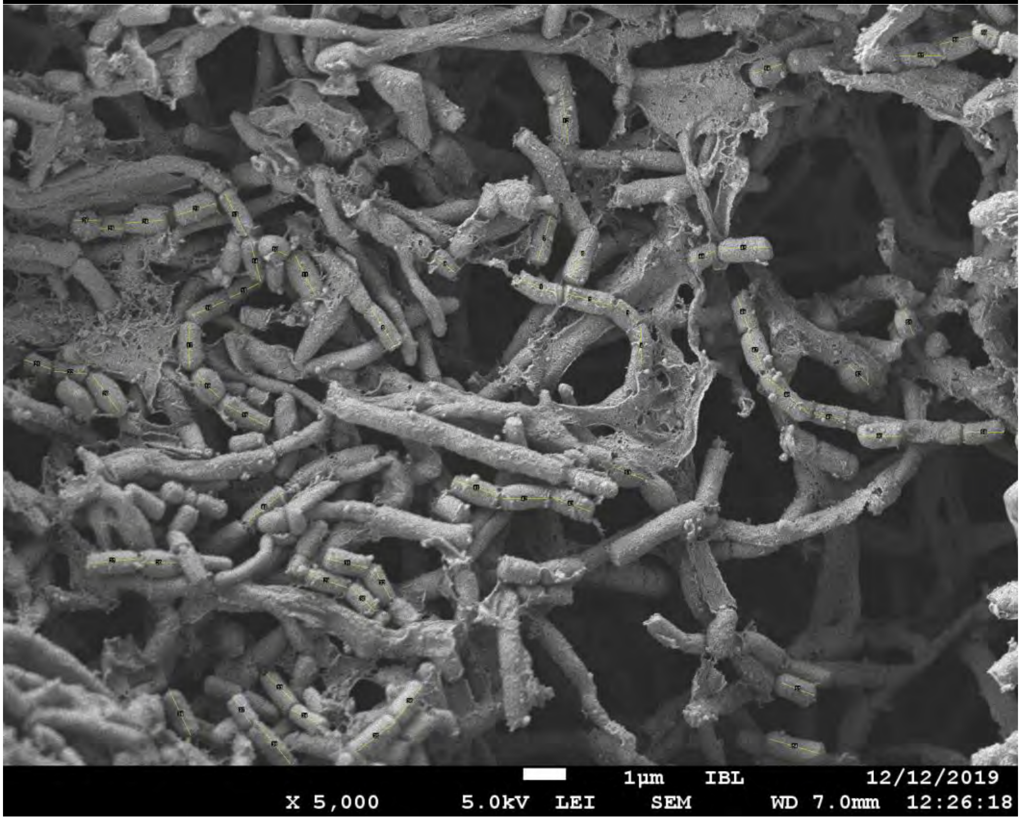
1µm IBL 12/12/2019
X 5,000 5.0kV LEI SEM WD 7.0mm 12:26:18

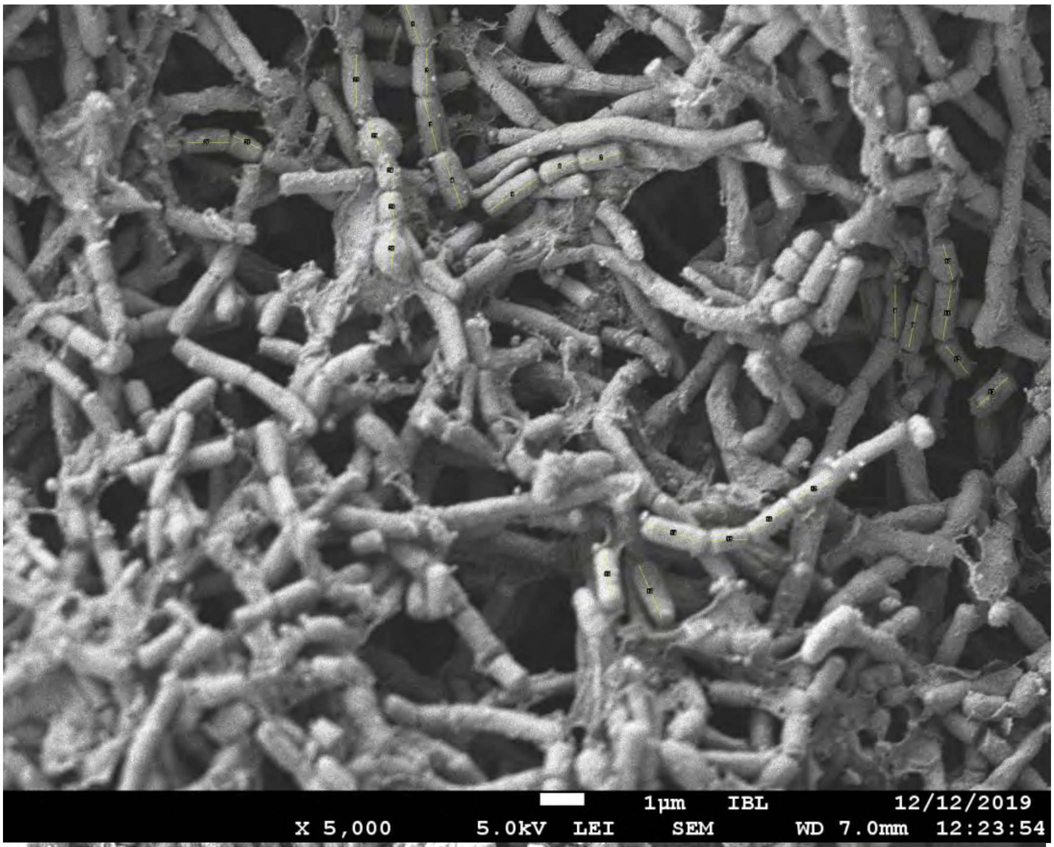
1µm IBL 12/12/2019
X 5,000 5.0kV LEI SEM WD 7.0mm 12:23:54

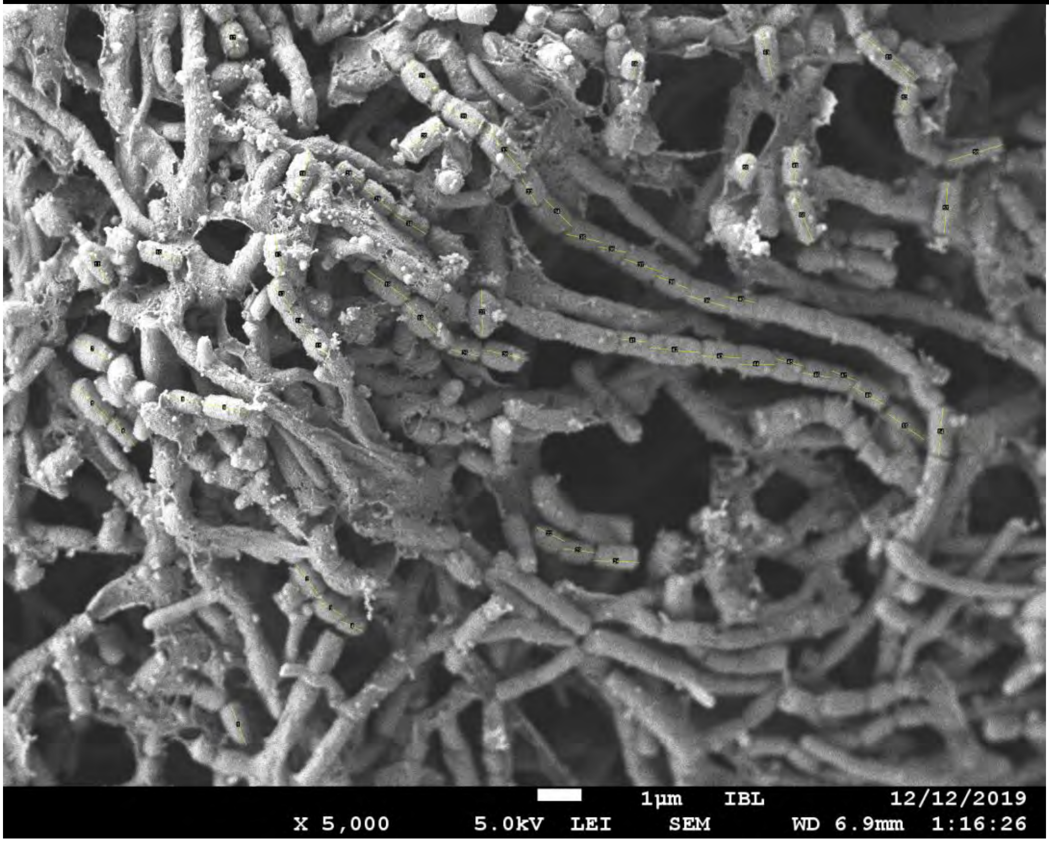
1µm IBL 12/12/2019
X 5,000 5.0kV LEI SEM WD 6.9mm 1:16:26

Mutant 2 (AA)

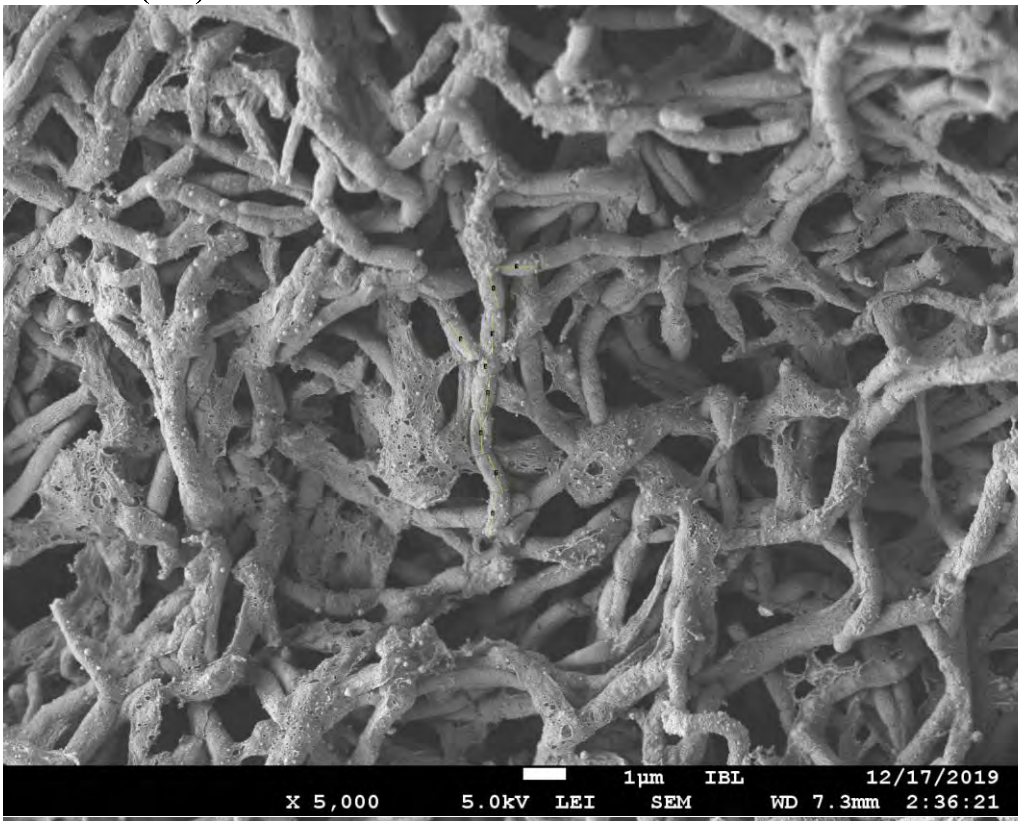
1µm IBL 12/17/2019
X 5,000 5.0kV LEI SEM WD 7.3mm 2:36:21

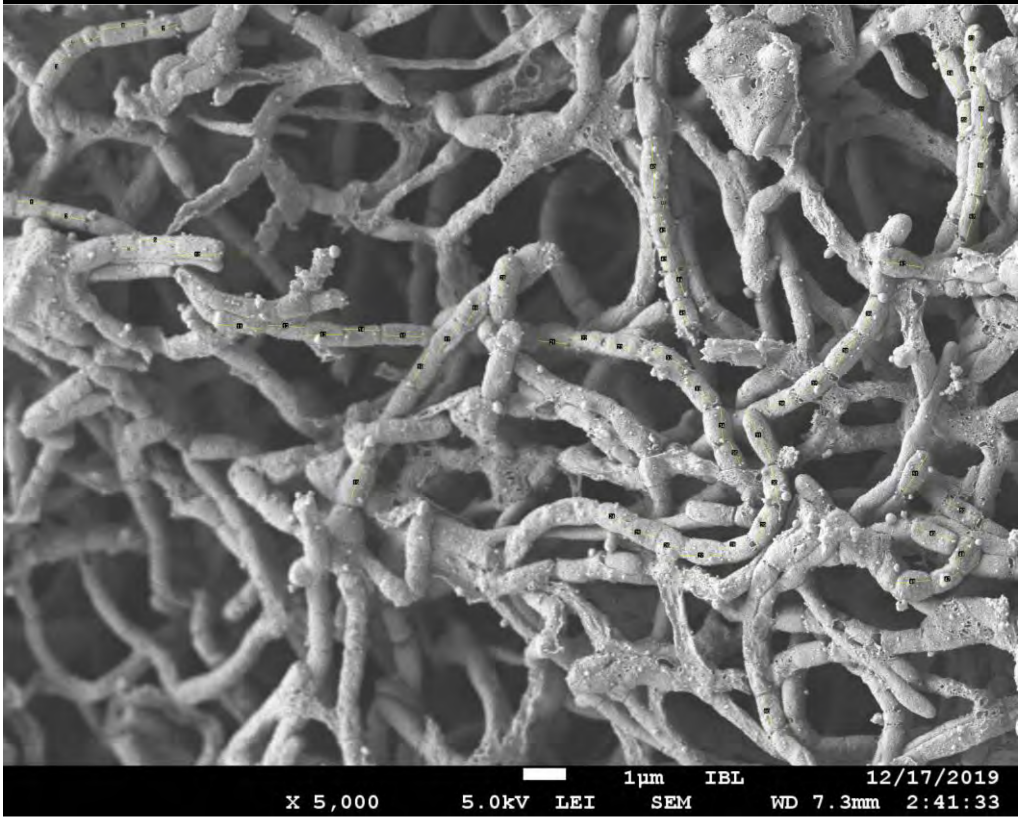
1µm IBL 12/17/2019
X 5,000 5.0kV LEI SEM WD 7.3mm 2:41:33

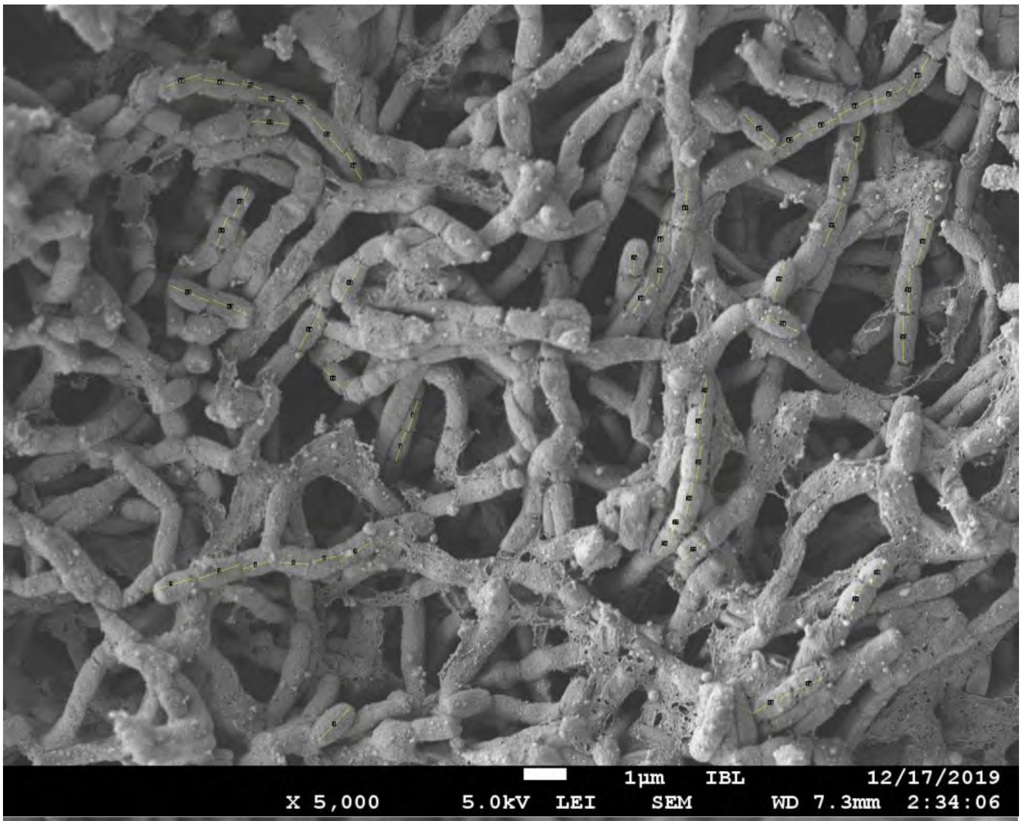
1μm IBL 12/17/2019
X 5,000 5.0kV LEI SEM WD 7.3mm 2:34:06

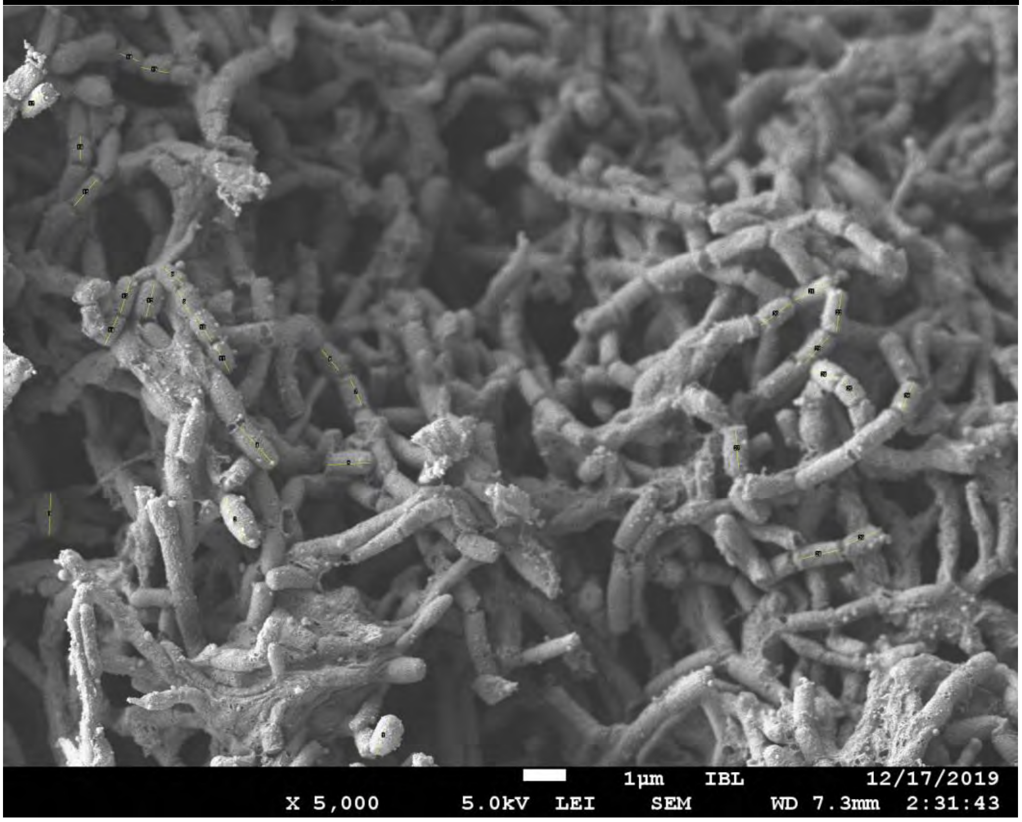
1μm IBL 12/17/2019
X 5,000 5.0kV LEI SEM WD 7.3mm 2:31:43

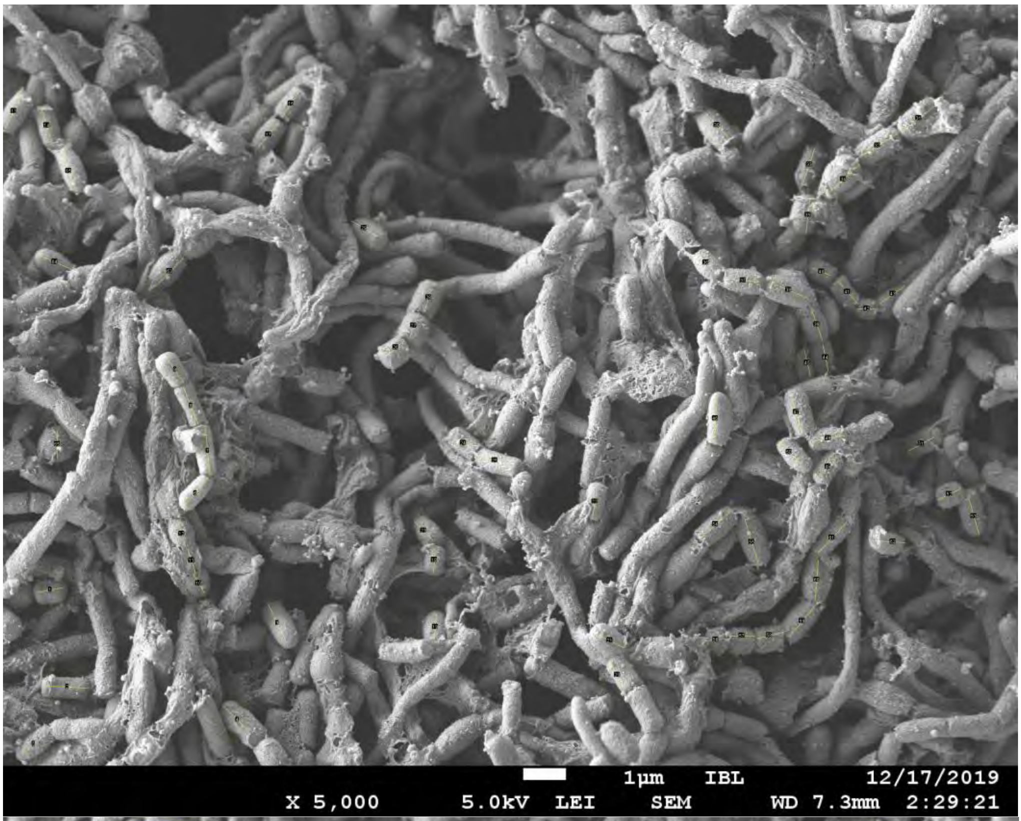
1µm IBL 12/17/2019
X 5,000 5.0kV LEI SEM WD 7.3mm 2:29:21

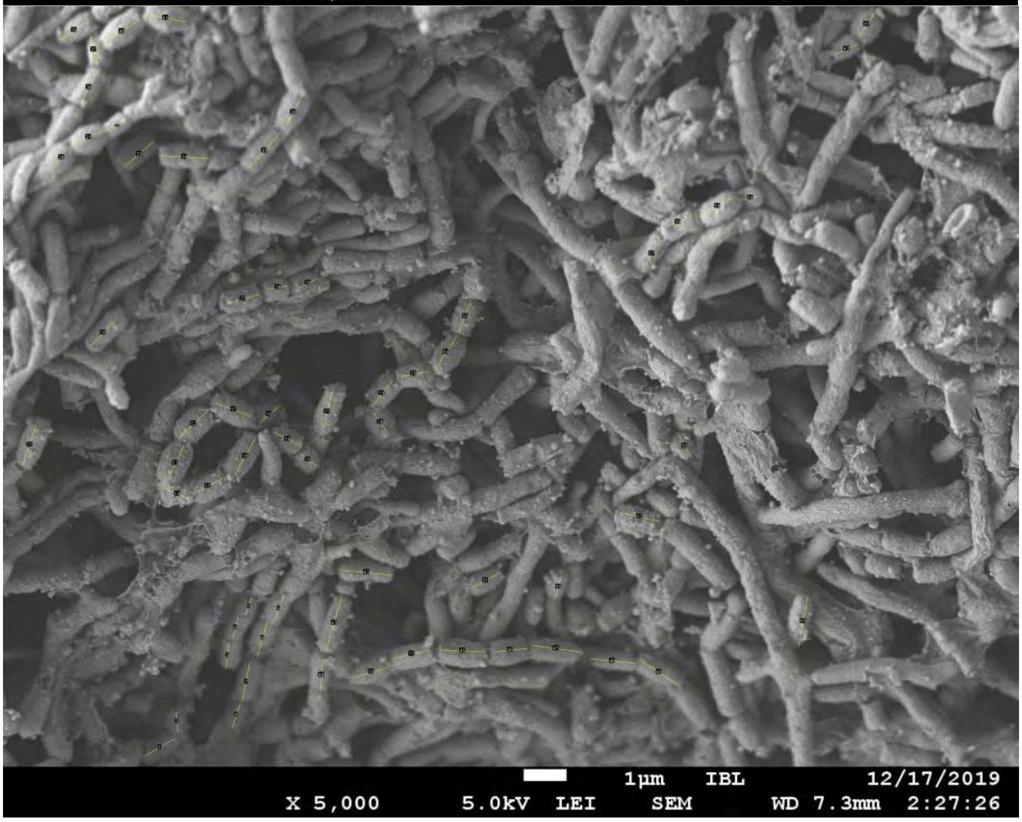
1µm IBL 12/17/2019
X 5,000 5.0kV LEI SEM WD 7.3mm 2:27:26

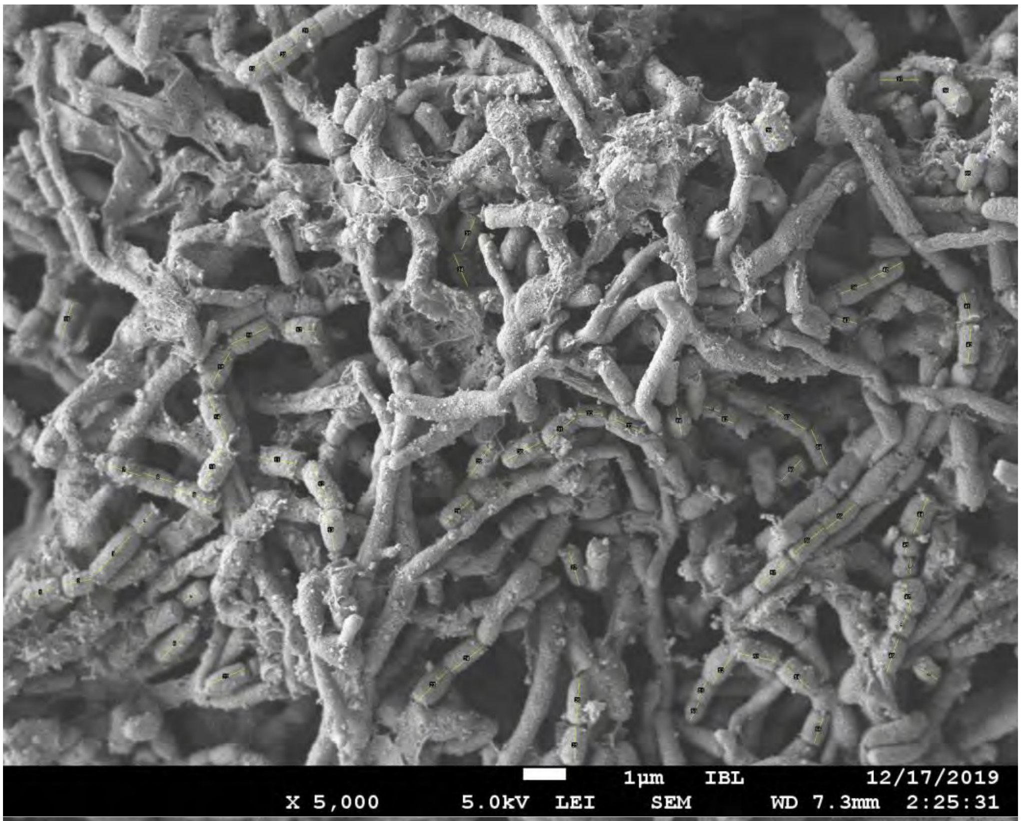
1μm IBL 12/17/2019
X 5,000 5.0kV LEI SEM WD 7.3mm 2:25:31

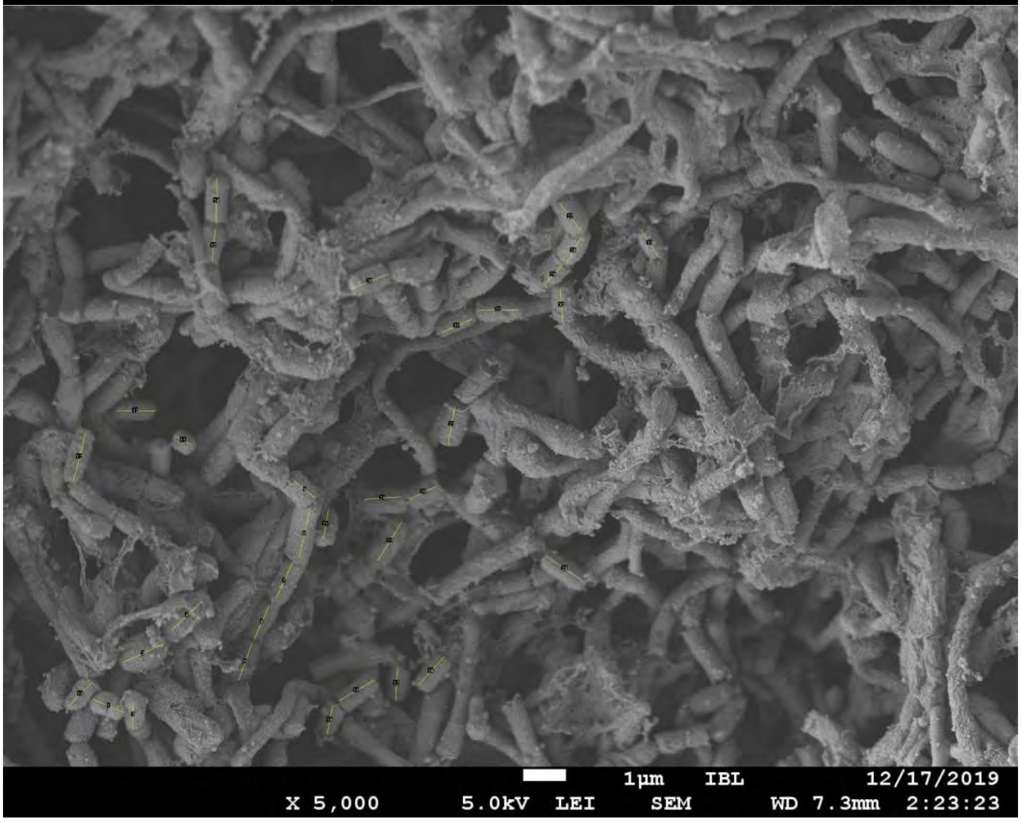
1μm IBL 12/17/2019
X 5,000 5.0kV LEI SEM WD 7.3mm 2:23:23

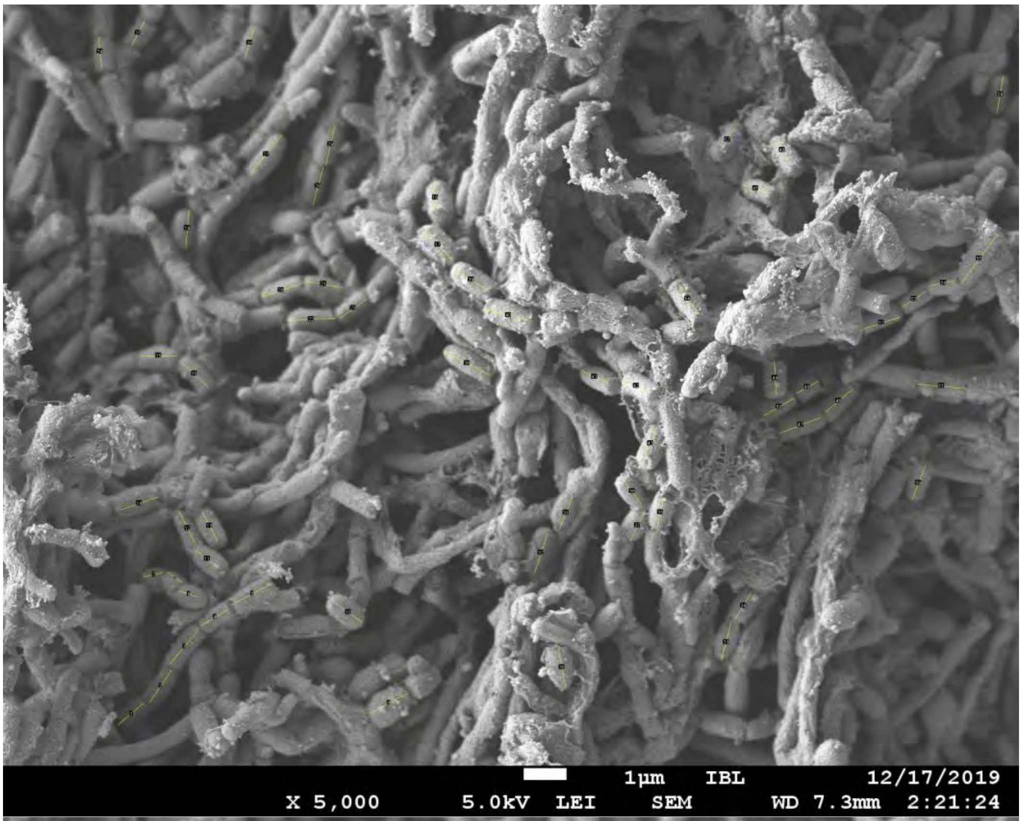
1µm IBL 12/17/2019
X 5,000 5.0kV LEI SEM WD 7.3mm 2:21:24

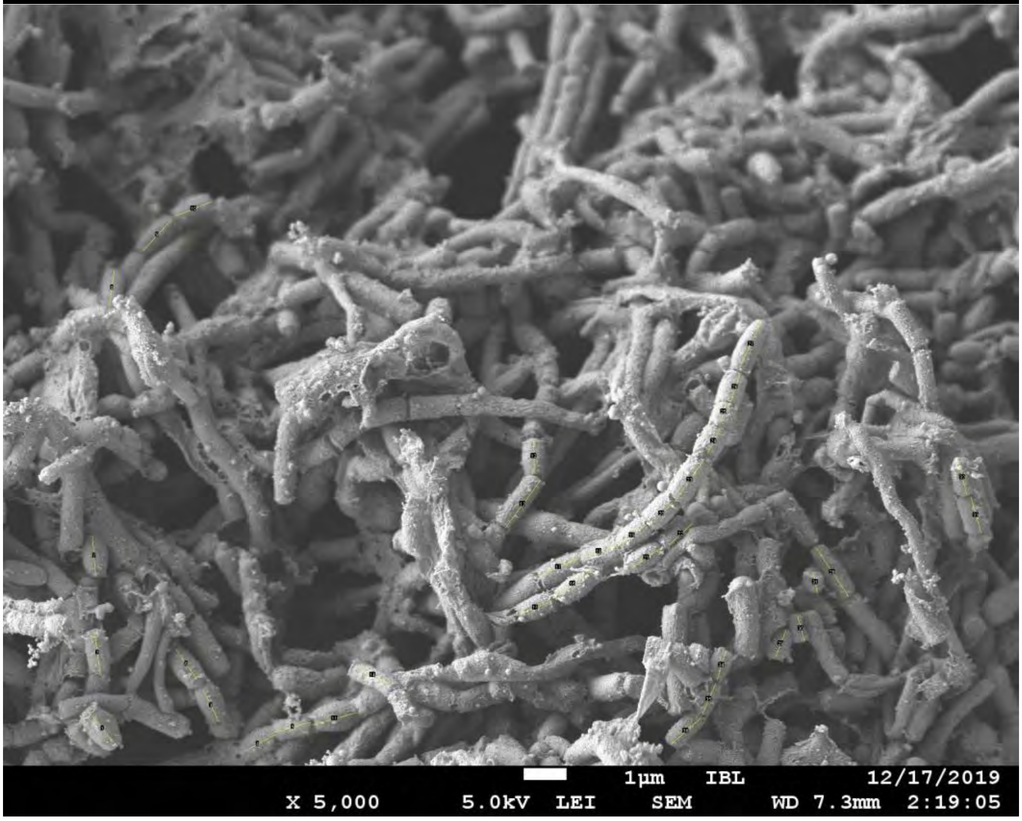
1µm IBL 12/17/2019
X 5,000 5.0kV LEI SEM WD 7.3mm 2:19:05

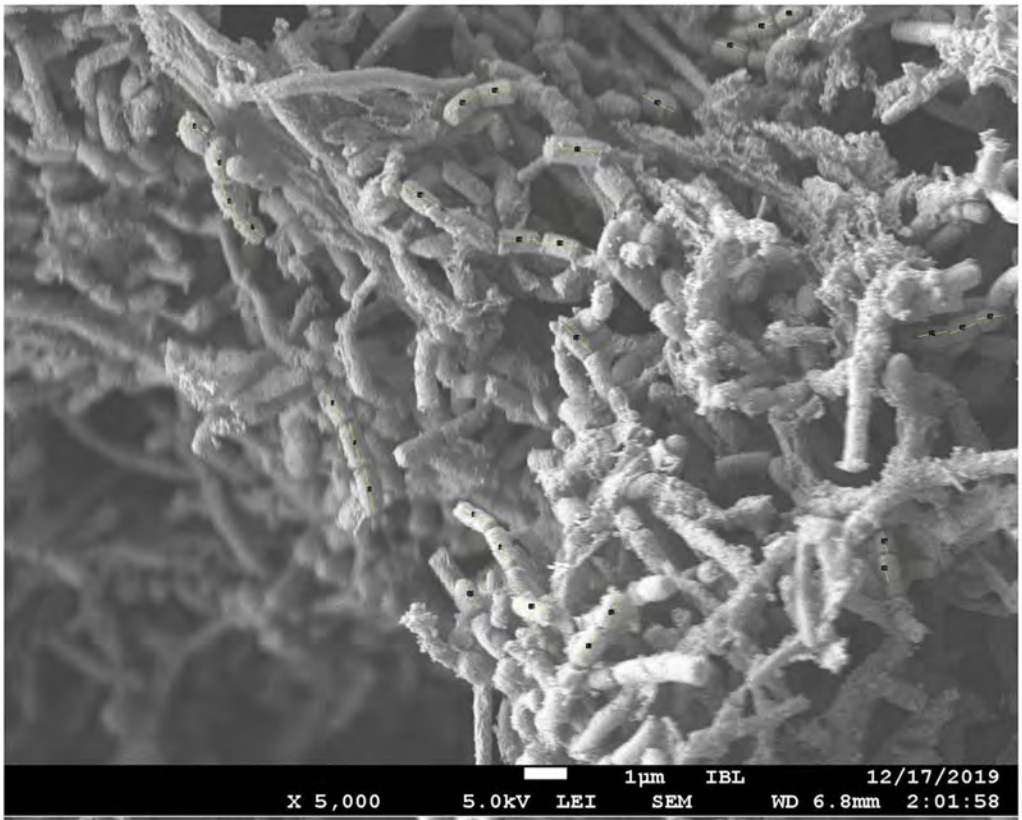
1µm IBL 12/17/2019
X 5,000 5.0kV LEI SEM WD 6.8mm 2:01:58

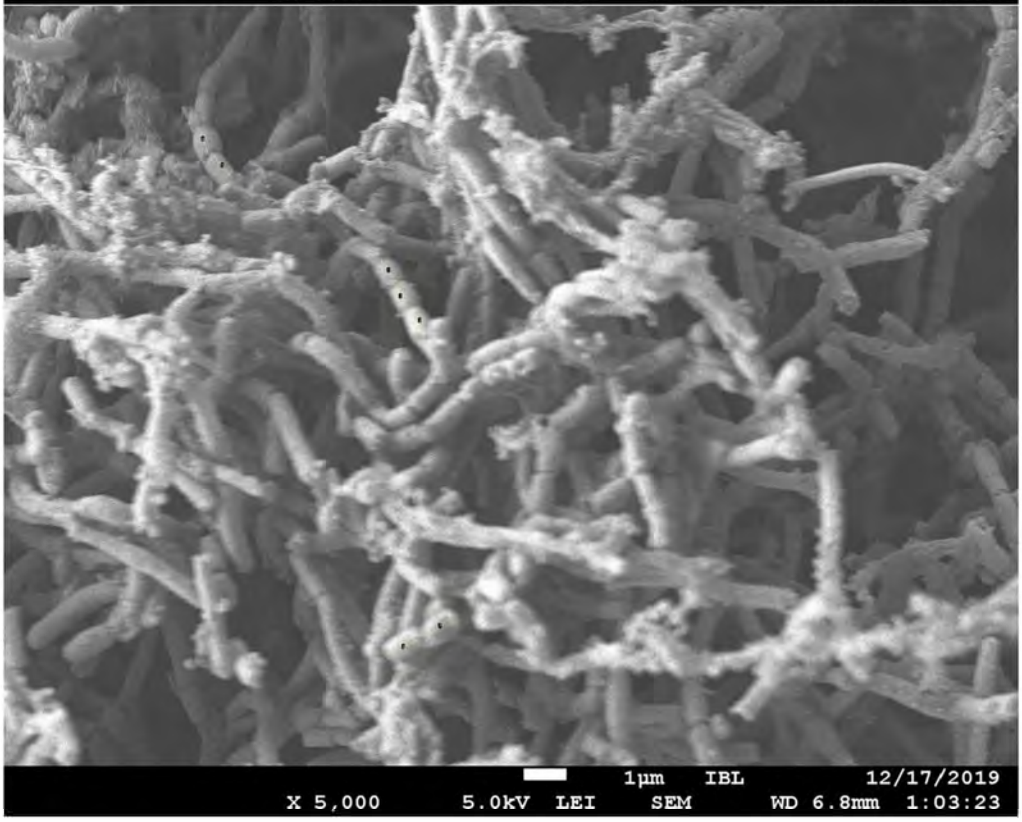
1µm IBL 12/17/2019
X 5,000 5.0kV LEI SEM WD 6.8mm 1:03:23

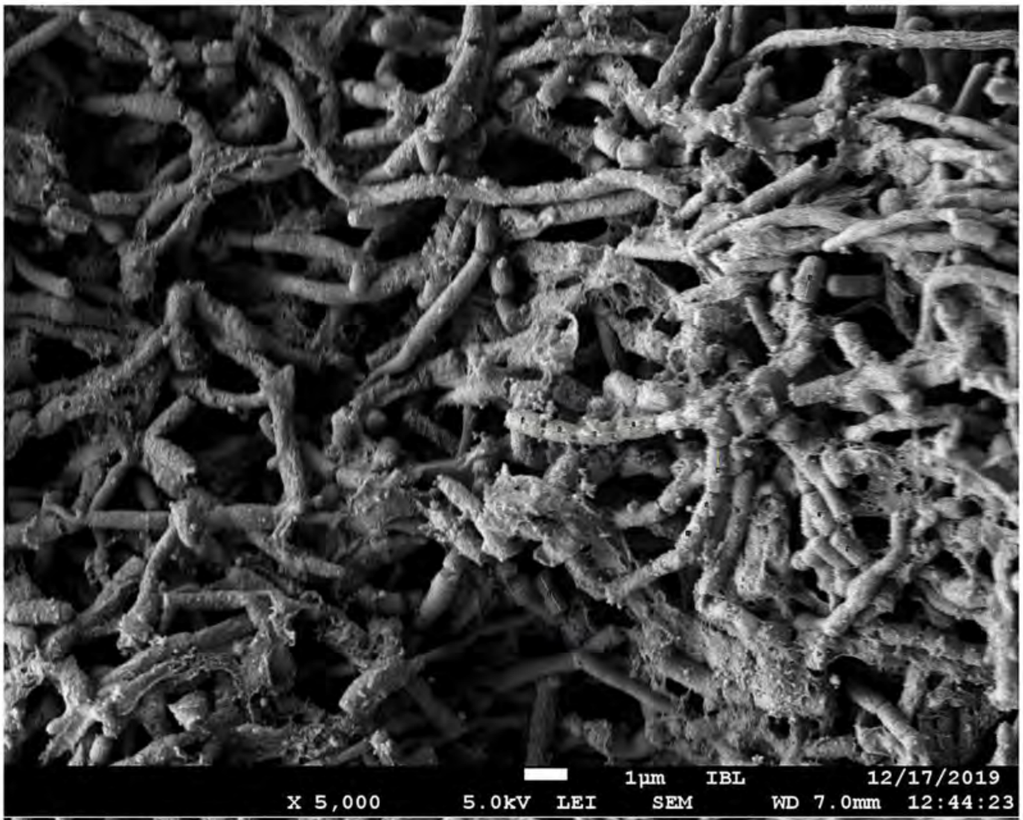
1µm IBL 12/17/2019
X 5,000 5.0kV LEI SEM WD 7.0mm 12:44:23

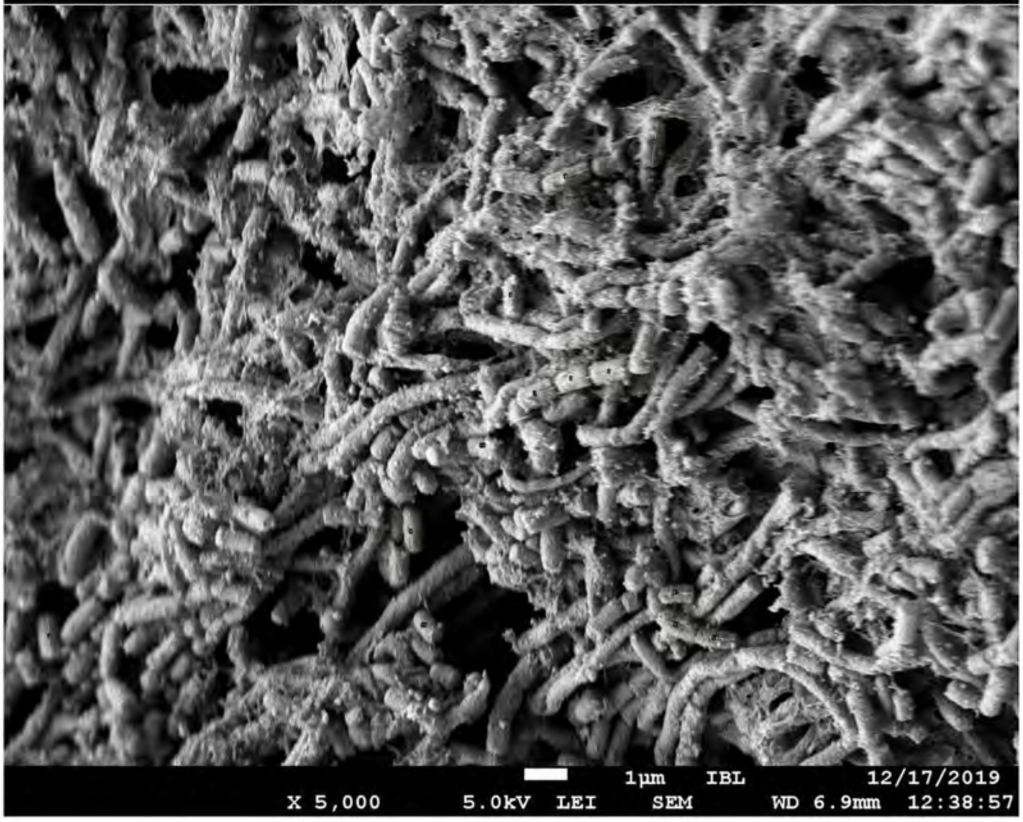
1µm IBL 12/17/2019
X 5,000 5.0kV LEI SEM WD 6.9mm 12:38:57

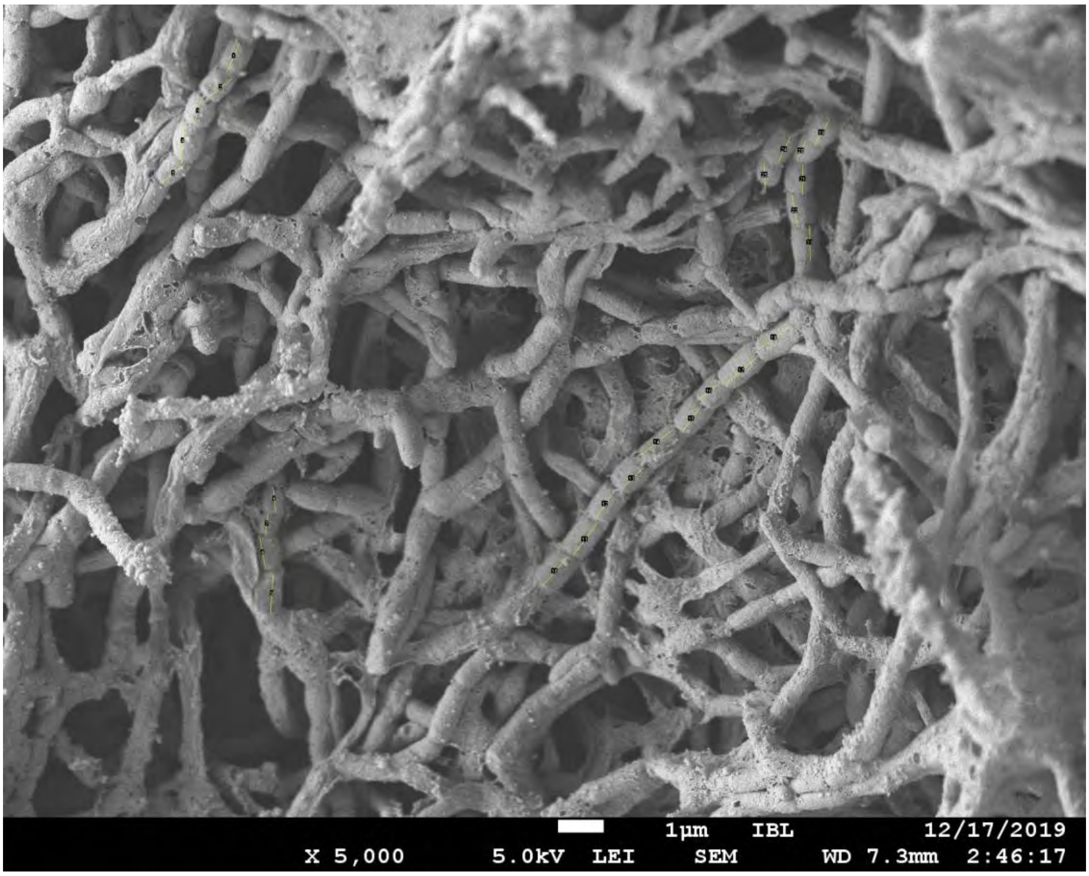
1µm IBL 12/17/2019
X 5,000 5.0kV LEI SEM WD 7.3mm 2:46:17
