# Supplementary material for: FtsZ phosphorylation pleiotropically affects Z-ladder formation, antibiotic production, and morphogenesis in Streptomyces coelicolor
Source: Antonie Van Leeuwenhoek. 2022 Nov 16;116(1):1–19. doi: 10.1007/s10482-022-01778-w (PMC9823044; doi:10.1007/s10482-022-01778-w)

Supplementary figure 3. A) Uncropped SEM images of mutant singularities shown in Figure 4.

WT

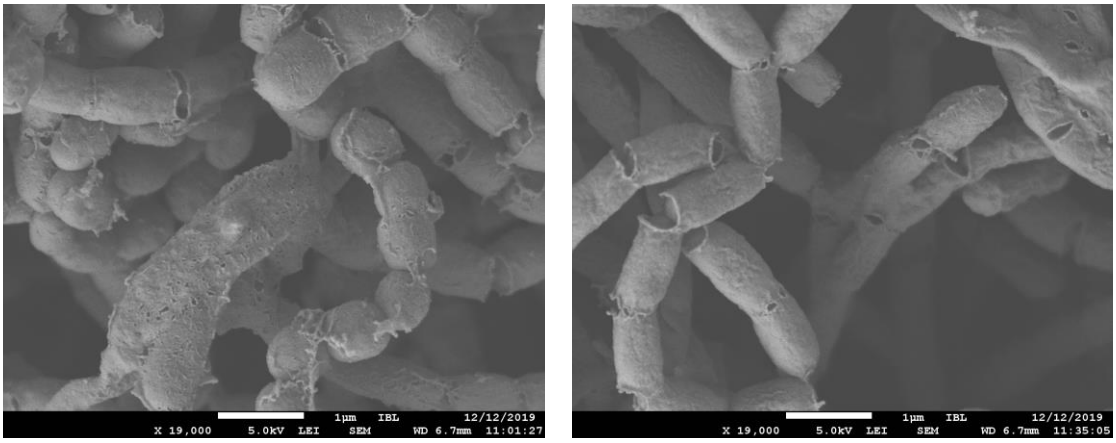

FtsZ-EE

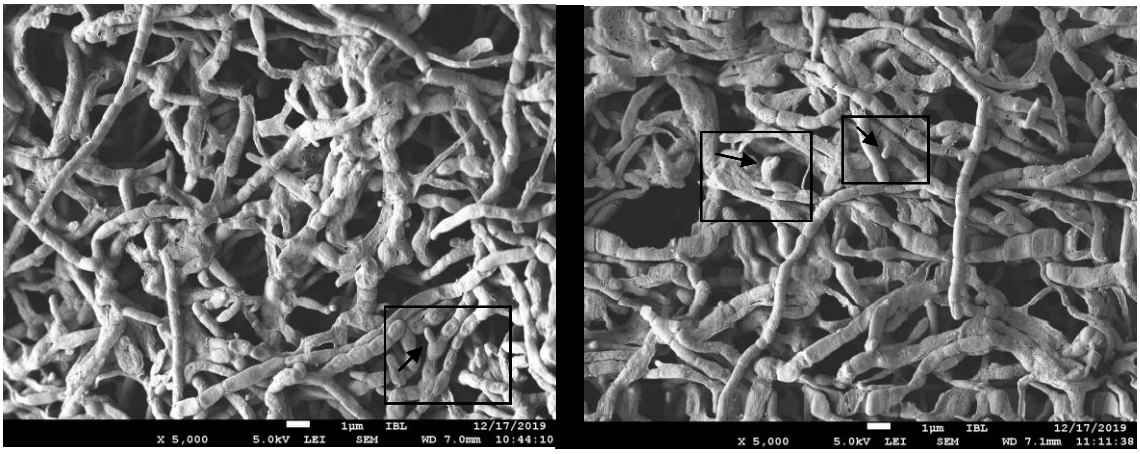

FtsZ-AA

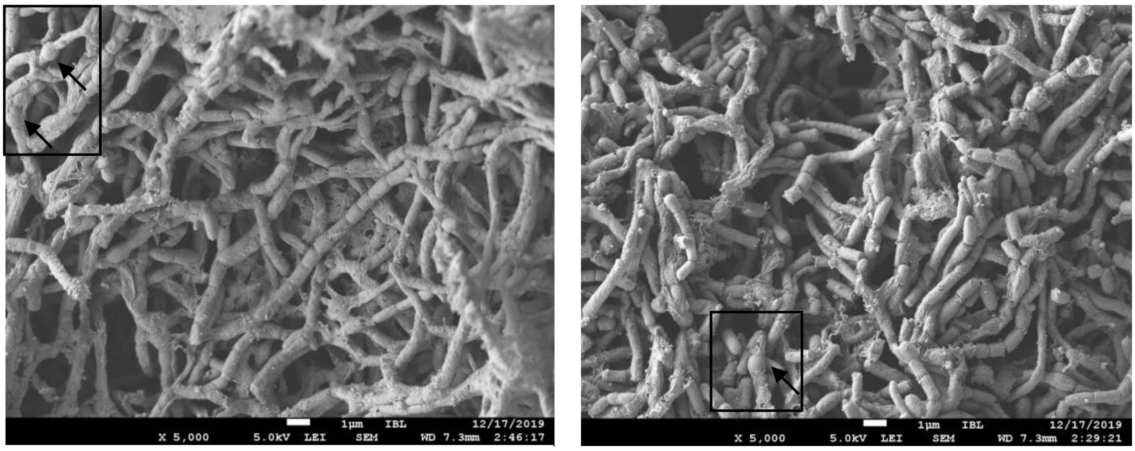

FtsZ-EA

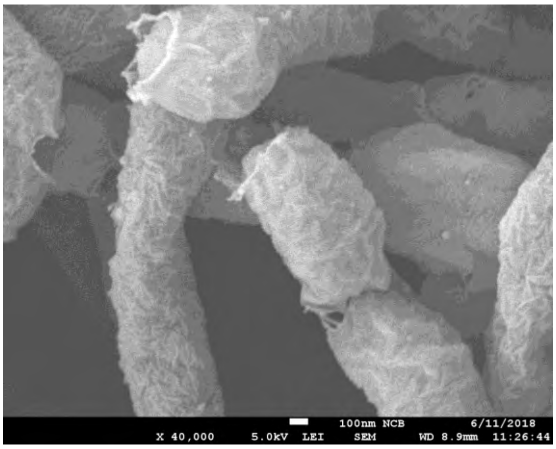

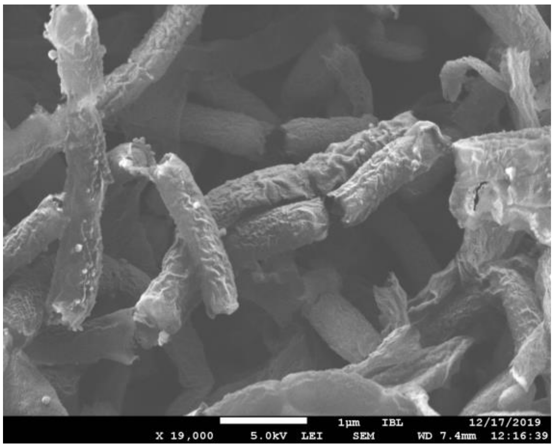

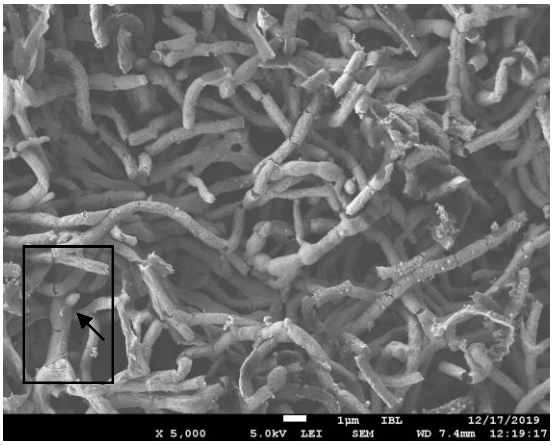

FtsZ-AE

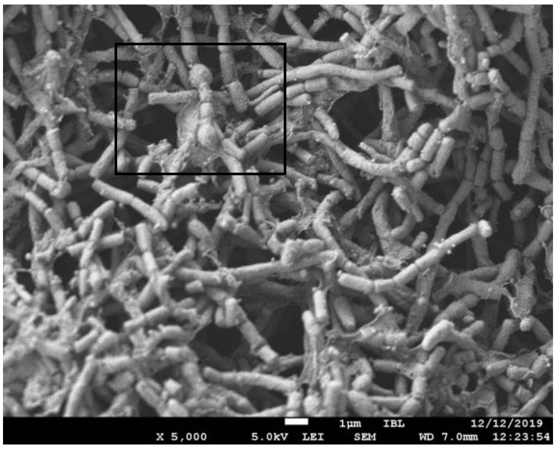

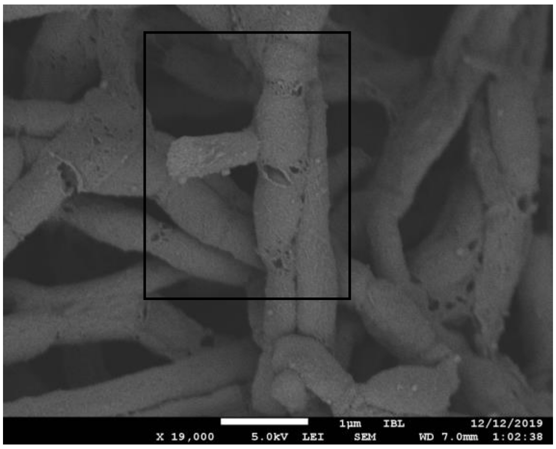

Supplementary figure 3. B) SEM micrographs of aerial hyphae in the mutants. Differences in thickness regarding FtsZ-AA can be observed.

WT

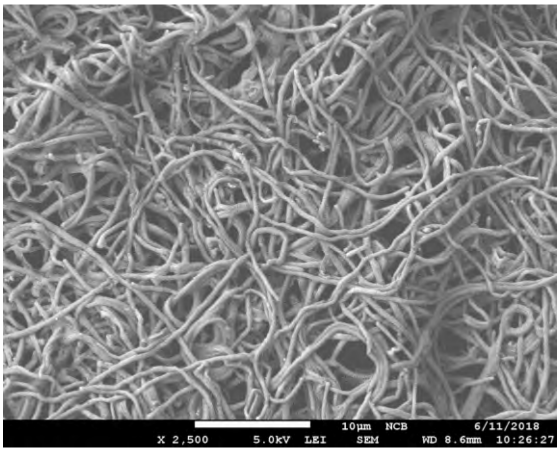

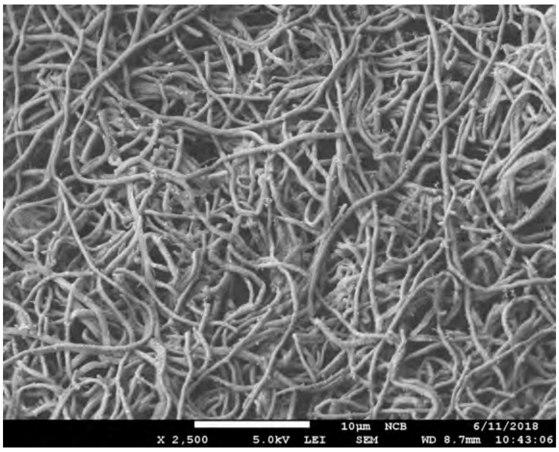

FtsZ-EE

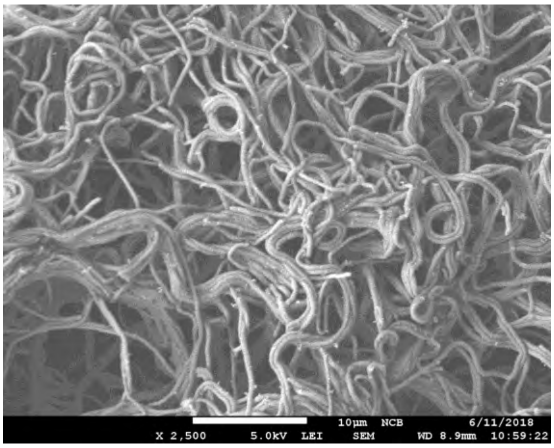

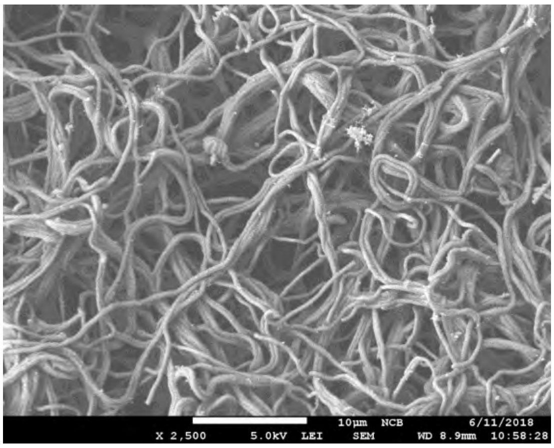

FtsZ-AA

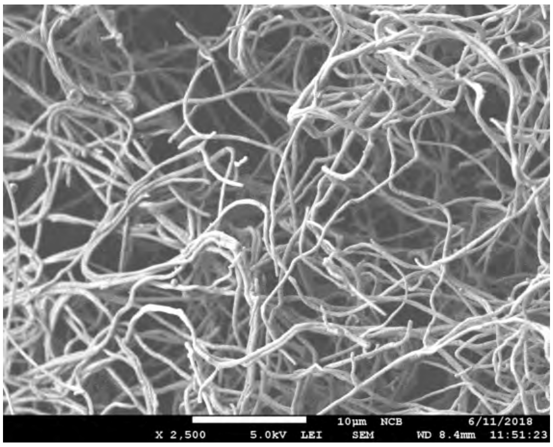

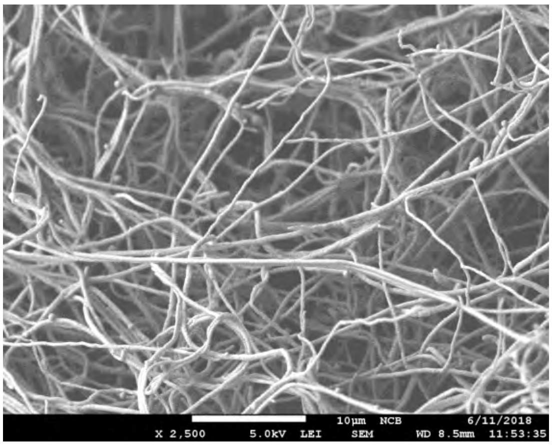

FtsZ-AA

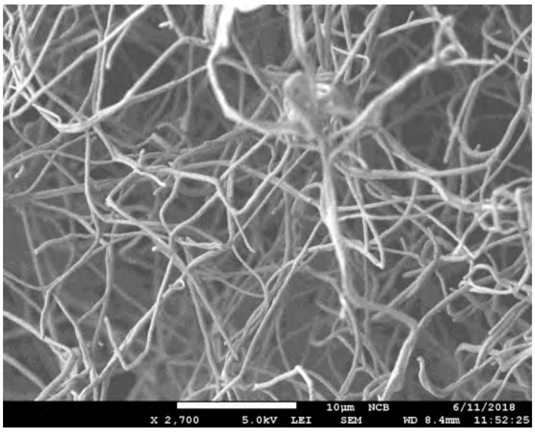

FtsZ-EA

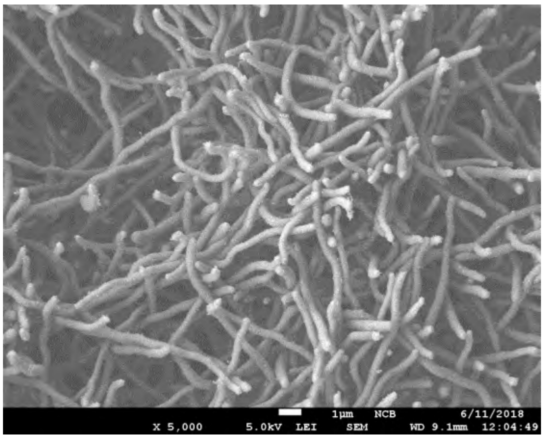

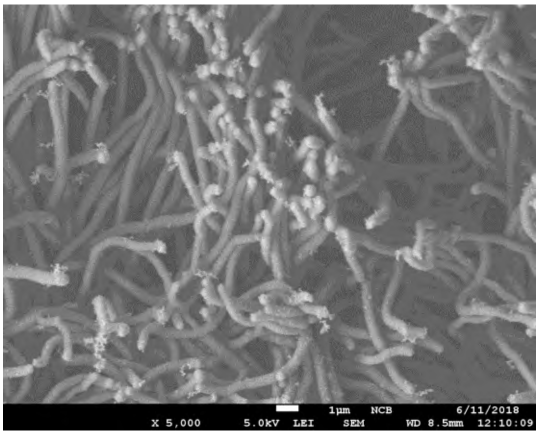

FtsZ-AE

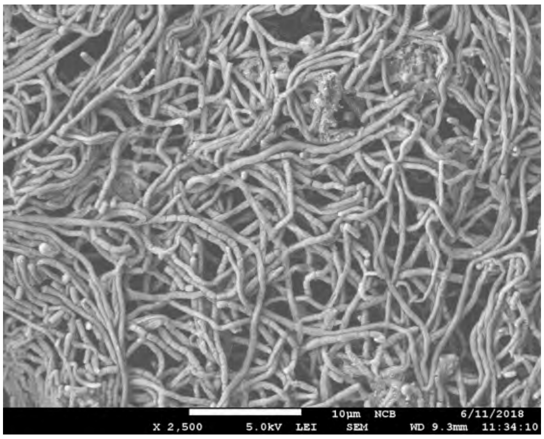

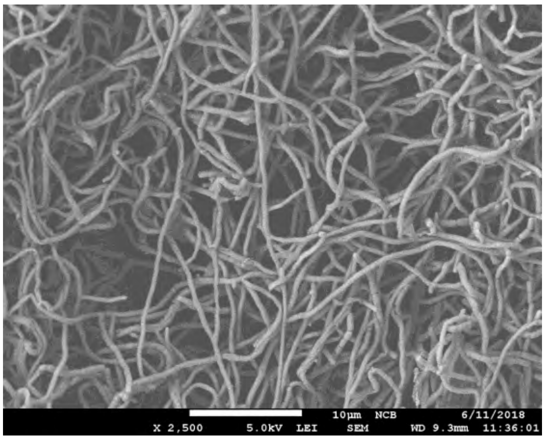

Supplementary figure 3. C) Uncropped TEM micrographs of spore chains

WT

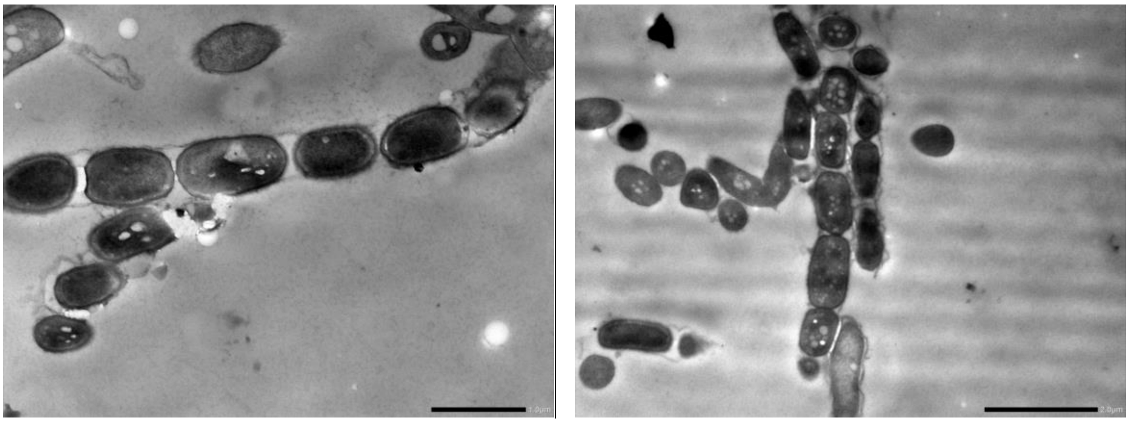

FtsZ-EE

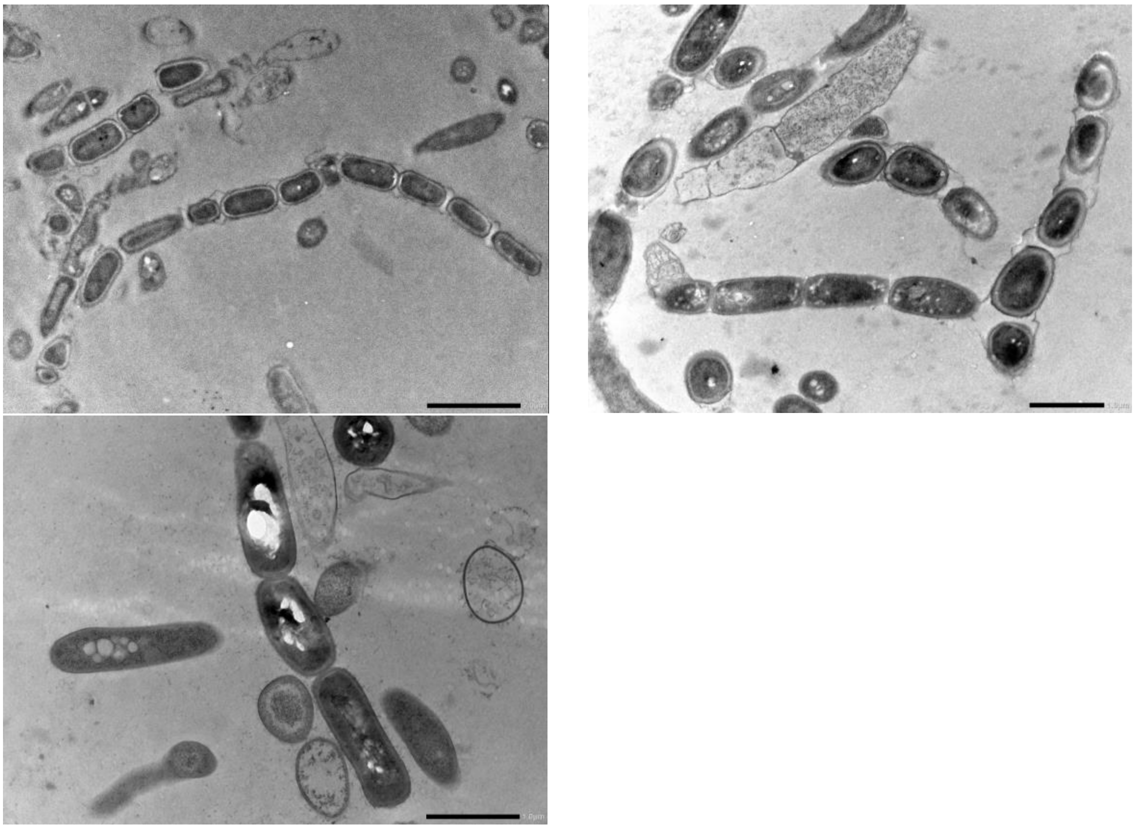

FtsZ-AA

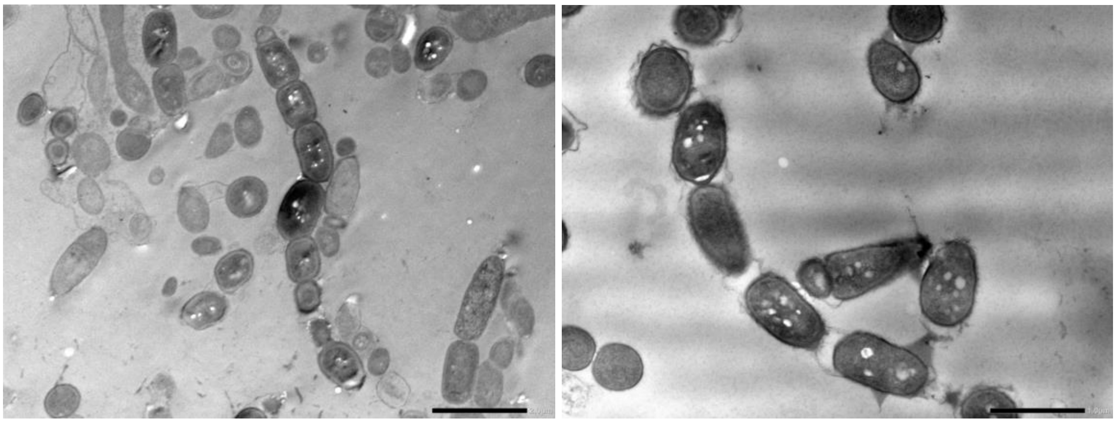

FtsZ-EA

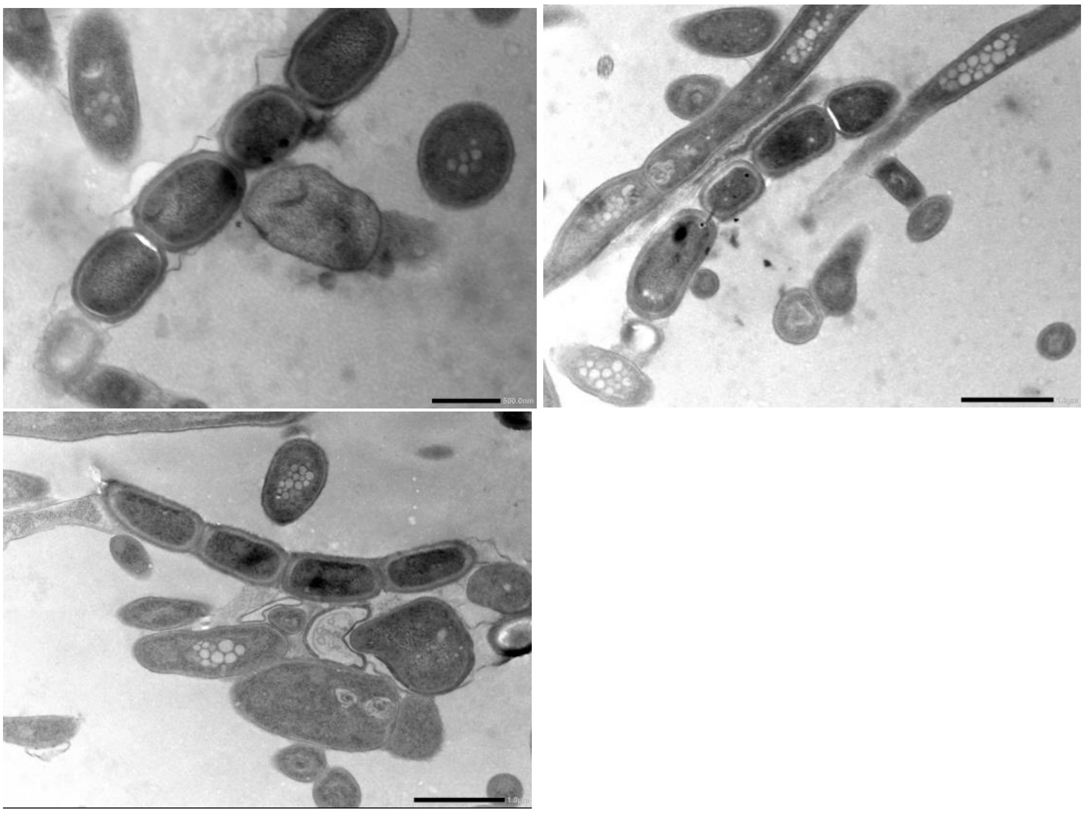

FtsZ-AE

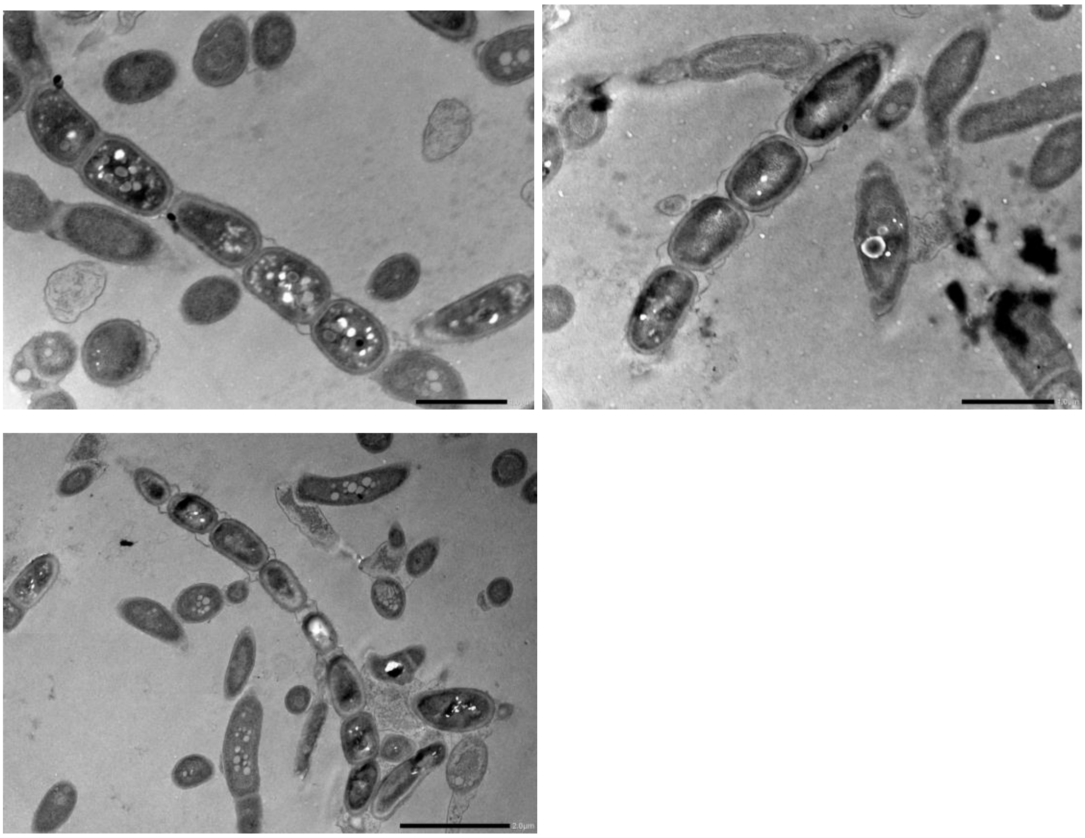

Supplement: Supplementary file 3 — Supplementary file3 (PDF 1624 KB) [file 10482_2022_1778_MOESM3_ESM.pdf]
