# Supplementary material for: FtsZ phosphorylation pleiotropically affects Z-ladder formation, antibiotic production, and morphogenesis in Streptomyces coelicolor
Source: Antonie Van Leeuwenhoek. 2022 Nov 16;116(1):1–19. doi: 10.1007/s10482-022-01778-w (PMC9823044; doi:10.1007/s10482-022-01778-w)

Supplementary figure 4. Confocal microscopy of 72 h growth in GYM solid cultures. Cultures were stained with the vital dyes SYTO9-Propidium Iodide.

WT

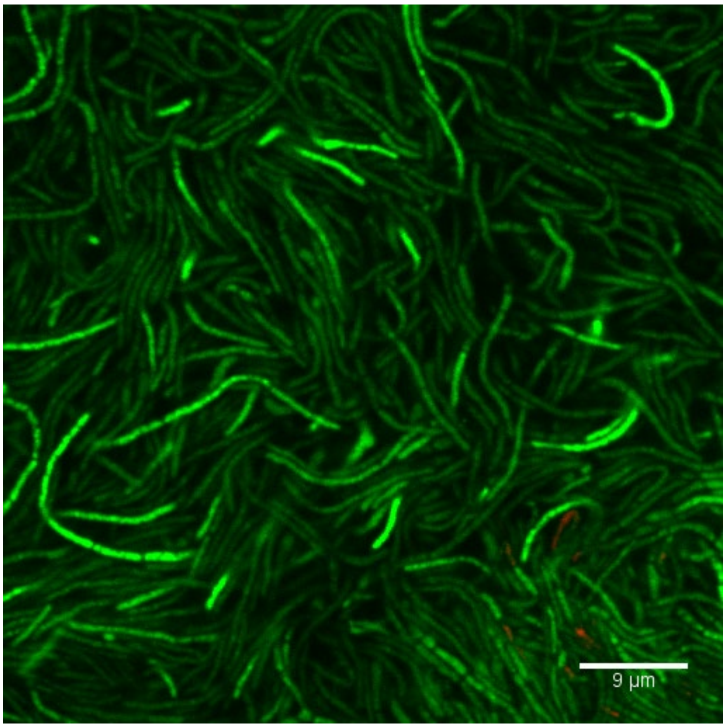

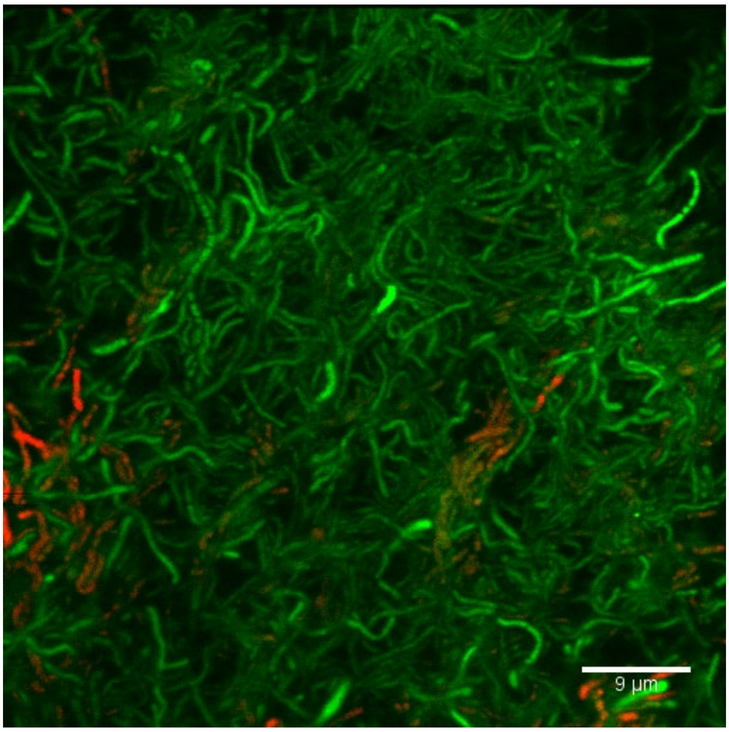

M1 (EE)

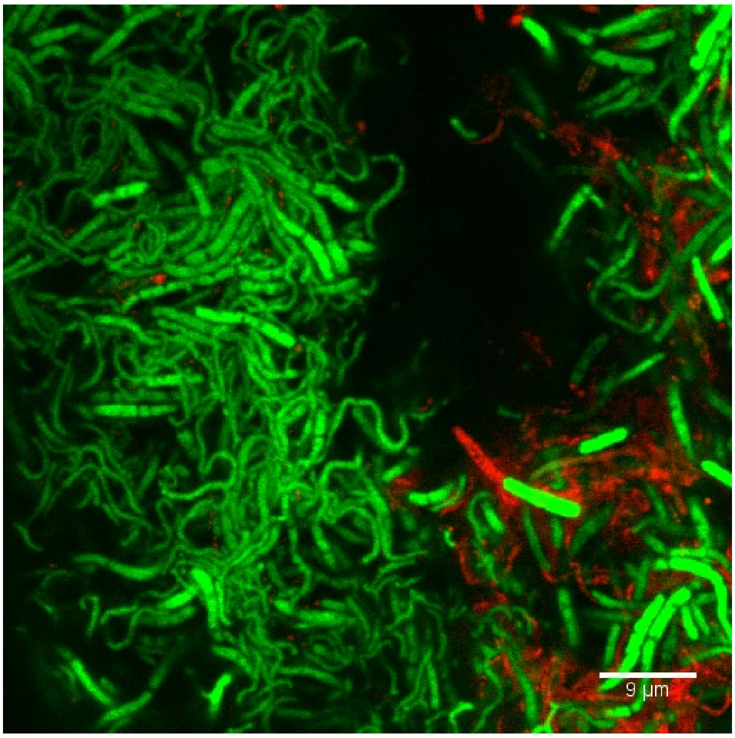

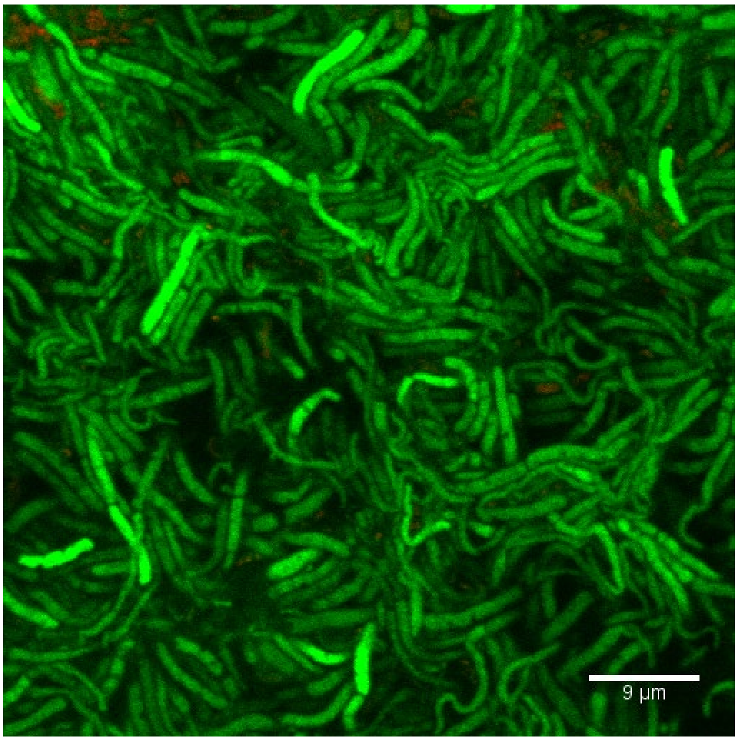

M2 (AA)

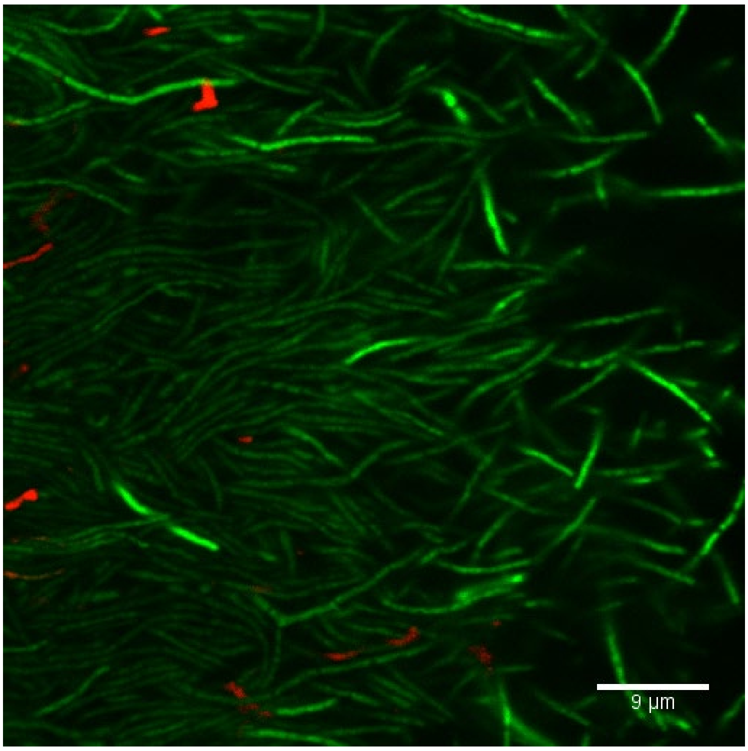

M3 (EA)

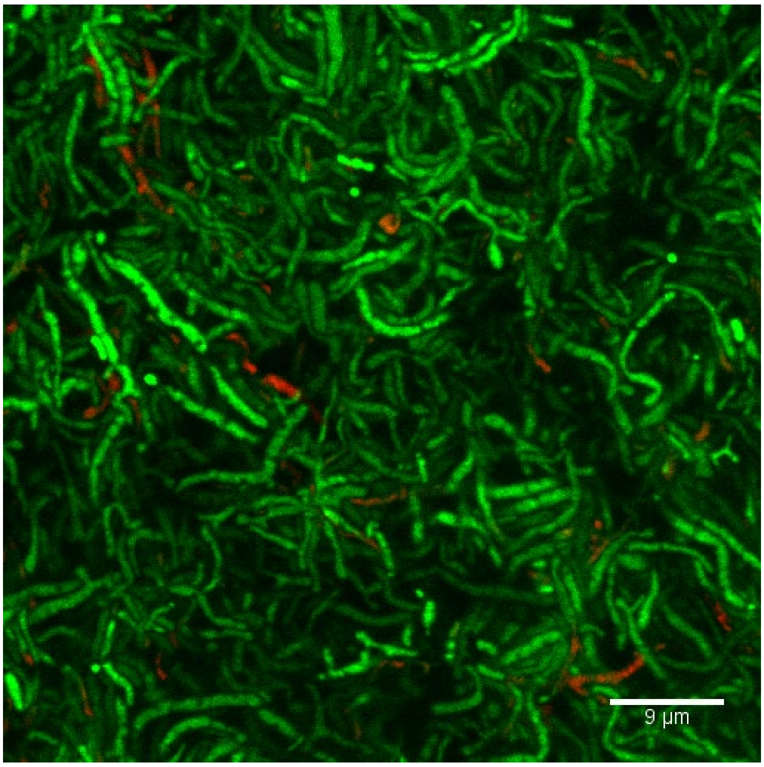

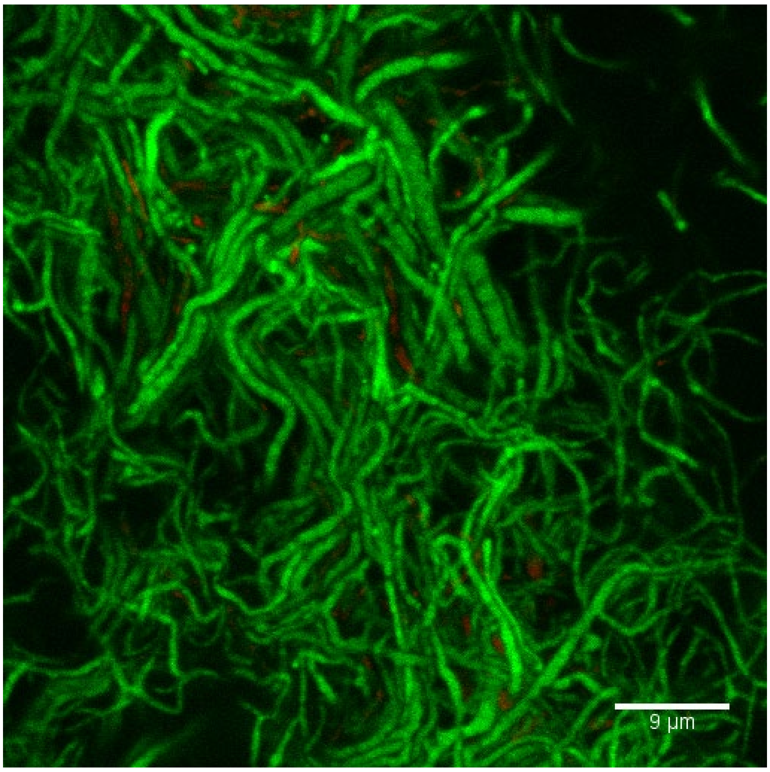

M4 (AE)

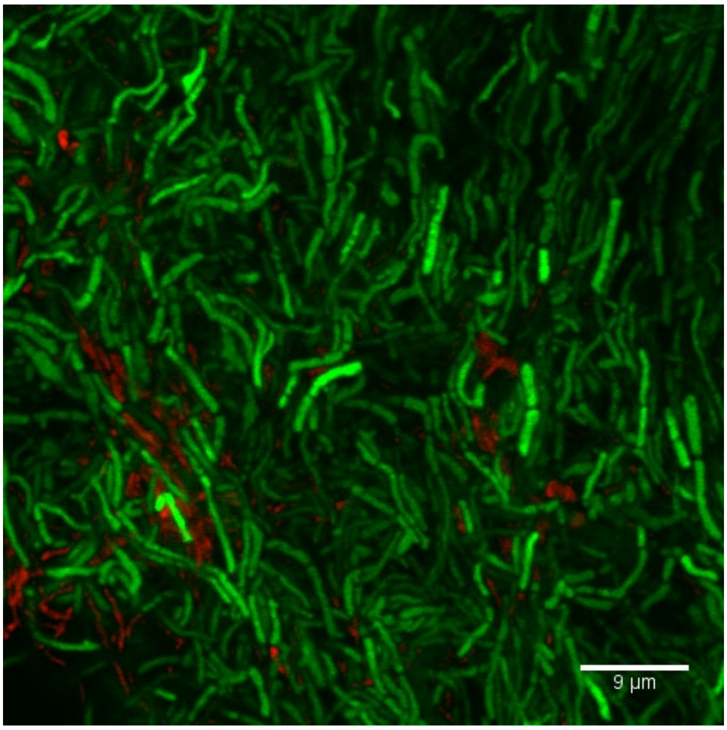

Supplement: Supplementary file 4 — Supplementary file4 (PDF 653 KB) [file 10482_2022_1778_MOESM4_ESM.pdf]
